# The pMTL70000 modular, plasmid vector series for strain engineering in *Cupriavidus necator* H16

**DOI:** 10.1016/j.mimet.2021.106323

**Published:** 2021-10

**Authors:** Muhammad Ehsaan, Jonathan Baker, Katalin Kovács, Naglis Malys, Nigel P. Minton

**Affiliations:** BBSRC/EPSRC Synthetic Biology Research Centre (SBRC), School of Life Sciences, Centre for Biomolecular Sciences, The University of Nottingham, Nottingham NG7 2RD, United Kingdom

**Keywords:** *Cupriavidus necator* H16, Modular vector, pMTL70000 series, Transformation, Segregational stability, Plasmid copy number

## Abstract

*Cupriavidus necator* H16 can convert CO_2_ into industrial chemicals and fuels. To facilitate its engineering, we designed, built and tested the pMTL70000 modular plasmids comprising standardised *Cupriavidus* and *E. coli* replicons, selectable markers and application specific modules. Plasmids were characterised in terms of transmissibility, stability, copy number and compatibility.

## Highlights

• A standardised, modular vector system for engineering *Cupriavidus necator* H16.
• An improved procedure for DNA transfer by electroporation.
• Vectors characterised in terms of segregational stability, copy number and compatibility.

*Cupriavidus necator* (formerly *Ralstonia eutropha*), is a non-pathogenic, Gram-negative, aerobic chemolithoautotroph. It is able to grow both autotrophically on CO_2_ as its sole carbon source and heterotrophically on variety of organic substrates, including fructose and gluconate. The potential to convert CO_2_ into a variety of polyhydroxyalkonates, chemicals and fuels (Tanaka et al., 1995; Li et al., 2012; Srinivasan et al., 2003; Bi et al., 2013; Grousseau et al., 2014; Chakravarty and Brigham, 2018) suggest that it can contribute to reducing greenhouse gas emissions. Accordingly, to improve on existing broad-host vectors (Bi et al., 2013; Sato et al., 2013; Gruber et al., 2014) we adopted a modular plasmid format, previously applied (Heap et al., 2009; Sheng et al., 2017) to Clostridium (pMTL80000) and Geobacillus (pMTL60000), to design, build and test a standardised modular vector system for strain engineering in *C. necator* H16. Details of material and methods used are provided in the Supplementary information.

**Fig. 1:**
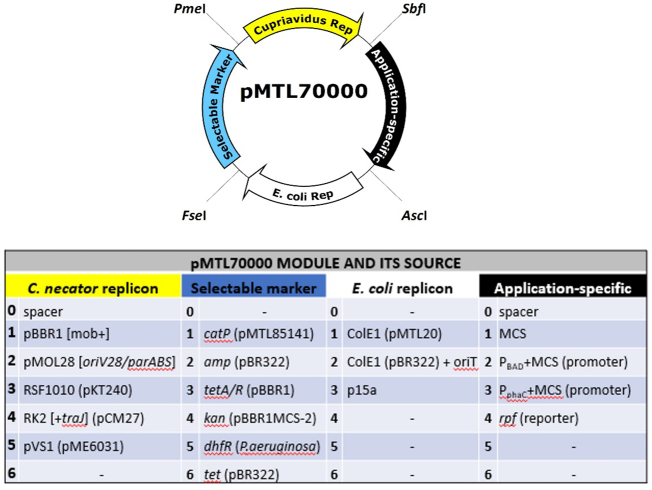
pMTL70000 series modular vectors with numbering scheme.

Each vector of the devised pMTL70000 system is divided into four modules flanked by the unique 8 bp recognition sites of the type II restriction endonucleases (RE) *Asc*I, *Fse*I, *Pme*I and *Sbf*I (Fig. 1). The modules, arranged in same order in all vectors, comprise a *C. necator* H16 replicon (PmeI/SbfI), an application specific module (SbfI/AscI), an *E. coli* replicon (AscI/FseI) and an antibiotic selection marker (FseI/PmeI). Individual modules were PCR amplified, digested with respective REs and ligated together to form a specific vector. Each module is allocated a number and their combination defines the vector name (Fig. 1). The base vector of the series, pMTL71101, was built using the *mob/rep* region from pBBR1, the *Clostridium perfringens catP* selectable marker and multiple cloning site (MCS) from pMTL85141 (Alagesan et al., 2018). Other vectors in the series were then built by exchanging individual modules with the available variants using appropriate flanking RE sites. These include alternative replicons, the selectable markers *tetA* (tetracycline), *amp*, (ampicillin) *kan* (kanamycin) and Dhfr (dihydrofolate reductase/trimethoprim resistance), a red fluorescence reporter protein (*rfp*) and two promoters, P_BAD_ and P_phaC._ A knockout vector (pMTL70621-SacB) was also constructed carrying the *sacB* counter selection marker. Through provision of the appropriate mutant alleles, the vector was subsequently used to delete genes encoding restriction enzymes by selecting cells able to grow in the presence of 15% (*w*/*v*) sucrose (see Supplementary Information).

**Fig. 2:**
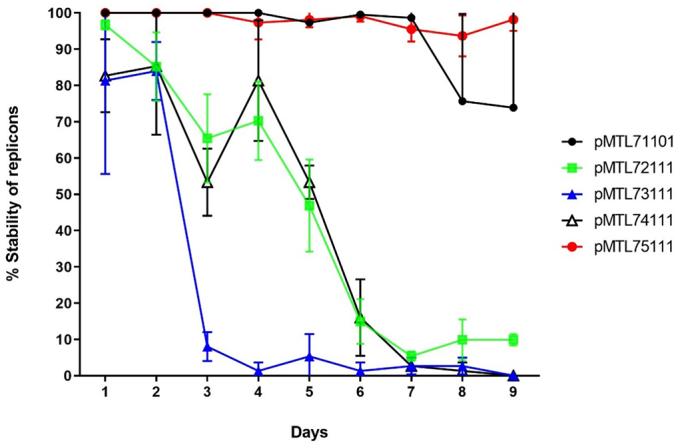
Segregational stability of the different replicons in *C. necator*. Plasmids assessed were pMTL71101, pMTL72111, pMTL73111, pMTL74111 and pMTL75111 containing the replicons pBBR1, pMOL28, RSF1010, RK2 and pVS1, respectively. Error bars represent standard deviations of three biological replicates.

The suitability of the various vectors and their replicons for engineering of *C. necator* H16 was tested as detailed in the Supplementary information, with respect to segregational stability, plasmid copy number and compatibility. Plasmid stability was assessed by estimating the number of cells retaining resistance to plasmid-encoded chloramphenicol after one to nine, 24-h serial passages at 30 °C in 0.4% (*w*/*v*) sodium gluconate-minimal medium (SG-MM) (Schlegel et al., 1961) lacking antibiotic supplementation. The percentage of cells retaining the plasmid was estimated by plating appropriately diluted cell culture on agar media and then replica plating single colonies onto agar media with and without chloramphenicol (100 μg/ml). Plasmids based on the *Pseudomonas* pVS1 replicon were found to most stable (Fig. 2), with more than 97% of the cells maintaining pMTL75111 over the nine days compared to only 9% and 74% in the case of pMTL72111 (pMOL28) and pMTL71101 (pBBR1), respectively. Plasmid pMTL73111 (RSF1010) was the most unstable, with 92% of cells losing the plasmid in just four days, in support of previous findings (Srinivasan et al., 2003; Sato et al., 2013).

Since gene dosage can be crucial for efficient protein production, it is desirable to know the range of copy numbers available in a vector series. Plasmid copy numbers were estimated by qPCR using primers directed against the *lacZα* region common to all plasmids and primers complementary to the chromosomal *panC* gene encoding pantothenate synthetase (see Supplementary information). Those plasmids with the highest copy numbers were based on pBBR1 and RK2, with 42.57 ± 2.00 and 42.10 ± 3.44 copies per cell, respectively. In contrast, plasmids carrying the pVS1 and pMOL28 replicons had respective copy numbers of 5.21 ± 0.24 and 6.03 ± 0.45). In other situations, it can be useful to divide heterologous genes between two different plasmids, which therefore need to co-exist. Compatibility studies were undertaken by identifying which plasmid pairs could simultaneously transform *C. necator* and be retained for at least 10-days following 5 subcultures (see Supplementary Information). This demonstrated that a plasmid carrying the pBBR1 replication region were relatively promiscuous, being able to co-exist in *C. necator* with plasmids based on the replicons of RK2, pMOL28 and RSF1010. Plasmids of the latter type were only compatible with vectors based on the IncP, RK2 replicon.

**Fig. 3:**
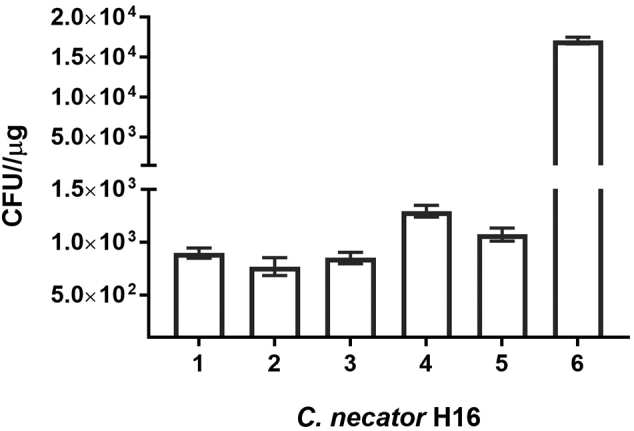
Effect of growth media and competent cell buffers on *C. necator* electroporation frequencies. Cells were transformed with 0.2 μg of pMTL71101 DNA extracted from *E. coli* DH5α. Key: 1- LB-0.3 M Sucrose, 2-LB-0.5 M Sucrose, 3-LB-1 mM HEPES, 4- SOB-0.3 M Sucrose, 5- SOB-0.5 M Sucrose, 6- SOB-1 mM HEPES.

Derivation and testing of the vectors was made possible by optimisation of the electroporation protocol used. A systematic comparison of different growth media, growth phase at which cells were harvested and the constituents of the electroporation buffer used, led to the most effective frequencies of DNA transfer (Fig. 3). The final method (Supplementary information) makes use of 1 mM HEPES buffer, which was more effective than either 0.3 M and 0.5 M sucrose solutions for electro-competent cell preparation and relied on cells grown in SOB rather than LB medium (Fig. 3). In these experiments, cells were harvested at a much earlier phase of growth than in the method reported by Xiong et al., 2018, being harvested at an OD_600_ of 0.2–0.3 rather than 0.6–0.8. Overall, the developed electro-competent cell preparation process takes less than 5-h, which is 2–3 h shorter than previously reported methodology (Tee et al., 2017).

It was previously reported (Xiong et al., 2018) that transformation frequency in *C. necator* H16 could be increased some 1658-fold following the disruption of the RE gene of a type I (H16_A0006) system and by 4-fold when a pair of adjacent type IV systems (H16_A0008 – H16_A0009) were deleted. The effects were not additive, as only a 1697-fold increase was seen when both systems were inactivated. Here, the increases in transformation frequencies over the wildtype were much more modest.

when the equivalent deletions were made using the knock-out vector pMTL70621-SacB (see Supplementary information), with only a respective 3- and 1.3-fold increase being observed with the equivalent (Little et al., 2019) type I (E6A55_RS00030) and type IV (E6A55_RS00040,E6A55_RS00045) deletion mutants, and a 16-fold increase when both were deleted. However, the final frequency obtained here (4.7 × 10^5^ per μg DNA) of the double mutant is 10-fold higher than that obtained in the study of Xiong et al. (2018) suggesting that the electroporation protocol used is more effective. Indeed, they reported frequencies as low as 2.5 × 10^1^ transformants per μg DNA with the wildtype compared to 1.6 × 10^4^ CFU/μg obtained here.

The pMTL70000 modular vector series and the optimised electroporation procedures described here, along with the recent optimisation of media components for cell growth (Azubuike et al., 2020) will facilitate the ongoing development of *C. necator* as an industrial chassis. Plasmids and their sequences are available at www.plasmidvectors.com.

## Funding

This work was supported by the 10.13039/501100000268Biotechnology and Biological Sciences Research Council [grant number BB/L013940/1] (BBSRC); and the 10.13039/501100000266Engineering and Physical Sciences Research Council (EPSRC) under the same grant number.

## Declaration of Competing Interest

The authors declare that they have no known competing financial interests or personal relationships that could have appeared to influence the work reported in this paper.

## Appendix A Supplementary data

Supplementary material Image 1
