# Supplementary material for: The pMTL70000 modular, plasmid vector series for strain engineering in Cupriavidus necator H16
Source: J Microbiol Methods. 2021 Oct;189:106323. doi: 10.1016/j.mimet.2021.106323 (PMC8482281; doi:10.1016/j.mimet.2021.106323)
Supplement: Supplementary file 1 — Supplementary material [file mmc1.docx]

**SUPPLEMENTARY INFORMATION**

**The pMTL70000 modular, plasmid vector series for strain engineering in *Cupriavidus* *necator* H16**

Muhammad Ehsaan, Jonathan Baker, Katalin Kovács, Naglis Malys, Nigel P. Minton^*^

*BBSRC/EPSRC Synthetic Biology Research Centre (SBRC), School of Life Sciences, Centre for Biomolecular Sciences, The University of Nottingham, Nottingham NG7 2RD, United Kingdom*

*Author to whom correspondence should be addressed. E-mail:

[nigel.minton@nottingham.ac.uk](mailto:nigel.minton@nottingham.ac.uk)

## Keywords:

*Cupriavidus* *necator* H16, modular vector, pMTL70000 series, transformation, segregational stability, plasmid copy number

# **MATERIALS AND METHODS**

# **Bacterial strains and media**

*C. necator* H16 (DSMZ-428, ATCC 17669) was purchased from the DSMZ (Braunschweig, Germany) and used in all experiments. *E. coli* DH5α (Grant et al. 1990) was used as the host for cloning and plasmid propagation, *E. coli* S17-1 was as donor strain to conjugate the plasmids into *C. necator* H16 (Simon et al. 1983). Strains are listed in **Table S1**. *C. necator* H16 and *E. coli* were generally grown in lysogeny broth (LB) at 30°C and 37°C, respectively. Where necessary, media was supplemented with antibiotics. Antibiotics used in *E. coli* were chloramphenicol (100 µg/ml in 70% ethanol), Tetracycline (10 µg/ml in 70% ethanol), ampicillin (100 µg/ml in distilled water), kanamycin (50 µg/ml in distilled water) and trimethoprim (10 µg/ml in 100% methanol). In *C necator* the antibiotics were chloramphenicol (100 µg/ml in 70% ethanol), Tetracycline (15 µg/ml in 70% ethanol) ampicillin (100 µg/ml in distilled water), kanamycin (100 µg/ml in distilled water) and trimethoprim (100 µg/ml in 100% methanol).

**Table S1**

| **Strain** | **Relevant features** | **Reference/source** |
| --- | --- | --- |
| *C. necator* H16 | (DSMZ-428, ATCC 17669) Wild type strain | DSMZ (Germany) |
| DH5α | F^-^ *endA1* *hsdR17* (rk^-^, mk_­_^+^) supE44 thi-1 λ^-^ *recA1 gyrA96* *relA1* *deoR* ∆(*lacZYA*-*argF*)- U169 φ80d*lacZ*∆M15 | (Grant et al. 1990) |
| *E. coli* S17-1 λpir | Conjugation donor. λ*pir* lysogen of strain S17-1 [*pro*, *res− hsdR17 (rK− mK+) recA−* with an integrated *RP4-2-Tc::Mu-Km::Tn7, Tp^r^*] | (Simon et al. 1983) |
| *E. coli* Stellar | F – ara, Δ(*lac-proAB*) [Φ80d *lacZΔM15*], r*psL(str), thi, Δ(mrr-hsdRMS-mcrBC), ΔmcrA, dam, dcm* | CLONTECH |

# **Chemicals and Oligonucleotide primers used in module construction**

Chemicals were sourced from Sigma-Aldrich unless stated. Restriction enzymes, T4 DNA ligase and alkaline phosphatase were purchased from either New England Bio labs (UK) or Thermo Fisher Scientific (UK). DreamTaq™ green PCR master mix (Thermo Fisher Scientific, UK) and Failsafe™ PCR system Cambio (UK) were used according to the manufacturer’s instructions for bacterial strain screening by PCR and amplification of DNA for cloning, respectively. DNA extraction and purification from agarose gel and PCR reaction mixtures were undertaken using QIAquick Gel Extraction and PCR Purification Kit (Qiagen Ltd UK), respectively, and used according to the manufacturer’s instructions. All the DNA manipulations including restriction digestion, dephosphorylation of 5′ end and ligation were carried out according to standard procedures as described previously (Ehsaan et al. 2016).

All oligonucleotide primers used in this study (Table S2a & b) were designed using the Primer3web (version 4.10) at <http://primer3.ut.ee> and synthesized by Eurofins Genomics (Ebersberg, Germany).

**Table S2a**

| **Primer** | **Sequence 5–3** | **Template** | **Reference** |
| --- | --- | --- | --- |
| Amp_F1 | ATATGGCGCGCCGGCCGGCCAGGTGGCACTTTTCGGGGAAATG | pTSA29 | Phillips (1999) |
| Amp_R1 | ATATGTTTAAACTCTGACGCTCAGTGGAACGAAAAC | pTSA29 | Phillips (1999) |
| Kan_F1 | ATATGGCGCGCCGGCCGGCCGGATGAATGTCAGCTACTGGGCTATC | pBBR1MCS-2 | Kovach et al. (1995) |
| Kan_R1 | ATATGTTTAAACTCAGAAGAACTCGTCAAGAAGGC | pBBR1MCS-2 | Kovach et al. (1995) |
| Tet_F1 | ATATGGCGCGCCGGCCGGCCGCAGGAGCGCCAGAAGGCCGCC | Tet^R^_,_ pME6000 | Lefebre & Valvano (2002) |
| Tet_R1 | ATATGTTTAAACCGCTCACAATTCCACACAACATAC | Tet^R^_,_ pME6000 | Lefebre & Valvano (2002) |
| TetA_F1 | ATATGGCCGGCCTAATTCTCATGTTTGACAGCTTATCATCG | pLO3 | Lenz & Friedrich (1998) |
| TetA_R1 | ATATGTTTAAACGGGTTGGTTTGCGCATTCACAGTTC | pLO3 | Lenz & Friedrich (1998) |
| mini_pVS1_F2 | ATGTTTAAACATGAAACCGCACCAGGACGGCCAG | pME6031 | Heeb et al (2000) |
| mini_pVS1_R1 | ATATCCTGCAGGAGCGCCGGCGGTCGAGTG | pME6031 | Heeb et al (2000) |
| dhfR_F1 | ATATGGCCGGCCTGACAGATGAGACAATAACCCTG | *P. aeruginosa* | Accession No. DQ522233 |
| dhfR_R1 | ATATGTTTAAACTCAGTTGATGCGTTCAAGCGCCG | *P. aeruginosa* | Accession No. DQ522233 |
| p15A_F1 | ATATGGCGCGCCAAGATGATCTTCTTGAGATCG | pMTL85111 | Heap et al. (2009) |
| p15A_R1 | ATATGGCCGGCCACTCGCTACGCTCGGTCGTTCG | pMTL85111 | Heap et al. (2009) |
| OriT_F1 AscI | ATATGGCGCGCCCGGTCAGTTCAGTAATTTCCTGC | pLO3 | Lenz & Friedrich (1998) |
| pBR322_R3 | ATATGGCCGGCCGGATCTAGGTGAAGATCCTTTTTG | pLO3 | Lenz & Friedrich (1998) |
| SacB_TetAF1 | GTGAATGCGCAAACCAACCCTTTATGGGATTCACCTTTATGTTG | pLO3 | Lenz & Friedrich (1998) |
| SacB_TetAR1 | CAACATAAAGGTGAATCCCATAAAGGGTTGGTTTGCGCATTCAC | pLO3 | Lenz & Friedrich (1998) |
| SacB_F3 | TCCCGGGATATGGGATTCACCTTTATGTTGATAAG | pLO3 *sacB* gene | Lenz & Friedrich (1998) |
| SacB_R3 | TGTTTAAACGATCCTTTTTAACCCATCACATATACC | pLO3 *sacB* gene | Lenz & Friedrich (1998) |
| AscI_ColE1_F1 | ATATGGCGCGCCTCCTTTTTGATAATCTCATGACCAAAATC | pMTL20 | Chambers et al. (1988) |
| Fse_ColE1_R1 | ATATGGCCGGCCGGGCCCTGCGTATTGGGCGCTCTTC | pMTL20 | Chambers et al. (1988) |
| *par*ABSF1 | AAAAGTTTAAACATGCACGCCTCAAAAGGACG | pMOL28 | Cupriavidus metallidurans CH34 |
| oriV28R1 | AAAACCTGCAGGCTTTCAGCGCAAAACACACCCT | pMOL28 | Cupriavidus metallidurans CH34 |
| RSF1010_F2 | TGTTTAAACTTCTTCAAATTCCCGTTGCACATAG | pKT240 | Badasarian et al. (1983) |
| RSF1010_R2 | AACCTGCAGGAGCAGAAGAGCATACATCTG | pKT240 | Badasarian et al. (1983) |
| pCM62F1 | AAAAGTTTAAACCGATACTGAGCGAAGCAAGT | pCM271rfp | Bi et al. (2013) |
| pCM62R1 | AAAACCTGCAGGCAGAAGTGGTCAGCTTGGCT | pCM271rfp | Bi et al. (2013) |
| mini_pVS1_F2 | ATGTTTAAACATGAAACCGCACCAGGACGGCCAG | pME6031 | Heeb et al (2000) |
| mini_pVS1_R1 | ATATCCTGCAGGAGCGCCGGCGGTCGAGTG | pME6031 | Heeb et al (2000) |
| M13F | GTAAAACGACGGCCAG | pMTL20 | Chambers et al. (1988) |
| M13R | CAGGAAACAGCTATGACC | pMTL20 | Chambers et al. (1988) |
| PanCFwd1 | GTGGTGATGAAGCTGTTCT | *C. necator* H16 | DSMZ-428 |
| PanCRev1 | CAAACTGCTGCACCATGC | *C. necator* H16 | DSMZ-428 |
| pCM271F | GTGTCGCTGCTGCACTGCTTCCGCGTCCTGGACCGTGG | pCM27 | Marx & Lidstrom (2001) |
| pCM271R | CCACGGTCCAGGACGCGGAAGCAGTGCAGCAGCGACAC | pCM27 | Marx & Lidstrom (2001) |
| pCM291F | GGTCCTGATCGACGAGGGAATCGTCGTGCTGTTTGC | pCM29 | Marx & Lidstrom (2001) |
| pCM291R | GCAAACAGCACGACGATTCCCTGGTCGATCAGGACC | pCM29 | Marx & Lidstrom (2001) |

**Table S2b**

| **Primer Name** | **Sequence 5–3** | **Targeted RM Locus tag from GenBank Accession Numbers** | |
| --- | --- | --- | --- |
|  |  | AM260479.1, AM260480.1, AY305378.1 | CP039287 CP039288, CP039289 |
| LHA1_F1 | ATATCCCGGGCGCGCAGTTTGATGAGAAGACGGATAAGC | H16_A0004-A0006 | E6A55_RS00020, E6A55_RS00025, E6A55_RS00030 |
| LHA1_SOE_R1 | CACCGGCGAAGGCATCGAATCACACGGGCATATGTAACTATGGGCGGATTCG | H16_A0004-A0006 | E6A55_RS00020, E6A55_RS00025, E6A55_RS00030 |
| RHA1_SOE_F1 | CGAATCCGCCCATAGTTACATATGCCCGTGTGATTCGATGCCTTCGCCGGTG | H16_A0004-A0006 | E6A55_RS00020, E6A55_RS00025, E6A55_RS00030 |
| RHA1_R1 | ATATTCTAGACTGGTGAAATAGTCAGCCATCGTTCTTTAG | H16_A0004-A0006 | E6A55_RS00020, E6A55_RS00025, E6A55_RS00030 |
| gyrB_SF1 | GGCTAAGTTGGCTGAGATGC | H16_A0004-A0006 | E6A55_RS00020, E6A55_RS00025, E6A55_RS00030 |
| A0008_SR1 | GTCACCTCGTAGGCCTTCTTCA | H16_A0004-A0006 | E6A55_RS00020, E6A55_RS00025, E6A55_RS00030 |
| LHA1A_F1 | ATATCCCGGGCTTGACGCGCGGCATCGACGCCA | H16_A0006 | E6A55_RS00030 |
| LHAA1_SOE_R1 | CCGGCGAAGGCATCGAATCACACGCCCATCAGGCGCTCCCTGCTTG | H16_A0006 | E6A55_RS00030 |
| RHAA1_SOE_F1 | CAAGCAGGGAGCGCCTGATGGGCGTGTGATTCGATGCCTTCGCCGG | H16_A0006 | E6A55_RS00030 |
| RHAA1_R1 | ATATCCCGGGCTGGTGAAATAGTCAGCCATCGTTCTTTAG | H16_A0006 | E6A55_RS00030 |
| H16_A0005_SF1 | TACTTGCGGAAATCCTCGACACTC | H16_A0006 | E6A55_RS00030 |
| A0008_SR1 | GTCACCTCGTAGGCCTTCTTCA | H16_A0006 | E6A55_RS00030 |
| LHA2_F1 | ATATTCTAGAACAAGCTGATGCAGGTGTGATTCGATGC | H16_A0008-A0009 | E6A55_RS00040,E6A55_RS00045 |
| LHA2_SOE_R1 | CTCATCCGATCAGACTTGGTCAGCCAGCCATCGTTCTTTAGATTCCTTCAAC | H16_A0008-A0009 | E6A55_RS00040,E6A55_RS00045 |
| RHA2_SOE_F1 | GGAATCTAAAGAACGATGGCTGGCTGACCAAGTCTGATCGGATGAG | H16_A0008-A0009 | E6A55_RS00040,E6A55_RS00045 |
| RHA2_R1 | ATATTCTAGAGCGGAGCCTTCGTTTTTCAACGCCTC | H16_A0008-A0009 | E6A55_RS00040,E6A55_RS00045 |
| H16_A0006_SF1 | GCGTCCACCGCTGTTAGATGC | H16_A0008-A0009 | E6A55_RS00040,E6A55_RS00045 |
| H16_A0011_SR1 | CGGGTCTTCAACGATTCCCTGA | H16_A0008-A0009 | E6A55_RS00040,E6A55_RS00045 |
| LHA3_F1 | ATATCCCGGGCCATGGTAGATCACAGGATTTCCAAG | H16_A0014 | E6A55_RS00070 |
| LHA3_SOE_R1 | CGATGTCGAAAGAAAGCCGATGCAAGATTGAACGCAAGAGCTGGAGATTAC | H16_A0014 | E6A55_RS00070 |
| RHA3_SOE_F1 | GTAATCTCCAGCTCTTGCGTTCAATCTTGCATCGGCTTTCTTTCGACATCG | H16_A0014 | E6A55_RS00070 |
| RHA3_R2 | ATATTCTAGACCTTGGGGGATGGTGTCGCTCTGT | H16_A0014 | E6A55_RS00070 |
| H16_A0013_SF1 | AGTAATCCTGCTCGCTTTGGATG | H16_A0014 | E6A55_RS00070 |
| H16_A0016_SR1 | GCAAGGCCACTTCTTCTGTCCG | H16_A0014 | E6A55_RS00070 |
| LHA4_F1 | ATATTCTAGACAGAACGAAACTTGGAACTCCCGTTTC | pHG170 | E6A55_RS33050 |
| LHA4_SOE_R1 | GATCGAGCCGGTTCCTAGACTAGACACCCATTTCTCTACCTGTTGTTGTTCTTC | pHG170 | E6A55_RS33050 |
| RHA4_SOE_F1 | GAAGAACAACAACAGGTAGAGAAATGGGTGTCTAGTCTAGGAACCGGCTCGATC | pHG170 | E6A55_RS33050 |
| RHA4_R1 | ATATTCTAGAGGCTACTTCGTAGTCCATGCCGTTC | pHG170 | E6A55_RS33050 |
| pHG169_SF1 | CTGCAACATTCGGGAACAAGATTC | pHG170 | E6A55_RS33050 |
| pHG170_SR1 | AGCCTAGTCATCCAAAGCGCTC | pHG170 | E6A55_RS33050 |

**Preparation of *C. necator* H16 competent cells and electroporation**

Chemically competent *E. coli* cells were prepared as described previously (Ehsaan et al. 2016). To prepare *C. necator* H16 electro-competent cells, a seed culture was streaked onto Luria broth (LB) agar plates and incubated overnight at 30^o^C. Next day, 10 mL of Hanahan’s Broth (SOB Medium- H8032 Sigma) was inoculated with a heavy loop of freshly grown *C. necator* H16 cells. After re-suspending thoroughly, serial dilutions of 10^-1^, 10^-2^ and 10^-3^ were prepared and incubated aerobically at 30^o^C and 250 rpm overnight. The following morning, 50 mL of SOB broth was dispensed into a 250 mL baffled flask, inoculated with one of the overnight cultures in mid- or late-logarithmic growth phase to a starting OD_600_ of 0.055-0.075 and incubated at 30^o^C and at 250 rpm for approximately 2 hr until the culture reached an OD_600_ of 0.25 to 0.3. The cells were pelleted using 50 mL Falcon tubes, with 25 mL of the culture in each, using a pre-cooled benchtop centrifuge at 7000 rpm for 10 min at 4^o^C and the supernatant discarded. Cells were resuspended in the 10 mL of buffer A (1 mM HEPES, pH 7.0; adjusted with NaOH, pre-chilled and filter-sterilized), and centrifuged as before. After the supernatant was discarded, cells were washed again with 5 mL of buffer A and centrifuged as before. The pellet was re-suspended in buffer A supplemented with 10% (w/v) of glycerol to an OD_600_ of 5. This electro-competent cell suspension was then frozen in aliquots of 100 µl and stored at -80^o^C.

Electroporation was performed as follows. A 100-300 ng aliquot of plasmid was mixed with 100 µl of cells and transferred to a pre-chilled electroporation cuvette of 0.2-cm width gap and incubated on ice for 2 to 5 min. Electroporation was performed using a Gene Pulser (Bio-Rad, UK) and the following settings: 2.5 kV, 200 Ω and 25 µF. Immediately after electroporation, 0.9 mL of SOC medium was added to the cell suspension in the electroporation cuvette and the cells transferred into a 50 mL Falcon tube and incubated at 30^o^C and 250 rpm for 2 hr. Undiluted or serially diluted cells were spread on LB agar plates, supplemented with appropriate antibiotics, and incubated at 30^o^C for at least 24 hr or until colonies had appeared.

**Construction of modular vectors**

The base vector of the series, pMTL71101, was built using the *mob/rep* region from pBBR1*, the *catP* antibiotic selection marker and multiple cloning site (MCS) from pMTL85141 (Alagesan et al. 2018). Other vectors in the series were then built by exchanging individual modules with the various available alternatives using the two appropriate flanking RE enzymes. Alternative *Cupriavidus* replicons to that of pBBR1* were those of pMOL28, RSF1010, RK2 and pVS1. These were PCR amplified using the Failsafe™ PCR system (Cambio UK) from pMOL28 (from *C. metallidurans* CH34, DSMZ 2839), pKT240, pCM271rfp (Bi et al. 2013) and pME6031 using oligonucleotide primer pairs parABSF1/oriV28R1, RSF1010_F2/RSF1010_R2, pCM62F1/pCM62R1 and mini_pVS1_F2/ mini_pVS1_R1, respectively, PCR amplified products were then digested with SbfI and PmeI and the treated DNA ligated with similarly cleaved pMTL70111 to yield pMTL72111, pMTL73111, pMTL74111 and pMTL75111, respectively.

Other selectable markers to *catP* were *tetA* (tetracycline), *amp*, (ampicillin) *kan* (kanamycin) and Dhfr (dihydrofolate reductase, conferring resistance to trimethoprim). Marker genes were PCR amplified with Failsafe™ PCR system (Cambio UK) using oligonucleotide primer pairs of Amp_F1/Amp_R1, TetA/R_F1/TetA/R_R1, Kan_F1/Kan_R1, and dhfR_F1/dhfR_R1 from vector templates pTSA29, pBBR1MCS-2, pBBR1, *P. aeruginosa* (accession number DQ522233) genomic DNA and after separating on agarose gel and purification, digested with restriction enzymes FseI and PmeI and ligated to pMTL71101 linearized with same set of restriction enzymes resulting in plasmids pMTL71201, pMTL71301, pMTL71401 and pMTL71501.

Modules carrying *E. coli* replication regions were based on those of pMTL20 or pBR322 (both ColE1-based) or p15A. They were incorporated by deleting the pBBR1* replicon from the pMTL71101 by the quick change method using the oligonucleotide primer pair of 70111_F1/70111_R1 followed by digestion of the fragment with AscI /FseI and ligation with the ColE1 (high and medium copy number versions) and p15A replicons amplified with oligonucleotide primer pairs of AscI_ColE1_F1/ Fse_ColE1_R1, OriT_F1 AscI/pBR322_R3 and p15A_F1/p15A_R1 using the respective template DNA of pMTL20, pOL3 and pMTL85111. Following PCR, the DNA amplified was digested with AscI and FseI and ligated with pMTL71101 linearized with the same restriction enzymes resulting in plasmids pMTL70111, pMTL70621 and pMTL70131, respectively.

A knockout vector was constructed based on the counter selection marker *sacB.* DNA encompassing this gene was fused to DNA encoding *tetA* marker by splicing over extension (SOEing) using oligonucleotide primer pairs of TetA_F1/SacB_TetAR1 and SacB_TetAF1/SacB_R3 in the first round of PCR and TetA_F1/SacB_R3 in the second round. Following amplification of the final product it was digested with FseI and PmeI and ligated with pMTL70621 DNA that had been cleaved with the same restriction enzymes. The plasmid generated was designated pMTL70621-SacB.

All newly constructed plasmids were verified by Sanger sequencing (Eurofins Genomics, Ebersberg, Germany). The sequences of the modular parts and selected vectors are given in the Appendix. These may be sourced from [www.plasmidvectors.com](http://www.plasmidvectors.com) in *.gb format, as can the vectors themselves.

## **Determination of plasmid segregational stability**

To estimate the segregational stability in *C. necator* H16, plasmid-containing strains were streaked out onto 0.4% (w/v) sodium gluconate-minimal medium (SG-MM) agar plates (Schlegel et al. 1961) supplemented with 100 µg/ml chloramphenicol and incubated overnight at 30^o^C. Single colonies from agar plates were inoculated into liquid SG-MM media supplemented with 100 µg/ml chloramphenicol and incubated overnight at 30^o^C with shaking, 200 rpm. Then, 10-ml aliquot of SG-MM media was inoculated with the overnight culture to an OD_600_ of 0.2 and incubated at 30^o^C and 200 rpm for 24 hr. Cells from this culture were used to inoculate a fresh SG-MM liquid media and the culture incubated as before. This procedure was repeatedly every 24 hr, at 24, 48, 72 and 96, 120, 144, 168, 192 and 216 hr time points. At each 24 hr time point, samples were taken from cultures and after dilution plated out on LB agar, which were then incubated at 30^o^C for 48 hr. In a subsequent step, the colonies were replica plated from LB agar plates to LB plates with and without supplemented with 100 µg/ml chloramphenicol. For all agar plates, the number of colonies forming units (CFU) was calculated. The ratio of CFU that grew on LB selective and LB non-selective plates at each time point revealed the percentage of plasmid stability over a time period of 216 hr. Plasmid stability assays were performed in triplicate.

## **Determination of plasmid copy number in *C. necator* H16**

Plasmid copy number was determined by comparing the quantification signal from the plasmid to those from the chromosome. The two primer pairs PanCFwd1/PanCrev1 and M13F/M13R (Table S2a) were designed to respectively target a single gene in the chromosome (*panC*, encoding pantothenate synthetase) and a conserved domain on the plasmid (*lacZa*)*.* The predicted sizes of the fragments that would be amplified were 103 and 153 bp, respectively. Plasmid containing cell lines were grown overnight in LB liquid media supplemented with an antibiotic appropriate to the plasmid and plated onto LB agar supplemented with the same antibiotic. Agar plates were incubated at 30^o^C for 48 hr and developed single colonies were re-suspended in 10 µl of nuclease free water, boiled for 10 min, centrifuged at full speed for 1-2 min before decanting the supernatants to be used as the template in qPCR.

PCR reactions were performed in a 20 µl reaction volume with Rotor-Gene^TM^ 3000 (Corbett Research) using LuminoCt SYBR green qPCR ReadyMix (Sigma UK) according to manufacturer instructions. The concentration of the DNA was measured by (SimpliNano) and copy number was calculated using the following equation (Whelan et al. 2003):

$$N_{DNA}=N_{A}C_{DNA}$$

(1)

Were *N_DNA_* is plasmid DNA copy number; *N_A_* – Avogadro constant and *C_DNA_* – plasmid DNA molar concentration.

A 10-fold serial dilution of plasmid and genomic DNA ranging from 1 x 10^5^ to 1 x 10^9^ copies/µl was used to construct the standard curves for both *panC* and *lacZa*. Threshold cycle (C_T_) values in each dilution were measured in triplicate using a real-time qPCR with sets of oligonucleotide primers for *panC* and *lacZa* and plotted against the logarithm of the initial copy number to generate the standard curves for *panC* and *lacZa*, respectively, and linear regression analysis. PCR amplification efficiency of each standard curve was calculated according to the following equation (Rasmussen 2001).

$$E={10}^{-1/slope}$$

(2)

Relative quantification of copy number of each replicon was carried out using efficiency-corrected ∆Cp method (Rao et al. 2013, Bilgrau et al. 2016).

## **Determination of replicon compatibility in *C. necator* H16**

*C necator* H16 electrocompetent cells (100 μl) were simultaneously transformed with 100 ng of two plasmids that carried different *C. necator* replicons and either the antibiotic selection marker *catP* or *tetA/R*. Transformed cells were recovered for 4 hr and plated on LB agar supplemented with either Cm (100 µg/ml) or Tet (15 µg/ml).  Transformant colonies appeared after 48 hr. The presence of both plasmids was confirmed by screening for their presence using colony PCR and a pair of replicon-specific primers (parABSF1 and oriV28R1, RSF1010_F2 and RSF1010_R2, mini_pVS1_F2 and mini_pVS1_R1, pCM271F and pCM271R – see Table S2a for sequences). The products of PCR were electrophoresed on an agarose gel to allow an assessment of whether a DNA fragment of a size appropriate to the replicon being screened was present or absent.  Transformants were sub-streaked every 48 hr from a single colony on LB agar supplemented with antibiotics for a period of 10 days and the presence of both replicons was confirmed by colony PCR. In no case was a plasmid seen to be lost.

**Gene deletion protocol in *C. necator* H16 using pMTL70621-SacB**

Knock-out plasmids based on pMTL70621-SacB were introduced into *C. necator* H16 via conjugation with *E. coli* S17-1 (Simon et al. 1983). Donor cells were grown in 5 mL LB, supplemented with appropriate antibiotic, at 37°C. The recipient (*C. necator* H16) was cultured in 5 mL YLB at 30°C overnight. A 1 mL aliquot of both donor and recipient cultures were centrifuged for 4 min at 5000 rpm, cell pellets were resuspended in 1 mL of LB each and centrifuged as before. Both recipient and donor cells were each resuspended in 500 µL LB, mixed together and centrifuged as before. The supernatant was discarded, the pellet was resuspended in 50 µL of LB and cell mixture was spotted onto LB agar and incubation at 30°C for 6 hr. Thereafter, cells were resuspended in 500 µL of Phosphate Buffer Saline (PBS) and plated on 0.4% (w/v) fructose minimal media (0.4% F-MM) (Schlegel et al. 1961) supplemented with 15 µg/mL tetracycline and incubated at 30°C for 48 hr. *C. necator* H16 transconjugants were picked and purified on 0.4% (w/v) F-MM supplemented with 15 µg/mL tetracycline by re-streaking twice. Single crossover between plasmid and chromosomal DNA was confirmed by colony PCR using an appropriate pair of screening oligonucleotide primers. *C. necator* H16 strains confirmed for single crossover were then inoculated into modified LB containing 0.25% (w/v) NaCl Low Salt Luria Broth (LSLB medium) (Lenz and Friedrich 1998) without any supplementation and grown overnight. Dilutions were made in PBS and 100 µL of each plated on LSLB-agar supplemented with 15% (w/v) sucrose and incubated at 30°C for 48 hr to select for sucrose resistant colonies, ie., the double crossover. Colony PCR was performed on sucrose resistant colonies using a flanking pair of oligonucleotide primers to screen for the double crossover deletion mutants.

## **Construction of restriction endonuclease mutant strains of *C. necator* H16**

The KO cassette targeting the E6A55_RS00020, E6A55_RS00025, E6A55_RS00030, as designated in Little et al. (2019), (equivalent to H16_ A0004-A0006 in Xiong et al., 2018) was composed of bases 6216 to 6965 (LHA) of the *C. necator H16* chromosome 1 fused to bases 14043 to 14792 (RHA) and was designed to delete a 7077bp DNA fragment sequence. The oligonucleotide primer pairs LHA1_F1/LHA1_SOE_R1 and RHA1_SOE_F1/RHA1_R1 were designed to position an XmaI restriction recognition site adjacent to the LHA and an XbaI site at the extremity of the RHA. The cassette was isolated as a 1500 bp XmaI/XbaI fragment and inserted between the XmaI and XbaI sites of pMTL70621-SacB to yield the KO plasmid pMTL70621-SacB::H16_A0004-A0006.

The KO cassette targeting only E6A55_RS00030 (H16_A0006) gene was composed of bases 9984 to 10733 (LHA) of the *C. necator* H16 chromosome 1 (PCR amplified using primer pair LHA1A_F1/LHAA1_SOE_R1) fused to bases 14043 to 14792 (RHA) (PCR amplified using primer pair RHAA1_SOE_F1/RHAA1_R1) by splicing over-extension PCR and was designed to delete 3309 bp of E6A55_RS00030. The PCR primers used were designed to position an XmaI restriction endonuclease site adjacent to the LHA and an XmaI site at the extremity of the RHA. The cassette was isolated as a 1500 bp XmaI/XmaI fragment and inserted at the XmaI site of pMTL70621-SacB to yield the KO plasmid pMTL70621-SacB::H16_A0006.

The KO cassette targeting only E6A55_RS00040, E6A55_RS00045 (equivalent to H16_A0008-A0009 in Xiong et al., 2018) genes was composed of bases 14029 to 14778 (LHA) of the *C. necator* H16 chromosome 1 (PCR amplified using primer pair LHA2_F1/LHA2_SOE_R1) fused to bases 18507 to 19256 (RHA) (PCR amplified using primer pair RHA2_SOE_F1/RHA2_R1) by splicing over-extension PCR and was designed to delete 3728 bp of in E6A55_RS00040, E6A55_RS00045. The PCR primers used were designed to position an XbaI restriction endonuclease site adjacent to the LHA and at the extremity of the RHA. The cassette was isolated as a 1500 bp XbaI/XbaI fragment and inserted at the XbaI site of pMTL70621-SacB to yield the KO plasmid pMTL70621-SacB::H16_A0008-09. This KO plasmid was used to make deletion in wild type *C. necator* as H16_ A0008-09 and also KO in strain H16ΔH16_RS00030 to make H16ΔH16_ RS00030ΔH16_ RS00020-25.

The KO cassette targeting only E6A55_RS00070 (equivalent to H16_A0014, Xiong et al., 2018) genes was composed of bases 21692 to 22441 (LHA) of the *C. necator* H16 chromosome 1 (PCR amplified using primer pair LHA3_F1/LHA3_SOE_R1) fused to bases 23546 to 24295 (RHA) (PCR amplified using primer pair RHA3_SOE_F1/RHA3_R2) by splicing over-extension PCR and was designed to delete 1104 bp of E6A55_RS00070. The PCR primers used were designed to position an XmaI restriction endonuclease site adjacent to the LHA and XbaI at the extremity of the RHA. The cassette was isolated as a 1500 bp XmaI/XbaI fragment and cloned at the XmaI/XbaI sites of pMTL70621-SacB to yield the KO plasmid pMTL70621-SacB::H16_A0014.

The KO cassette targeting E6A55_RS33050 (equivalent to pHG170, Xiong et al., 2018) was composed of bases 182829 to 183578 (LHA) fused to bases 184512 to 185261 (RHA) of the *C. necator* H16 megaplasmid pHG1 and was designed to delete 933 bp of pHG170. The oligonucleotide primers pairs LHA4_F1/LHA4_SOE_R1 and RHA4_SOE_F1/RHA4_R1 were designed to position XbaI restriction recognition site adjacent to the LHA and at the extremity of the RHA. The cassette was isolated as a 1500 bp XbaI/XbaI fragment and inserted at the XbaI site of pMTL70621-SacB to yield the KO plasmid pMTL70621-SacB::H16_pHG170.

**FIGUIRES**


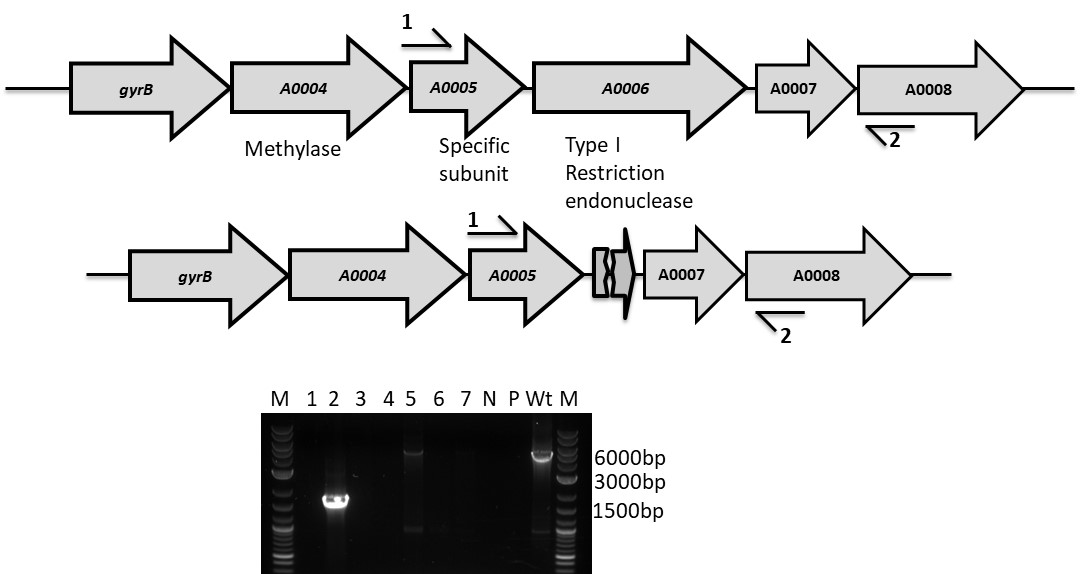


**Figure S1. in-frame deletion of H16_A0006**

Schematic representations of the wild-type (Wt) and putative mutant genomes in the targeted region is shown above the electrophoretogram of the PCR products, together with the relative position of the two primers. Key: M = 2-log DNA marker (NEB) molecular weight marker; N = no DNA control; P = the pMTL70621-SacB::H16_A0006 plasmid only control, and; Wt = wildtype. Lanes 1-7 = the seven SacB^R^ colonies screened using primers H16_A0005_SF1 (1) and A0008_SR1 (2). Expected fragment sizes are 1705 bp in the deletion mutant and 5014 bp fragment in the *C. necator* H16 wildtype, respectively. Accordingly, lane 5 is wildtype, lanes 1, 3, 4, 6, 7 didn’t produce any band and while lane 2 is a pure double crossover mutant.


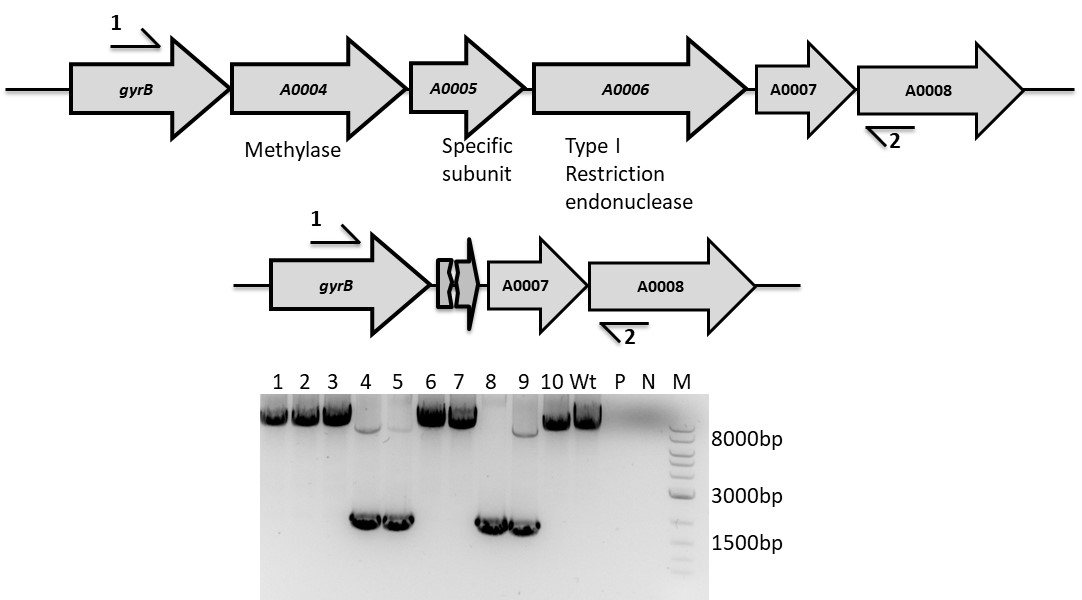


**Figure S2. in-frame deletion of H16_A0004-A0006**

Schematic representations of the wild-type (Wt) and putative mutant genomes in the targeted region is shown above the electrophoretogram of the PCR products, together with the relative position of the two primers. Key: M = 2-log DNA marker (NEB) molecular weight marker; N = no DNA control; P = the pMTL70621-SacB::H16_A0004-A0006 plasmid only control, and; Wt = wildtype. Lanes 1-10 = the ten SacB^R^ colonies screened using primers gyrB_SF1 (1) and A0008_SR1 (2). Expected fragment sizes are 1684 bp in the deletion mutant and 8757 bp fragment in the *C. necator* H16 wildtype, respectively. Accordingly, labelled lanes 1, 2, 3, 6,7 and 10 are wildtype, lanes 4, 5 and 9 are a mixture of wild-type and mutant while lane 8 is a pure double crossover mutant.


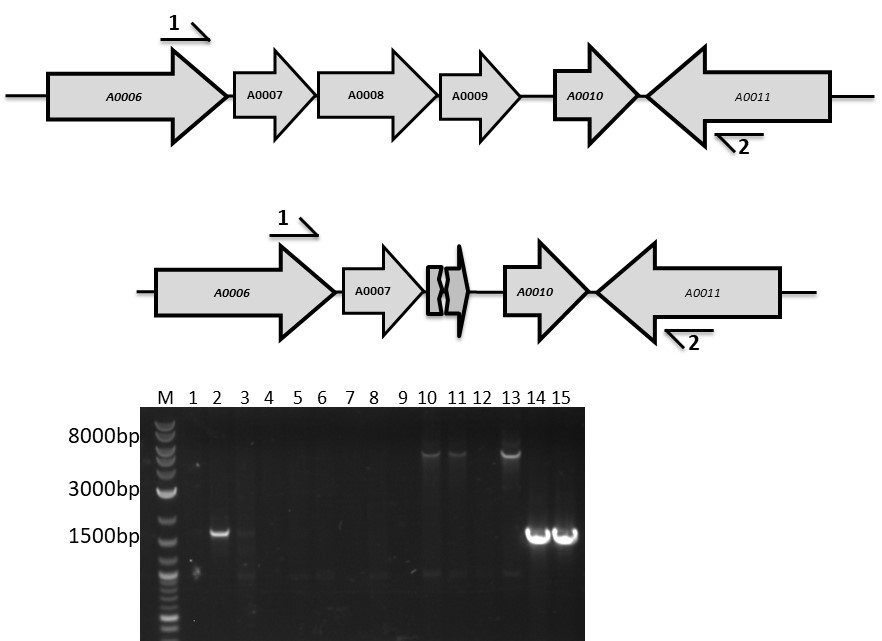


**Figure S3. in-frame deletion of H16_A0008-A0009**

Schematic representations of the wild-type (Wt) and putative mutant genomes in the targeted region is shown above the electrophoretogram of the PCR products, together with the relative position of the two primers. Key: M = 2-log DNA marker (NEB) molecular weight marker, and; Lanes 1-15 = the fifteen SacB^R^ colonies screened using primers H16-A0006-SF1 (1) and H16_A0011-SR1 (2). Expected fragment sizes are 1666 bp in the deletion mutant and 5394 bp fragment in the *C. necator* H16 wildtype, respectively. Accordingly, lanes 10, 11, 13 are wild-type, lanes 2, 3, 14 and 15 are pure double crossover mutants while lanes 1, 4-9 and 12 didn’t produce any band.


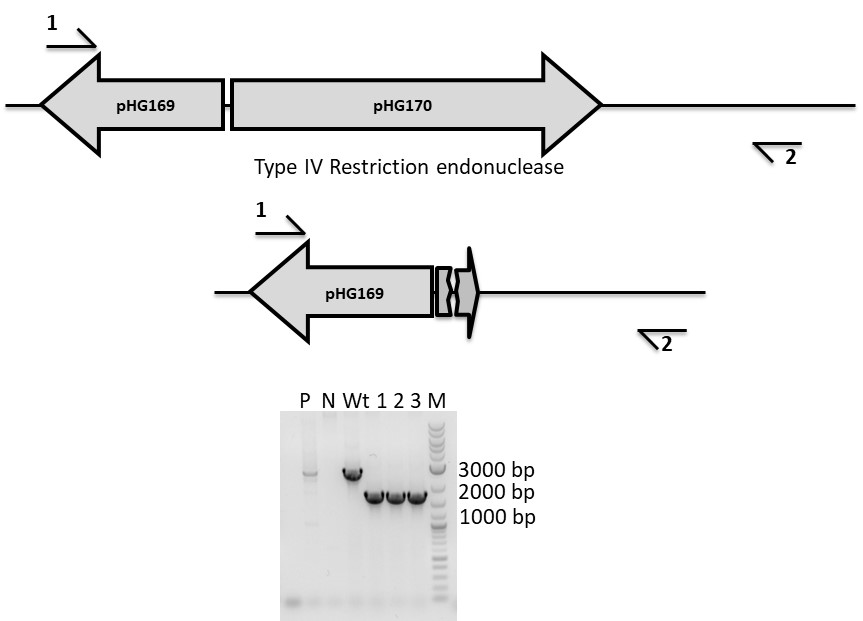


**Figure S4. in-frame deletion of H16_pHG170**

Schematic representations of the wild-type (Wt) and putative mutant genomes in the targeted region is shown above the electrophoretogram of the PCR products, together with the relative position of the two primers. Key: M = 2-log DNA marker (NEB) molecular weight marker; N = no DNA control; P = the pMTL70621-SacB::H16_pHG170 plasmid only control, and; Wt = the wild-type. Lanes 1-3 = the three SacB^R^ colonies screened using primers pHG169_SF1 (1) and pHG170_SR1 (2). Expected fragment sizes are 1731 bp in the deletion mutant and 2664 bp fragment in the *C. necator* H16 wildtype, respectively. Accordingly, labelled lanes 1, 2 and 3 are pure double crossover mutants.


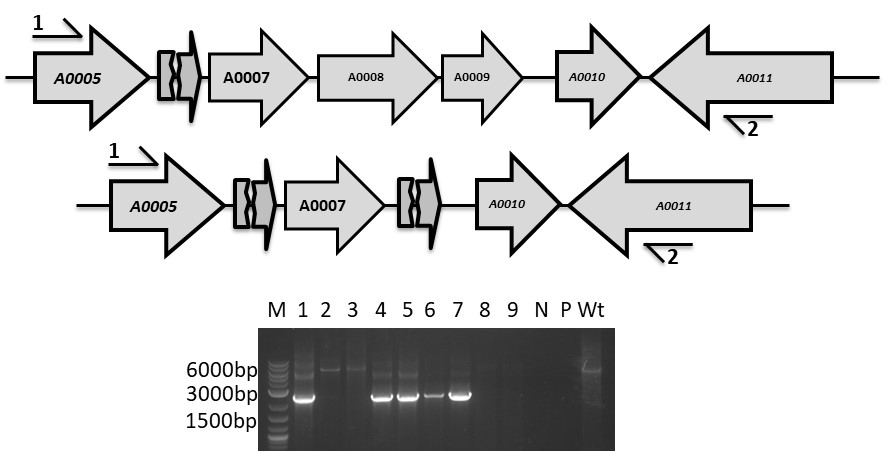


**Figure S5. in-frame deletion of H16_A0008-A0009 in strain H16ΔH16_A0006 to generate H16ΔH16_A0006Δ H16_A0008-A0009**

Schematic representations of the wild-type (Wt) and putative mutant genomes in the targeted region is shown above the electrophoretogram of the PCR products, together with the relative position of the two primers. Key: M = 2-log DNA marker (NEB) molecular weight marker; N = no DNA control; P = the pMTL70621-SacB::H16_A0008-A0009 plasmid only control, and; Wt = the wildtype. Lanes 1-9 = the nine SacB^R^ colonies screened using primers H16_A0005_SF1 (1) and A0011_SR1 (2). Expected fragment sizes are 2425 bp in the deletion mutant and 6153 bp fragment in the *C. necator* H16 wildtype, respectively. Accordingly, lanes 2 and 3 are wildtype, lanes 1, 4, 5, 6, 7 are pure double crossover mutants, while 8 and 9 didn’t produce any band.

**A]**


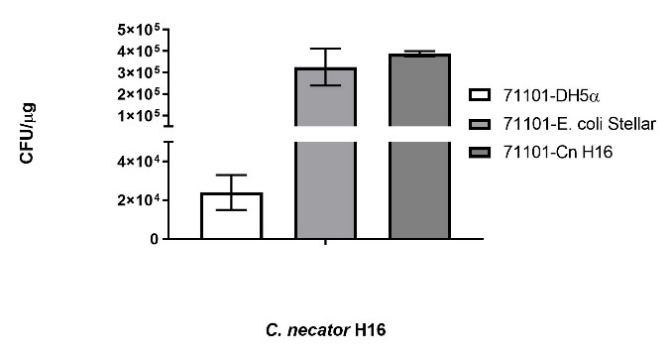


**B]**

**Figure S6. Electroporation efficiencies of different plasmid DNA in different *C. necator* H16 strains**.

**A]** Electroporation efficiencies of pMTL71101 extracted from *E. coli* DH5α, *E. coli* Stellar and *C. necator* H16 in *C. necator* H16, 71101-DH5α extracted from *E. coli* DH5α, 71101-*E. coli* Stellar extracted from *E. coli* Stellar and 71101-H16 extracted from *C. necator* H16

**B]** Electroporation efficiencies of pMTL71101 extracted from *E. coli* Top10 in *C. necator* H16 WT and engineered *C. necator* H16 strains.

**REFERENCES**

Alagesan S, Hanko EKR, Malys N, Ehsaan M, Winzer K, Minton NP. Functional Genetic Elements for Controlling Gene Expression in *Cupriavidus necator* H16. *Appl Environ Microbiol,* 2018; **84**(19). <https://doi.org/10.1128/AEM.00878-18>

Bagdasarian MM, Amann E, Lurz R, Ruckert B, Bagdasarian M. Activity of the hybrid *trp-lac* (*tac*) promoter of *Escherichia coli* in *Pseudomonas putida*. Construction of broad-host-range, controlled-expression vectors. *Gene*, 1983; **26(2-3):** 273-282. <https://doi.org/10.1016/0378-1119(83​)90197-x>

Bi C, Su P, Muller J, Yeh YC, Chhabra SR, Beller HR, Singer SW, Hillson NJ. Development of a broad-host synthetic biology toolbox for *Ralstonia eutropha* and its application to engineering hydrocarbon biofuel production. *Microbial Cell Factories,* 2013; **12:**107. <https://doi.org/10.1186/1475-2859-12-107>

Bilgrau AE, Falgreen S, Petersen A, Kjeldsen MK, Bodker JS, Johnsen HE, Dybkaer K, Bogsted M. Unaccounted uncertainty from qPCR efficiency estimates entails uncontrolled false positive rates. *BMC Bioinformatics,* 2016; **17:**159. <https://doi.org/10.1186/s12859-016-0997-6>

Chambers SP, Prior SE, Barstow DA, Minton NP. The pMTL nic- cloning vectors. I. Improved pUC polylinker regions to facilitate the use of sonicated DNA for nucleotide sequencing. *Gene,* 1988; **68(1):**139-49. <https://doi.org/10.1016/0378-1119(88)90606-3>

Ehsaan, M., W. Kuit, Y. Zhang, S. T. Cartman, J. T. Heap, K. Winzer and N. P. Minton (2016). Mutant generation by allelic exchange and genome resequencing of the biobutanol organism *Clostridium acetobutylicum* ATCC 824. *Biotechnol Biofuels* 2016; **9:**4 <https://doi.org/10.1186/s13068-015-0410-0>

Grant SGN, Jessee J, Bloom FR, Hanahan D. Differential Plasmid Rescue from Transgenic Mouse Dnas into *Escherichia coli* Methylation-Restriction Mutants. *Proc Natl Acad Sci USA,* 1990; **87(12):**4645-4649. <https://doi.org/10.1073/pnas.87.12.4645>

Heap JT, Pennington OJ, Cartman ST, Minton NP (2009). A modular system for *Clostridium* shuttle plasmids. *J Microbiol Methods,* 2009; **78(1):**79-85. <https://doi.org/10.1016/j.mimet.2009.05.004>

Heeb S, Itoh Y, Nishijyo T, Schnider U, Keel C, Wade J, Walsh U, O'Gara F, Haas D. Small, stable shuttle vectors based on the minimal pVS1 replicon for use in gram-negative, plant-associated bacteria. *Mol Plant Microbe Interact,* 2000; **13(2):**232-237. <https://doi.org/10.3389/fpls.2017.02242>

Kovach ME, Elzer PH, Hill DS, Robertson GT, Farris MA, Roop RM 2nd, Peterson KM. Four new derivatives of the broad-host-range cloning vector pBBR1MCS, carrying different antibiotic-resistance cassettes. *Gene,* 1995;**166(1):**175-6. <https://doi.org/10.1016/0378-1119(95)00584-1>

Lenz O, Friedrich B. A novel multicomponent regulatory system mediates H_2_ sensing in *Alcaligenes eutrophus*. *Proc Natl Acad Sci USA*, 1998; **95(21):**12474-12479. <https://doi.org/10.1073/pnas.95.21.12474>

Lefebre MD, Valvano MA. Construction and evaluation of plasmid vectors optimized for constitutive and regulated gene expression in *Burkholderia cepacia* complex isolates. *Appl Environ Microbiol*, 2002; **68(12):**5956-64. <https://doi.org/10.1128/AEM.68.12.5956-5964.2002>

Marx CJ, Lidstrom ME. Development of improved versatile broad-host-range vectors for use in methylotrophs and other Gram-negative bacteria*. Microbiol*, 2001; **147(8):**2065-2075. <https://doi.org/10.1099/00221287-147-8-2065>

# Phillips GJ. New cloning vectors with temperature-sensitive replication. Plasmid, 1999; **41(1):**78-81. <https://doi.org/10.1006/plas.1998.1380>

Rao X, Huang X, Zhou Z, Lin X. An improvement of the 2^(-delta delta CT) method for quantitative real-time polymerase chain reaction data analysis. *Biostat Bioinforma Biomath,* 2013; **3(3):** 71-85. PMID: 25558171; PMCID: PMC4280562.

Rasmussen R. Quantification on the LightCycler. Rapid Cycle Real-Time PCR: *Methods and Applications*. S. Meuer, C. Wittwer and K.-I. Nakagawara. Berlin, Heidelberg, Springer Berlin Heidelberg: 2001; 21-34.

Schlegel HG, Kaltwasser H, Gottschalk G. A submersion method for culture of hydrogen-oxidizing bacteria: growth physiological studies." *Arch Mikrobiol,* 1961; **38:**209-222. PMID: **13747777.**

Simon R, Priefer U, Puhler A. A Broad Host Range Mobilization System for Invivo Genetic-Engineering - Transposon Mutagenesis in Gram-Negative Bacteria." *Bio-Technol,* 1983; **1(9):** 784-791. <https://doi.org/10.1038/nbt1183-784>

Whelan JA, Russell NB, Whelan MA. A method for the absolute quantification of cDNA using real-time PCR." *J Immunol Methods,* 2003; **278(1-2):** 261-269.

<https://doi.org/10.1016/s0022-1759(03)00223-0>

Xiong B, Li ZK, Liu L, Zhao DD, Zhang XL, Bi CH. Genome editing of *Ralstonia eutropha* using an electroporation-based CRISPR-Cas9 technique. *Biotechnol Biofuels,* 2018; **11(1):**172–181. <https://doi.org/10.1186/s13068-018-1170-4>

# **APPENDIX**

[pMTL70000 Modular vectors and their nucleotide sequences in *.gb format may be sourced from [www.plasmidvectors.com](http://www.plasmidvectors.com) ].

For information, the sequences of the various modules are given here, together with the full sequences of selected plasmids.

***C. necator* replicons**

**(1)** pBBR1 [*mob+*]

gtttaaacgtgctacgcctgaataagtgataataagcggatgaatggcagaaattcgaaagcaaattcgacccggtcgtcggttcagggcagggtcgttaaatagccgcttatgtctattgctggtttaccggtttattgactaccggaagcagtgtgaccgtgtgcttctcaaatgcctgaggccagtttgctcaggctctccccgtggaggtaataattgacgatatgatcatttattctgcctcccagagcctgataaaaacggtgaatccgttagcgaggtgccgccggcttccattcaggtcgaggtggcccggctccatgcaccgcgacgcaacgcggggaggcagacaaggtatagggcggcgaggcggctacagccgatagtctggaacagcgcacttacgggttgctgcgcaacccaagtgctaccggcgcggcagcgtgacccgtgtcggcggctccaacggctcgccatcgtccagaaaacacggctcatcgggcatcggcaggcgctgctgcccgcgccgttcccattcctccgtttcggtcaaggctggcaggtctggttccatgcccggaatgccgggctggctgggcggctcctcgccggggccggtcggtagttgctgctcgcccggatacagggtcgggatgcggcgcaggtcgccatgccccaacagcgattcgtcctggtcgtcgtgatcaaccaccacggcggcactgaacaccgacaggcgcaactggtcgcggggctggccccacgccacgcggtcattgaccacgtaggccgacacggtgccggggccgttgagcttcacgacggagatccagcgctcggccaccaagtccttgactgcgtattggaccgtccgcaaagaacgtccgatgagcttggaaagtgtcttctggctgaccaccacggcgttctggtggcccatctgcgccacgaggtgatgcagcagcattgccgccgtgggtttcctcgcaataagcccggcccacgcctcatgcgctttgcgttccgtttgcacccagtgaccgggcttgttcttggcttgaatgccgatttctctggactgcgtggccatgcttatctccatgcggtaggggtgccgcacggttgcggcaccatgcgcaatcagctgcaacttttcggcagcgcgacaacaattatgcgttgcgtaaaagtggcagtcaattacagattttctttaacctacgcaatgagctattgcggggggtgccgcaatgagctgttgcgtaccccccttttttaagttgttgatttttaagtctttcgcatttcgccctatatctagttctttggtgcccaaagaagggcacccctgcggggttcccccacgccttcggcgcggctccccctccggcaaaaagtggcccctccggggcttgttgatcgactgcgcggccttcggccttgcccaaggtggcgctgcccccttggaacccccgcactcgccgccgtgaggctcggggggcaggcgggcgggcttcgccttcgactgcccccactcgcataggcttgggtcgttccaggcgcgtcaaggccaagccgctgcgcggtcgctgcgcgagccttgacccgccttccacttggtgtccaaccggcaagcgaagcgcgcaggccgcaggccggaggcttttccccagagaaaattaaaaaaattgatggggcaaggccgcaggccgcgcagttggagccggtgggtatgtggtcgaaggctgggtagccggtgggcaatccctgtggtcaagctcgtgggcaggcgcagcctgtccatcagcttgtccagcagggttgtccacgggccgagcgaagcgagccagccggtggccgctcgcggccatcgtccacatatccacgggctggcaagggagcgcagcgaccgcgcagggcgaagcccggagagcaagcccgtagggcgccgcagccgccgtaggcggtcacgactttgcgaagcaaagtctagtgagtatactcaagcattgagtggcccgccggaggcaccgccttgcgctgcccccgtcgagccggttggacaccaaaagggaggggcaggcatggcggcatacgcgatcatgcgatgcaagaagctggcgaaaatgggcaacgtggcggccagtctcaagcacgcctaccgcgagcgcgagacgcccaacgctgacgccagcaggacgccagagaacgagcactgggcggccagcagcaccgatgaagcgatgggccgactgcgcgagttgctgccagagaagcggcgcaaggacgctgtgttggcggtcgagtacgtcatgacggccagcccggaatggtggaagtcggccagccaagaacagcaggcggcgttcttcgagaaggcgcacaagtggctggcggacaagtacggggcggatcgcatcgtgacggccagcatccaccgtgacgaaaccagcccgcacatgaccgcgttcgtggtgccgctgacgcaggacggcaggctgtcggccaaggagttcatcggcaacaaagcgcagatgacccgcgaccagaccacgtttgccgccgctgtggccgatctagggctgcaacggggcatcgagggcagcaaggcacgtcacacgcgcattcaggcgttctacgaggccctggagcggccaccagtgggccacgtcaccatcagcccgcaagcggtcgagccacgcgcctatgcaccgcagggattggccgaaaagctgggaatctcaaagcgcgttgagacgccggaagccgtggccgaccggctgacaaaagcggttcggcaggggtatgagcctgccctacaggccgccgcaggagcgcgtgagatgcgcaagaaggccgatcaagcccaagagacggcccgagaccttcgggagcgcctgaagcccgttctggacgccctggggccgttgaatcgggatatgcaggccaaggccgccgcgatcatcaaggccgtgggcgaaaagctgctgacggaacagcgggaagtccagcgccagaaacaggcccagcgccagcaggaacgcgggcgcgcacatttccccgaaaagtgccacctgacgtctaagaaaccattattatcatgacattaacctataaaaataggcgtatcacgaggccctttcgtcttcgaataaatacctgtgacggaagatcacttcgcagaataaataaatcctggtgtccctgttgataccgggaagccctgggccaacttttggcgaaaatgagacgttgatcggcacgtaagaggttccaactttcaccataatgaaataagatcactaccgggcgtattttttgagttatcgagattttcaggagctaaggaagctaaaatggagaaaaaaatcactggatataccaccgttgatatatcccaatggcatcgtaaagaacattttgaggcatttcagtcagttgctcaatgtacctataaccagaccgttcagctggatattacggcctttttagcttttgccattctcaccggattcagtcgtcactcatggtgatttctcacttgataaccttatttttgacgaggggaaattaataggttgtattgatgttggacgagtcggaatcgcagaccgataccaggatcttgccatcctatggaactgcctcggtgagttttctccttcattacagaaacggctttttcaaaaatatggtattgataatcctgatatgaataaattgcagtttcatttgatgctcgatgagtttttctaatcagaattggttaattggttgtaacactggcagagcattacgctgacttgacgggacggcggctttgttgaataaatcgaacttttgctgagttgaaggatcagatcacgcatcttcccgacaacgcagaccgttccgtggcaaagcaaaagttcaaaatcaccaactggtccacctacaacaaagctctcatcaaccgtggctccctcactttctggctggatgatggggcgattcaggcctggtatgagtcagcaacaccttcttcacgaggcagacctcatcagcctgcagg

**(2)** pMOL28 [*oriV28*/*parABC*]

gtttaaactcgccttttactttaacaacgacccgtaagtaccgggggaacagctcgatctctcgggtacaacaggcacaaagttaacttgcgtatacgtctaagggccgctaaccttcacggcaacgcaaccgcggacgtcatttttgccgaaaacggttgcacgatccaccggcggttccggtgaacagcttaaaggtttttgaaccgaatggatatcaagctagcccaccccagcaagacgacgcctgaggatttgaagcagttggcaaatctctccgcggtgatgctgcagaaaattcgggatgagatgctggagccatttcctcggaaggaagccccgctgatcccgtctggccgcctacaagaattgtgtggcatcgacaaaacgcggatgaaccggtccctcaaaaagggggatctccctcagggccagcaatcgcgacccggtgcagtgcgctatttcagcctcagcgaggcaatgcaatggatccgagcggaacttaagcctgtcccgcgaaggggaccaggtaaagtcattgcagttgcgaacttcaagggcggtgtcacgaagaccactatgtccaccctcctctgccagggcttgagtctgcggcgaggtcggaaggtgtgccacgttgatctggatccgcagggaagcgcaaccacgctgtatggcatcaatccacatgccgaggtgtcgtccgaaaacaccattatgccgctcatcgaggcgtatttggcgggcgagtccttcgatatgcgagggcttcctcaggagacttactggcctaacctggatttgattccttcgtctactgagcttttcaacgcggagtttatgcttccggctcgggcgacggcagaggaaggccatattccgttcgagcgcgtgttaagtaacggcctcgattcgttgaaagacgaatatgactacatcatcctcgacacggctcctaccctcagctacctgaccatcaacgcgattttcgctgccgatggcgtcatcgtaccggtggtcccggacaccttggctttcgcgtctatggtccagttctggcaactcttctcggacctagtaacaggcatggaagagcagagcgagggatctaaaaaggagttcgactttctcgatgttctcatgacacgcatggagaaaaagaacgctcctcgcctggtggcagactggattcgcggcgtctatgggtcgcgcgtgctgccgattgagatccctgagacggacctcgcccgtaacagcagcattcaatttcgcacggtctatgacctctcctctagcgaggcgaacaccgagacgatgcgacgcattcgccaaccctgcgatgagtttgtcgactatgtggacgacaaggtcagcgcgctttggcaaggaattgaagaatgagtttgagagaaaagcttgccgcaaaggctgggaacatcaaggtcacggcggaagacttggagaaagccgctgcgcgcggtccgcaagcgccgcgaactgcgcccggtcagttaatgcatatgcaagggaaggttgagcgacaggctaacgagatcgcgcaactaagagcagaacttgagtcggcccgcgtcagcggcggcgcagtggatgtgcctatcgaccaactgcatgaggtcccaggccgcagacgcttcatgcctcccgagaagtatgtcgaattgagggaaaacctcaggcacaacaagctcgttcatcctgtgattgtatgccctcggcctgcgggaggcttcgagattgtctccgggcatcaccggacagacgcgtaccgcgagcttgggcgcgatcacatacgctgcgtgctcggcgaacttagttcagacgaggctgacacgggcgcgttctacgcgaaccttatgcagtcagatttaacggatttcgagaagtttcggaagttcgacgaactgctgcttcgcagcccagacaagactcaagccgcaatagctgaacaggctggtgtacctgtctcgactctctcagagattttgtcgttccggaacttgcctcccgaggtcctaagccttctcgatagccgcccagacctgctcgggtcgaatgctggcgccgagttggcaagggcgaccaaagacggtcgcggggatcgggtcgtcgaagcggttaagttgttggccgagaagaagatcgatcaacagcaggccgtacggatgactaaggccgagcaggttaagaccaggcctgccgcatctaccggcttcaaaatcaaggcgggaaaggcgacttggtgcgatgttcgtatcgcaaagaaagtcatgcgcattgagttccgcagcgaggaagaagcggaagcggcccaatcggccattcgcgaacatctggaagggttagctaaagctgcgtcggaagacgcaaaaagctaagtgcttgttttttaaggacttcgtactacgaatcgaggttttaagccatgtctagactgtaatcctacaaaaacaaaagcccacggcggcaaccgtgggcttttgagaacttcaagctgaccagtttcccggccgctacacaccgaagccactcgacatggattcgttagtcggagtgtagcggaacgcgaacctgagtcaagcgacttcaaccattttttacgaatgggaaggtcatatgactttcgcgcaacgctccgttgctggcgaggatgttgctcgcccacaaaaacaccttaaccagacagacgctctgattgctccggcgcccaagcgcctcaaacgcaagactatcgaagcagtcgagcgcgcaactcgaatcgtcgggatcgggcgtagcgcccgatcagctcttgccgccctcgcccgcacggcgaataacgatgaccccaccggtaagatctttaagcaccgggaaacgctttgtgccgaaaccggaatgtcgccggctacttggtaccgtgctcaacgagaactgctcgacttgggcctaattaccgtcgacgttcaagttcggaagcgatttggccgattcgcaggagcctacatttacctgacggaaaaagcgacggagatgctcggcttaagctcgcgaaaagaagaagaaaccacgggtacgggcgaggacgacacagcgcagctcggcgagccggccgttccaccaccctcttctatggcgcaaccgtctctcaaaacgagagtcctgtttacagaagatcgtgtcccatactcctttcaaaaaagacagcaggatcggctcccccaggacctgacacgtctgcgcggcctgggtcttgatgtaaatttaattttttggttgatgcgaaaggctaaagagcaaggccactttctctcagatgtcgtaagcgcgacatgggagagtcttgcgaaagcacgcgtgccaaaagcgtatctgcttgccctactcaccgcccgcaccgatttcagtgctgtctgcaaagcaaaggcactcaaagaagacaaagcccgaatccaagtgcaggaccgcgatttcgtgcgttcgatactcgcaggggcagcgcggcagtgtttcgtggacgaaaaaggcaaccatttcgaagtcgaaagcgacggaagctcagtgcttgtcaccgaggtccaaagtgcggtcacttcccgcttggtaggaacttccctcgccgaatttgcaaggcgactacacgctggtgcgtaccagaaagctgaggtctacgctgctccccagagagcaagcggccggctcgagaaacggggaaaggaggcggcttcgacgttatcggcgttgcgagcgatgctgcgcgaccgcaggtcagccaacgcggcaaacacgacgaacaatgctcatgccatggcctagggtgtgttttgcgctgaaagcctgcagg

**(3)** RSF1010 (pKT240)

gtttaaacttcttcaaattcccgttgcacatagcccggcaattcctttccctgctctgccataagcgcagcgaatgccgggtaatactcgtcaacgatctgatagagaagggtttgctcgggtcggtggctctggtaacgaccagtatcccgatcccggctggccgtcctggccgccacatgaggcatgttccgcgtccttgcaatactgtgtttacatacagcctatcgcttagcggaaagttcttttaccctcagccgaaatgcctgccgttgctagacattgccagccagtgcccgtcactcccgtactaactgtcacgaacccctgcaataactgtcacgcccccctgcaataactgtcacgaacccctgcaataactgtcacgcccccaaacctgcaaacccagcaggggcgggggctggcggggtgttggaaaaatccatccatgattatctaagaataatccactaggcgcggttatcagcgcccttgtggggcgctgctgcccttgcccaatatgcccggccagaggccggatagctggtctattcgctgcgctaggctacacaccgccccaccgctgcgcggcagggggaaaggcgggcaaagcccgctaaaccccacaccaaaccccgcagaaatacgctggagcgcttttagccgctttagcggcctttccccctacccgaagggtgggggcgcgtgtgcagccccgcagggcctgtctcggtcgatcattcagcccggctcatccttctggcgtggcggcagaccgaacaaggcgcggtcgtggtcgcgttcaaggtacgcatccattgccgccatgagccgatcctccggccactcgctgctgttcaccttggccaaaatcatggcccccaccagcaccttgcgccttgtttcgttcttgcgctcttgctgctgttcccttgcccgcacccgctgaatttcggcattgattcgcgctcgttgttcttcgagcttggccagccgatccgccgccttgttgctccccttaaccatcttgacaccccattgttaatgtgctgtctcgtaggctatcatggaggcacagcggcggcaatcccgaccctactttgtaggggagggcgcacttaccggtttctcttcgagaaactggcctaacggccacccttcgggcggtgcgctctccgagggccattgcatggagccgaaaagcaaaagcaacagcgaggcagcatggcgatttatcaccttacggcgaaaaccggcagcaggtcgggcggccaatcggccagggccaaggccgactacatccagcgcgaaggcaagtatgcccgcgacatggatgaagtcttgcacgccgaatccgggcacatgccggagttcgtcgagcggcccgccgactactgggatgctgccgacctgtatgaacgcgccaatgggcggctgttcaaggaggtcgaatttgccctgccggtcgagctgaccctcgaccagcagaaggcgctggcgtccgagttcgcccagcacctgaccggtgccgagcgcctgccgtatacgctggccatccatgccggtggcggcgagaacccgcactgccacctgatgatctccgagcggatcaatgacggcatcgagcggcccgccgctcagtggttcaagcggtacaacggcaagaccccggagaagggcggggcacagaagaccgaagcgctcaagcccaaggcatggcttgagcagacccgcgaggcatgggccgaccatgccaaccgggcattagagcgggctggccacgacgcccgcattgaccacagaacacttgaggcgcagggcatcgagcgcctgcccggtgttcacctggggccgaacgtggtggagatggaaggccggggcatccgcaccgaccgggcagacgtggccctgaacatcgacaccgccaacgcccagatcatcgacttacaggaataccgggaggcaatagaccatgaacgcaatcgacagagtgaagaaatccagaggcatcaacgagttagcggagcagatcgaaccgctggcccagagcatggcgacactggccgacgaagcccggcaggtcatgagccagacccagcaggccagcgaggcgcaggcggcggagtggctgaaagcccagcgccagacaggggcggcatgggtggagctggccaaagagttgcgggaggtagccgccgaggtgagcagcgccgcgcagagcgcccggagcgcgtcgcgggggtggcactggaagctatggctaaccgtgatgctggcttccatgatgcctacggtggtgctgctgatcgcatcgttgctcttgctcgacctgacgccactgacaaccgaggacggctcgatctggctgcgcttggtggcccgatgaagaacgacaggactttgcaggccataggccgacagctcaaggccatgggctgtgagcgcttcgatatcggcgtcagggacgccaccaccggccagatgatgaaccgggaatggtcagccgccgaagtgctccagaacacgccatggctcaagcggatgaatgcccagggcaatgacgtgtatatcaggcccgccgagcaggagcggcatggtctggtgctggtggacgacctcagcgagtttgacctggatgacatgaaagccgagggccgggagcctgccctggtagtggaaaccagcccgaagaactatcaggcatgggtcaaggtggccgacgccgcaggcggtgaacttcgggggcagattgcccggacgctggccagcgagtacgacgccgacccggccagcgccgacagccgccactatggccgcttggcgggcttcaccaaccgcaaggacaagcacaccacccgcgccggttatcagccgtgggtgctgctgcgtgaatccaagggcaagaccgccaccgctggcccggcgctggtgcagcaggctggccagcagatcgagcaggcccagcggcagcaggagaaggcccgcaggctggccagcctcgaactgcccgagcggcagcttagccgccaccggcgcacggcgctggacgagtaccgcagcgagatggccgggctggtcaagcgcttcggtgatgacctcagcaagtgcgactttatcgccgcgcagaagctggccagccggggccgcagtgccgaggaaatcggcaaggccatggccgaggccagcccagcgctggcagagcgcaagcccggccacgaagcggattacatcgagcgcaccgtcagcaaggtcatgggtctgcccagcgtccagcttgcgcgggccgagctggcacgggcaccggcaccccgccagcgaggcatggacaggggcgggccagatttcagcatgtagtgcttgcgttggtactcacgcctgttatactatgagtactcacgcacagaagggggttttatggaatacgaaaaaagcgcttcagggtcggtctacctgatcaaaagtgacaagggctattggttgcccggtggctttggttatacgtcaaacaaggccgaggctggccgcttttcagtcgctgatatggccagccttaaccttgacggctgcaccttgtccttgttccgcgaagacaagcctttcggccccggcaagtttctcggtgactgatatgaaagaccaaaaggacaagcagaccggcgacctgctggccagccctgacgctgtacgccaagcgcgatatgccgagcgcatgaaggccaaagggatgcgtcagcgcaagttctggctgaccgacgacgaatacgaggcgctgcgcgagtgcctggaagaactcagagcggcgcagggcgggggtagtgaccccgccagcgcctaaccaccaactgcctgcaaaggaggcaatcaatggctacccataagcctatcaatattctggaggcgttcgcagcagcgccgccaccgctggactacgttttgcccaacatggtggccggtacggtcggggcgctggtgtcgcccggtggtgccggtaaatccatgctggccctgcaactggccgcacagattgcaggcgggccggatctgctggaggtgggcgaactgcccaccggcccggtgatctacctgcccgccgaagacccgcccaccgccattcatcaccgcctgcacgcccttggggcgcacctcagcgccgaggaacggcaagccgtggctgacggcctgctgatccagccgctgatcggcagcctgcccaacatcatggccccggagtggttcgacggcctcaagcgcgccgccgagggccgccgcctgatggtgctggacacgctgcgccggttccacatcgaggaagaaaacgccagcggccccatggcccaggtcatcggtcgcatggaggccatcgccgccgataccgggtgctctatcgtgttcctgcaccatgccagcaagggcgcggccatgatgggcgcaggcgaccagcagcaggccagccggggcagctcggtactggtcgataacatccgctggcagtcctacctgtcgagcatgaccagcgccgaggccgaggaatggggtgtggacgacgaccagcgccggttcttcgtccgcttcggtgtgagcaaggccaactatggcgcaccgttcgctgatcggtggttcaggcggcatgacggcggggtgctcaagcccgccgtgctggagaggcagcgcaagagcaagggggtgccccgtggtgaagcctaagaacaagcacagcctcagccacgtccggcacgacccggcgcactgtctggcccccggcctgttccgtgccctcaagcggggcgagcgcaagcgcagcaagctggacgtgacgtatgactacggcgacggcaagcggatcgagttcagcggcccggagccgctgggcgctgatgatctgcgcatcctgcaagggctggtggccatggctgggcctaatggcctagtgcttggcccggaacccaagaccgaaggcggacggcagctccggctgttcctggaacccaagtgggaggccgtcaccgctgatgccatggtggtcaaaggtagctatcgggcgctggcaaaggaaatcggggcagaggtcgatagtggtggggcgctcaagcacatacaggactgcatcgagcgcctttggaaggtatccatcatcgcccagaatggccgcaagcggcaggggtttcggctgctgtcggagtacgccagcgacgaggcggacgggcgcctgtacgtggccctgaaccccttgatcgcgcaggccgtcatgggtggcggccagcatgtgcgcatcagcatggacgaggtgcgggcgctggacagcgaaaccgcccgcctgctgcaccagcggctgtgtggctggatcgaccccggcaaaaccggcaaggcttccatagataccttgtgcggctatgtctggccgtcagaggccagtggttcgaccatgcgcaagcgccgccagcgggtgcgcgaggcgttgccggagctggtcgcgctgggctggacggtaaccgagttcgcggcgggcaagtacgacatcacccggcccaaggcggcaggctgaccccccccactctattgtaaacaagacatttttatcttttatattcaatggcttattttcctgctaattggtaataccatgaaaaataccatgctcagaaaaggcttaacaatattttgaaaaattgcctactgagcgctgccgcacagctccataggccgctttcctggctttgcttccagatgtatgctcttctgctcctgcagg

**(4)** RK2 [+*traJ*] (pCM27)

gtttaaaccgatactgagcgaagcaagtgcgtcgagcagtgcccgcttgttcctgaaatgccagtaaagcgctggctgctgaacccccagccggaactgaccccacaaggccctagcgtttgcaatgcaccaggtcatcattgacccaggcgtgttccaccaggccgctgcctcgcaactcttcgcaggcttcgccgacctgctcgcgccacttcttcacgcgggtggaatccgatccgcacatgaggcggaaggtttccagcttgagcgggtacggctcccggtgcgagctgaaatagtcgaacatccgtcgggccgtcggcgacagcttgcggtacttctcccatatgaatttcgtgtagtggtcgccagcaaacagcacgacgatttcctcgtcgatcaggacctggcaacgggacgttttcttgccacggtccaggacgcggaagcagtgcagcagcgacaccgattccaggtgcccaacgcggtcggacgtgaagcccatcgccgtcgcctgtaggcgcgacaggcattcctcggccttcgtgtaataccggccattgatcgaccagcccaggtcctggcaaagctcgtagaacgtgaaggtgatcggctcgccgataggggtgcgcttcgcgtactccaacacctgctgccacaccagttcgtcatcgtcggcccgcagctcgacgccggtgtaggtgatcttcacgtccttgttgacgtggaaaatgaccttgttttgcagcgcctcgcgcgggattttcttgttgcgcgtggtgaacagggcagagcgggccgtgtcgtttggcatcgctcgcatcgtgtccggccacggcgcaatatcgaacaaggaaagctgcatttccttgatctgctgcttcgtgtgtttcagcaacgcggcctgcttggcctcgctgacctgttttgccaggtcctcgccggcggtttttcgcttcttggtcgtcatagttcctcgcgtgtcgatggtcatcgacttcgccaaacctgccgcctcctgttcgagacgacgcgaacgctccacggcggccgatggcgcgggcagggcagggggagccagttgcacgctgtcgcgctcgatcttggccgtagcttgctggaccatcgagccgacggactggaaggtttcgcggggcgcacgcatgacggtgcggcttgcgatggtttcggcatcctcggcggaaaaccccgcgtcgatcagttcttgcctgtatgccttccggtcaaacgtccgattcattcaccctccttgcgggattgccccgactcacgccggggcaatgtgcccttattcctgatttgacccgcctggtgccttggtgtccagataatccaccttatcggcaatgaagtcggtcccgtagaccgtctggccgtccttctcgtacttggtattccgaatcttgccctgcacgaataccagctccgcgaagtcgctcttcttgatggagcgcatggggacgtgcttggcaatcacgcgcaccccccggccgttttagcggctaaaaaagtcatggctctgccctcgggcggaccacgcccatcatgaccttgccaagctcgtcctgcttctcttcgatcttcgccagcagggcgaggatcgtggcatcaccgaaccgcgccgtgcgcgggtcgtcggtgagccagagtttcagcaggccgcccaggcggcccaggtcgccattgatgcgggccagctcgcggacgtgctcatagtccacgacgcccgtgattttgtagccctggccgacggccagcaggtaggcctacaggctcatgccggccgccgccgccttttcctcaatcgctcttcgttcgtctggaaggcagtacaccttgataggtgggctgcccttcctggttggcttggtttcatcagccatccgcttgccctcatctgttacgccggcggtagccggccagcctcgcagagcaggattcccgttgagcaccgccaggtgcgaataagggacagtgaagaaggaacacccgctcgcgggtgggcctacttcacctatcctgcccggctgacgccgttggatacaccaaggaaagtctacacgaaccctttggcaaaatcctgtatatcgtgcgaaaaaggatggatataccgaaaaaatcgctataatgaccccgaagcagggttatgcagcggaaaagatccgtcgaccctttccgacgctcaccgggctggttgccctcgccgctgggctggcggccgtctatggccctgcaaacgcgccagaaacgccgtcgaagccgtgtgcgagacaccgcggccgccggcgttgtggatacctcgcggaaaacttggccctcactgacagatgaggggcggacgttgacacttgaggggccgactcacccggcgcggcgttgacagatgaggggcaggctcgatttcggccggcgacgtggagctggccagcctcgcaaatcggcgaaaacgcctgattttacgcgagtttcccacagatgatgtggacaagcctggggataagtgccctgcggtattgacacttgaggggcgcgactactgacagatgaggggcgcgatccttgacacttgaggggcagagtgctgacagatgaggggcgcacctattgacatttgaggggctgtccacaggcagaaaatccagcatttgcaagggtttccgcccgtttttcggccaccgctaacctgtcttttaacctgcttttaaaccaatatttataaaccttgtttttaaccagggctgcgccctgtgcgcgtgaccgcgcacgccgaaggggggtgcccccccttctcgaaccctcccggcccgctaacgcgggcctcccatccccccaggggctgcgcccctcggccgcgaacggcctcaccccaaaaatggcagccaagctgaccacttctgcctgcagg

**(5)** pVS1 (pME6031)

gtttaaacatgaaaccgcaccaggacggccaggacgaaccgtttttcattaccgaagagatcgaggcggagatgatcgcggccgggtacgtgttcgagccgcccgcgcacgtctcaaccgtgcggctgcatgaaatcctggccggtttgtctgatgccaagctggcggcctggccggccagcttggccgctgaagaaaccgagcgccgccgtctaaaaaggtgatgtgtatttgagtaaaacagcttgcgtcatgcggtcgctgcgtatatgatgcgatgagtaaataaacaaatacgcaaggggaacgcatgaaggttatcgctgtacttaaccagaaaggcgggtcaggcaagacgaccatcgcaacccatctagcccgcgccctgcaactcgccggggccgatgttctgttagtcgattccgatccccagggcagtgcccgcgattgggcggccgtgcgggaagatcaaccgctaaccgttgtcggcatcgaccgcccgacgattgaccgcgacgtgaaggccatcggccggcgcgacttcgtagtgatcgacggagcgccccaggcggcggacttggctgtgtccgcgatcaaggcagccgacttcgtgctgattccggtgcagccaagcccttacgacatatgggccaccgccgacctggtggagctggttaagcagcgcattgaggtcacggatggaaggctacaagcggcctttgtcgtgtcgcgggcgatcaaaggcacgcgcatcggcggtgaggttgccgaggcgctggccgggtacgagctgcccattcttgagtcccgtatcacgcagcgcgtgagctacccaggcactgccgccgccggcacaaccgttcttgaatcagaacccgagggcgacgctgcccgcgaggtccaggcgctggccgctgaaattaaatcaaaactcatttgagttaatgaggtaaagaggaaatgagcaaaagcacaaacacgctaagtgccggccgtccgagcgcacgcagcagcaaggctgcaacgttggccagcctggcagacacgccagccatgaagcgggtcaactttcagttgtcggcggaggatcacaccaagctgaagatgtacgcggtacgccaaggcaagaccattaccgagctgctatctgaatacatcgcgcagctaccagagtaaatgagcaaatgaataatggagtagatgaattttagcggctaaaggaggcggcatggaaaatcaagaacaaccaggcaccgacgccgtggaatgccccatgtgtggaggaacgggcggttggccaggcgtaagcggctgggttgtctgccggccctgcaatggcactggaacccccaagcccgaggaatcggcgtgagcggtcgcaaaccatccggcccggtacaaatcggcgcggcgctgggtgatgacctggtggagaagttgaaggccgcgcaggccgcccagcggcaacgcatcgaggcagaagcacgccccggtgaatcgtggcaagcggccgctgatcgaatccgcaaagaatcccggcaaccgccggcagccggtgcgccgtcgattaggaagccgcccaagggcgacgagcaaccagattttttcgttccgatgctctatgacgtgggcacccgcgatagtcgcagcatcatggacgtggccgttttccgtctgtcgaagcgtgaccgacgagctggcgaggtgatccgctacgagcttccagacgggcacgtagaggtttccgcagggccggccggcatggccagtgtgtgggattacgacctggtactgatggcggtttcccatctaaccgaatccatgaaccgataccgggaagggaagggagacaagcccggccgcgtgttccgtccacacgttgcggacgtactcaagttctgccggcgagccgatggcggaaagcagaaagacgacctggtagaaacctgcattcggttaaacaccacgcacgttgccatgcagcgtacgaagaaggccaagaacggccgcctggtgacggtatccgagggtgaagccttgattagccgctacaagatcgtaaagagcgaaaccgggcggccggagtacatcgagatcgagctagctgattggatgtaccgcgagatcacagaaggcaagaacccggacgtgctgacggttcaccccgattactttttgatcgatcccggcatcggccgttttctctaccgcctggcacgccgcgccgcaggcaaggcagaagccagatggttgttcaagacgatctacgaacgcagtggcagcgccggagagttcaagaagttctgtttcaccgtgcgcaagctgatcgggtcaaatgacctgccggagtacgatttgaaggaggaggcggggcaggctggcccgatcctagtcatgcgctaccgcaacctgatcgagggcgaagcatccgccggttcctaatgtacggagcagatgctagggcaaattgccctagcaggggaaaaaggtcgaaaaggtctctttcctgtggatagcacgtacattgggaacccaaagccgtacattgggaaccggaacccgtacattgggaacccaaagccgtacattgggaaccggccacacatgtaagtgactgatataaaagagaaaaaaggcgatttttccgcctaaaactctttaaaacttattaaaactcttaaaacccgcctggcctgtgcataactgtctggccagcgcacagccgaagagctgcaaaaagcgcctacccttcggtcgctgcgctccctacgccccgccgcttcgcgtcggcctatcgcggccgctggccgctcaaaaatggctggcctacggccaggcaatctaccagggcgcggacaagccgcgccgtcgccactcgaccgccggcgctcctgcagg

**Selectable marker modules**

**(1)** *catP* (pMTL85141)

ggccggccagtgggcaagttgaaaaattcacaaaaatgtggtataatatctttgttcattagagcgataaacttgaatttgagagggaacttagatggtatttgaaaaaattgataaaaatagttggaacagaaaagagtattttgaccactactttgcaagtgtaccttgtacctacagcatgaccgttaaagtggatatcacacaaataaaggaaaagggaatgaaactatatcctgcaatgctttattatattgcaatgattgtaaaccgccattcagagtttaggacggcaatcaatcaagatggtgaattggggatatatgatgagatgataccaagctatacaatatttcacaatgatactgaaacattttccagcctttggactgagtgtaagtctgactttaaatcatttttagcagattatgaaagtgatacgcaacggtatggaaacaatcatagaatggaaggaaagccaaatgctccggaaaacatttttaatgtatctatgataccgtggtcaaccttcgatggctttaatctgaatttgcagaaaggatatgattatttgattcctatttttactatggggaaatattataaagaagataacaaaattatacttcctttggcaattcaagttcatcacgcagtatgtgacggatttcacatttgccgttttgtaaacgaattgcaggaattgataaatagttaacttcaggtttgtctgtaactaaaaacaagtatttaagcaaaaacatcgtagaaatacggtgttttttgttaccctaagtttaaac

**(2)** *amp* (pBR322)

ggccggccaggtggcacttttcggggaaatgtgcgcggaacccctatttgtttatttttctaaatacattcaaatatgtatccgctcatgagacaataaccctgataaatgcttcaataatattgaaaaaggaagagtatgagtattcaacatttccgtgtcgcccttattcccttttttgcggcattttgccttcctgtttttgctcacccagaaacgctggtgaaagtaaaagatgctgaagatcagttgggtgcacgagtgggttacatcgaactggatctcaacagcggtaagatccttgagagttttcgccccgaagaacgttttccaatgatgagcacttttaaagttctgctatgtggcgcggtattatcccgtattgacgccgggcaagagcaactcggtcgccgcatacactattctcagaatgacttggttgagtactcaccagtcacagaaaagcatcttacggatggcatgacagtaagagaattatgcagtgctgccataaccatgagtgataacactgcggccaacttacttctgacaacgatcggaggaccgaaggagctaaccgcttttttgcacaacatgggggatcatgtaactcgccttgatcgttgggaaccggagctgaatgaagccataccaaacgacgagcgtgacaccacgatgcctgtagcaatggcaacaacgttgcgcaaactattaactggcgaactacttactctagcttcccggcaacaattaatagactggatggaggcggataaagttgcaggaccacttctgcgctcggcccttccggctggctggtttattgctgataaatctggagccggtgagcgtgggtctcgcggtatcattgcagcactggggccagatggtaagccctcccgtatcgtagttatctacacgacggggagtcaggcaactatggatgaacgaaatagacagatcgctgagataggtgcctcactgattaagcattggtaactgtcagaccaagtttactcatatatactttagattgatttaaaacttcatttttaatttaaaaggatctaggtgaagatcctttttgataatctcatgaccaaaatcccttaacgtgagttttcgttccactgagcgtcagagtttaaac

**(3)** *tetA/R* (pBBR1)

ggccggccgcaggagcgccagaaggccgccagagaggccgagcgcggccgtgaggcttggacgctagggcagggcatgaaaaagcccgtagcgggctgctacgggcgtctgacgcggtggaaagggggaggggatgttgtctacatggctctgctgtagtgagtgggttgcgctccggcagcggtcctgatcaatcgtcaccctttctcggtccttcaacgttcctgacaacgagcctccttttcgccaatccatcgacaatcaccgcgagtccctgctcgaacgctgcgtccggaccggcttcgtcgaaggcgtctatcgcggcccgcaacagcggcgagagcggagcctgttcaacggtgccgccgcgctcgccggcatcgctgtcgccggcctgctcctcaagcacggccccaacagtgaagtagctgattgtcatcagcgcattgacggcgtccccggccgaaaaacccgcctcgcagaggaagcgaagctgcgcgtcggccgtttccatctgcggtgcgcccggtcgcgtgccggcatggatgcgcgcgccatcgcggtaggcgagcagcgcctgcctgaagctgcgggcattcccgatcagaaatgagcgccagtcgtcgtcggctctcggcaccgaatgcgtatgattctccgccagcatggcttcggccagtgcgtcgagcagcgcccgcttgttcctgaagtgccagtaaagcgccggctgctgaacccccaaccgttccgccagtttgcgtgtcgtcagaccgtctacgccgacctcgttcaacaggtccagggcggcacggatcactgtattcggctgcaactttgtcatgcttgacactttatcactgataaacataatatgtccaccaacttatcagtgataaagaatccgcgcgttcaatcggaccagcggaggctggtccggaggccagacgtgaaacccaacatacccctgatcgtaattctgagcactgtcgcgctcgacgctgtcggcatcggcctgattatgccggtgctgccgggcctcctgcgcgatctggttcactcgaacgacgtcaccgcccactatggcattctgctggcgctgtatgcgttggtgcaatttgcctgcgcacctgtgctgggcgcgctgtcggatcgtttcgggcggcggccaatcttgctcgtctcgctggccggcgccactgtcgactacgccatcatggcgacagcgcctttcctttgggttctctatatcgggcggatcgtggccggcatcaccggggcgactggggcggtagccggcgcttatattgccgatatcactgatggcgatgagcgcgcgcggcacttcggcttcatgagcgcctgtttcgggttcgggatggtcgcgggacctgtgctcggtgggctgatgggcggtttctccccccacgctccgttcttcgccgcggcagccttgaacggcctcaatttcctgacgggctgtttccttttgccggagtcgcacaaaggcgaacgccggccgttacgccgggaggctctcaacccgctcgcttcgttccggtgggcccggggcatgaccgtcgtcgccgccctgatggcggtcttcttcatcatgcaacttgtcggacaggtgccggccgcgctttgggtcattttcggcgaggatcgctttcactgggacgcgaccacgatcggcatttcgcttgccgcatttggcattctgcattcactcgcccaggcagtgatcaccggccctgtagccgcccggctcggcgaaaggcgggcactcatgctcggaatgattgccgacggcacaggctacatcctgcttgccttcgcgacacggggatggatggcgttcccgatcatggtcctgcttgcttcgggtggcatcggaatgccggcgctgcaagcaatgttgtccaggcaggtggatgaggaacgtcaggggcagctgcaaggctcactggcggcgctcaccagcctgacctcgatcgtcggacccctcctcttcacggcgatctatgcggcttctataacaacgtggaacgggtgggcatggattgcgggcgctgccctctacttgctctgcctgccggcgctgcgtcgcgggctttggagcggcgcagggcaacgagccgatcgctgatcgtggaaacgataggaaacgtggccaatatggacaacttcttcgcccccgttttcaccatgggcaaatattacacgcaaggcgacaaggtgctgatgccgctggcgattcaggttcatcatgccgtttgtgatggcttccatgtcggcagaatgcttaatgaattacaacagtttttatgcatgcgcccaatacgcaaaccgcctctccccgcgcgttggccgattcattaatgcagctggcacgacaggtttcccgactggaaagcgggcagtgaacgcaacgcaattaatgtgagttagctcactcattaggcaccccaggctttacactttatgcttccggctcgtatgttgtgtggaattgtgagcggtttaaac

**(4)** *kan* (pBBR1MSC-2)

ggccggccggatgaatgtcagctactgggctatctggacaagggaaaacgcaagcgcaaagagaaagcaggtagcttgcagtgggcttacatggcgatagctagactgggcggttttatggacagcaagcgaaccggaattgccagctggggcgccctctggtaaggttgggaagccctgcaaagtaaactggatggctttcttgccgccaaggatctgatggcgcaggggatcaagatctgatcaagagacaggatgaggatcgtttcgcatgattgaacaagatggattgcacgcaggttctccggccgcttgggtggagaggctattcggctatgactgggcacaacagacaatcggctgctctgatgccgccgtgttccggctgtcagcgcaggggcgcccggttctttttgtcaagaccgacctgtccggtgccctgaatgaactgcaggacgaggcagcgcggctatcgtggctggccacgacgggcgttccttgcgcagctgtgctcgacgttgtcactgaagcgggaagggactggctgctattgggcgaagtgccggggcaggatctcctgtcatctcaccttgctcctgccgagaaagtatccatcatggctgatgcaatgcggcggctgcatacgcttgatccggctacctgcccattcgaccaccaagcgaaacatcgcatcgagcgagcacgtactcggatggaagccggtcttgtcgatcaggatgatctggacgaagagcatcaggggctcgcgccagccgaactgttcgccaggctcaaggcgcgcatgcccgacggcgaggatctcgtcgtgacccatggcgatgcctgcttgccgaatatcatggtggaaaatggccgcttttctggattcatcgactgtggccggctgggtgtggcggaccgctatcaggacatagcgttggctacccgtgatattgctgaagagcttggcggcgaatgggctgaccgcttcctcgtgctttacggtatcgccgctcccgattcgcagcgcatcgccttctatcgccttcttgacgagttcttctgagtttaaac

**(5)** *dhfR* (*P. aeruginosa*)

ggccggcctgacagatgagacaataaccctgataaatgcttcaataatattgaaaaaggaggaagagtatggaacgaagtagcaatgaagtcagtaatccagttgctggcaattttgtattcccatcgaacgccacgtttggtatgggagatcgcgtgcgcaagaaatccggcgccgcctggcaaggtcagattgtcgggtggtactgcacaaatttgacccccgaaggctacgccgtcgagtctgaggctcacccaggctcagtacagatttatcctgttgcggcgcttgaacgcatcaactgagtttaaac

**(6)** *tet* (pBR322)

ggccggcctaattctcatgtttgacagcttatcatcgataagctttaatgcggtagtttatcacagttaaattgctaacgcagtcaggcaccgtgtatgaaatctaacaatgcgctcatcgtcatcctcggcaccgtcaccctggatgctgtaggcataggcttggttatgccggtactgccgggcctcttgcgggatatcgtccattccgacagcatcgccagtcactatggcgtgctgctagcgctatatgcgttgatgcaatttctatgcgcacccgttctcggagcactgtccgaccgctttggccgccgcccagtcctgctcgcttcgctacttggagccactatcgactacgcgatcatggcgaccacacccgtcctgtggatcctctacgccggacgcatcgtggccggcatcaccggcgccacaggtgcggttgctggcgcctatatcgccgacatcaccgatggggaagatcgggctcgccacttcgggctcatgagcgcttgtttcggcgtgggtatggtggcaggccccgtggccgggggactgttgggcgccatctccttgcatgcaccattccttgcggcggcggtgctcaacggcctcaacctactactgggctgcttcctaatgcaggagtcgcataagggagagcgtcgaccgatgcccttgagagccttcaacccagtcagctccttccggtgggcgcggggcatgactatcgtcgccgcacttatgactgtcttctttatcatgcaactcgtaggacaggtgccggcagcgctctgggtcattttcggcgaggaccgctttcgctggagcgcgacgatgatcggcctgtcgcttgcggtattcggaatcttgcacgccctcgctcaagccttcgtcactggtcccgccaccaaacgtttcggcgagaagcaggccattatcgccggcatggcggccgacgcgctgggctacgtcttgctggcgttcgcgacgcgaggctggatggccttccccattatgattcttctcgcttccggcggcatcgggatgcccgcgttgcaggccatgctgtccaggcaggtagatgacgaccatcagggacagcttcaaggatcgctcgcggctcttaccagcctaacttcgatcattggaccgctgatcgtcacggcgatttatgccgcctcggcgagcacatggaacgggttggcatggattgtaggcgccgccctataccttgtctgcctccccgcgttgcgtcgcggtgcatggagccgggccacctcgacctgaatggaagccggcggcacctcgctaacggattcaccactccaagaattggagccaatcaattcttgcggagaactgtgaatgcgcaaaccaacccgtttaaac

***E. coli r*eplicons**

**(1)** ColE1 (pMTL20)

ggcgcgcctcctttttgataatctcatgaccaaaatcccttaacgtgagttttcgttccactgagcgtcagaccccgtagaaaagatcaaaggatcttcttgagatcctttttttctgcgcgtaatctgctgcttgcaaacaaaaaaaccaccgctaccagcggtggtttgtttgccggatcaagagctaccaactctttttccgaaggtaactggcttcagcagagcgcagataccaaatactgttcttctagtgtagccgtagttaggccaccacttcaagaactctgtagcaccgcctacatacctcgctctgctaatcctgttaccagtggctgctgccagtggcgataagtcgtgtcttaccgggttggactcaagacgatagttaccggataaggcgcagcggtcgggctgaacggggggttcgtgcacacagcccagcttggagcgaacgacctacaccgaactgagatacctacagcgtgagctatgagaaagcgccacgcttcccgaagggagaaaggcggacaggtatccggtaagcggcagggtcggaacaggagagcgcacgagggagcttccagggggaaacgcctggtatctttatagtcctgtcgggtttcgccacctctgacttgagcgtcgatttttgtgatgctcgtcaggggggcggagcctatggaaaaacgccagcaacgcggcctttttacggttcctggccttttgctggccttttgctcacatgttctttcctgcgttatcccctgattctgtggataaccgtattaccgcctttgagtgagctgataccgctcgccgcagccgaacgaccgagcgcagcgagtcagtgagcgaggaagcggaagagcgcccaatacgcagggcccggccggcc

**(2)** ColE1 (pBR322) + oriT

ggcgcgcccggtcagttcagtaatttcctgcatttgcctgtttccagtcggtagatattccacaaaacagcagggaagcagcgcttttccgctgcataaccctgcttcggggtcattatagcgattttttcggtatatccatcctttttcgcacgatatacaggattttgccaaagggttcgtgtagactttccttggtgtatccaacggcgtcagccgggcaggataggtgaagtaggcccacccgcgagcgggtgttccttcttcactgtcccttattcgcacctggcggtgctcaacgggaatcctgctctgcgaggctggccggctaccgccggcgtaacagatgagggcaagcggatggctgatgaaaccaagccaaccaggaagggcagcccacctatcaaggtgtactgccttccagacgaacgaagagcgattgaggaaaaggcggcggcggccccggatctatgcggtgtgaaataccgcacagatgcgtaaggagaaaataccgcatcaggcgctcttccgcttcctcgctcactgactcgctgcgctcggtcgttcggctgcggcgagcggtatcagctcactcaaaggcggtaatacggttatccacagaatcaggggataacgcaggaaagaacatgtgagcaaaaggccagcaaaaggccaggaaccgtaaaaaggccgcgttgctggcgtttttccataggctccgcccccctgacgagcatcacaaaaatcgacgctcaagtcagaggtggcgaaacccgacaggactataaagataccaggcgtttccccctggaagctccctcgtgcgctctcctgttccgaccctgccgcttaccggatacctgtccgcctttctcccttcgggaagcgtggcgctttctcatagctcacgctgtaggtatctcagttcggtgtaggtcgttcgctccaagctgggctgtgtgcacgaaccccccgttcagcccgaccgctgcgccttatccggtaactatcgtcttgagtccaacccggtaagacacgacttatcgccactggcagcagccactggtaacaggattagcagagcgaggtatgtaggcggtgctacagagttcttgaagtggtggcctaactacggctacactagaaggacagtatttggtatctgcgctctgctgaagccagttaccttcggaaaaagagttggtagctcttgatccggcaaacaaaccaccgctggtagcggtggtttttttgtttgcaagcagcagattacgcgcagaaaaaaaggatctcaagaagatcctttgatcttttctacggggtctgacgctcagtggaacgaaaactcacgttaagggattttggtcatgagattatcaaaaaggatcttcacctagatccggccggcc

**(3)** p15a

ggcgcgccaagatgatcttcttgagatcgttttggtctgcgcgtaatctcttgctctgaaaacgaaaaaaccgccttgcagggcggtttttcgaaggttctctgagctaccaactctttgaaccgaggtaactggcttggaggagcgcagtcaccaaaacttgtcctttcagtttagccttaaccggcgcatgacttcaagactaactcctctaaatcaattaccagtggctgctgccagtggtgcttttgcatgtctttccgggttggactcaagacgatagttaccggataaggcgcagcggtcggactgaacggggggttcgtgcatacagtccagcttggagcgaactgcctacccggaactgagtgtcaggcgtggaatgagacaaacgcggccataacagcggaatgacaccggtaaaccgaaaggcaggaacaggagagcgcacgagggagccgccagggggaaacgcctggtatctttatagtcctgtcgggtttcgccaccactgatttgagcgtcagatttcgtgatgcttgtcaggggggcggagcctatggaaaaacggctttgccgcggccctctcacttccctgttaagtatcttcctggcatcttccaggaaatctccgccccgttcgtaagccatttccgctcgccgcagtcgaacgaccgagcgtagcgagtggccggcc

**Application specific module**

**(1)** MCS

cctgcaggataaaaaaattgtagataaattttataaaatagttttatctacaatttttttatcaggaaacagctatgaccgcggccgctgtatccatatgaccatgattacgaattcgagctcggtacccggggatcctctagagtcgacgtcacgcgtccatggagatctcgaggcctgcagacatgcaagcttggcactggccgtcgttttacaacgtcgtgactgggaaaaccctggcgttacccaacttaatcgccttgcagcacatccccctttcgccagctggcgtaatagcgaagaggcccgcaccgatcgcccttcccaacagttgcgcagcctgaatggcgaatggcgctagcataaaaataagaagcctgcatttgcaggcttcttatttttatggcgcgcc

**(2)** *P_BAD_*+MCS (promoter)

cctgcaggataaaaaaattgtagataaattttataaaatagttttatctacaatttttttatcaggaaacagctatgaccgcggccgcttatgacaacttgacggctacatcattcactttttcttcacaaccggcacggaactcgctcgggctggccccggtgcattttttaaatacccgcgagaaatagagttgatcgtcaaaaccaacattgcgaccgacggtggcgataggcatccgggtggtgctcaaaagcagcttcgcctggctgatacgttggtcctcgcgccagcttaagacgctaatccctaactgctggcggaaaagatgtgacagacgcgacggcgacaagcaaacatgctgtgcgacgctggcgatatcaaaattgctgtctgccaggtgatcgctgatgtactgacaagcctcgcgtacccgattatccatcggtggatggagcgactcgttaatcgcttccatgcgccgcagtaacaattgctcaagcagatttatcgccagcagctccgaatagcgcccttccccttgcccggcgttaatgatttgcccaaacaggtcgctgaaatgcggctggtgcgcttcatccgggcgaaagaaccccgtattggcaaatattgacggccagttaagccattcatgccagtaggcgcgcggacgaaagtaaacccactggtgataccattcgcgagcctccggatgacgaccgtagtgatgaatctctcctggcgggaacagcaaaatatcacccggtcggcaaacaaattctcgtccctgatttttcaccaccccctgaccgcgaatggtgagattgagaatataacctttcattcccagcggtcggtcgataaaaaaatcgagataaccgttggcctcaatcggcgttaaacccgccaccagatgggcattaaacgagtatcccggcagcaggggatcattttgcgcttcagccatacttttcatactcccgccattcagagaagaaaccaattgtccatattgcatcagacattgccgtcactgcgtcttttactggctcttctcgctaaccaaaccggtaaccccgcttattaaaagcattctgtaacaaagcgggaccaaagccatgacaaaaacgcgtaacaaaagtgtctataatcacggcagaaaagtccacattgattatttgcacggcgtcacactttgctatgccatagcatttttatccataagattagcggattctacctgacgctttttatcgcaactctctactgtttctccatacccgtttttttgggaattcaaaagatcttttaagaaggagatatacatatgaccatgattacgaattcgagctcggtacccggggatcctctagagtcgacgtcacgcgtccatggagatctcgaggcctgcagacatgcaagcttggcactggccgtcgttttacaacgtcgtgactgggaaaaccctggcgttacccaacttaatcgccttgcagcacatccccctttcgccagctggcgtaatagcgaagaggcccgcaccgatcgcccttcccaacagttgcgcagcctgaatggcgaatggcgctagcataaaaataagaagcctgcatttgcaggcttcttatttttatggcgcgcc

**(3)** *P_phaC_*+MCS (promoter)

cctgcaggataaaaaaattgtagataaattttataaaatagttttatctacaatttttttatcaggaaacagctatgaccgcggccgctcatccttctcgcctatgctctggggcctcggcagatgcgagcgctgcataccgtccggtaggtcgggaagcgtgcagtgccgaggcggattcccgcattgacagcgcgtgcgttgcaaggcaacaatggactcaaatgtctcggaatcgctgacgattcccaggtttctccggcaagcatagcgcatggcgtctccatgcgagaatgtcgcgcttgccggataaaaggggagccgctatcggaatggacgcaagccacggccgcagcaggtgcggtcgagggcttccagccagttccagggcagatgtgccggcagaccctcccgctttgggggaggcgcaagccgggtccattcggatagcatctccccatgcaaagtgccggccagggcaatgcccggagccggttcgaatagtgacggcagagagacaatcaacatatgaccatgattacgaattcgagctcggtacccggggatcctctagagtcgacgtcacgcgtccatggagatctcgaggcctgcagacatgcaagcttggcactggccgtcgttttacaacgtcgtgactgggaaaaccctggcgttacccaacttaatcgccttgcagcacatccccctttcgccagctggcgtaatagcgaagaggcccgcaccgatcgcccttcccaacagttgcgcagcctgaatggcgaatggcgctagcataaaaataagaagcctgcatttgcaggcttcttatttttatggcgcgcc

**(4)** *rfp* (reporter)

cctgcaggataaaaaaattgtagataaattttataaaatagttttatctacaatttttttatcaggaaacagctatgaccgcggccgctgtatccatatggcgagtagcgaagacgttatcaaagagttcatgcgtttcaaagttcgtatggaaggttccgttaacggtcacgagttcgaaatcgaaggtgaaggtgaaggtcgtccgtacgaaggtacccagaccgctaaactgaaagttaccaaaggtggtccgctgccgttcgcttgggacatcctgtccccgcagttccagtacggttccaaagcttacgttaaacacccggctgacatcccggactacctgaaactgtccttcccggaaggtttcaaatgggaacgtgttatgaacttcgaagacggtggtgttgttaccgttacccaggactcctccctgcaagacggtgagttcatctacaaagttaaactgcgtggtaccaacttcccgtccgacggtccggttatgcagaaaaaaaccatgggttgggaagcttccaccgaacgtatgtacccggaagacggtgctctgaaaggtgaaatcaaaatgcgtctgaaactgaaagacggtggtcactacgacgctgaagttaaaaccacctacatggctaaaaaaccggttcagctgccgggtgcttacaaaaccgacatcaaactggacatcacctcccacaacgaagactacaccatcgttgaacagtacgaacgtgctgaaggtcgtcactccaccggtgcttaaatcgcccttcccaacagttgcgcagcctgaatggcgaatggcgctagcataaaaataagaagcctgcatttgcaggcttcttatttttatggcgcgcc

**Selected Plasmids**

pMTL71101

cctgcaggataaaaaaattgtagataaattttataaaatagttttatctacaatttttttatcaggaaacagctatgaccgcggccgctgtatccatatgaccatgattacgaattcgagctcggtacccggggatcctctagagtcgacgtcacgcgtccatggagatctcgaggcctgcagacatgcaagcttggcactggccgtcgttttacaacgtcgtgactgggaaaaccctggcgttacccaacttaatcgccttgcagcacatccccctttcgccagctggcgtaatagcgaagaggcccgcaccgatcgcccttcccaacagttgcgcagcctgaatggcgaatggcgctagcataaaaataagaagcctgcatttgcaggcttcttatttttatggcgcgccggccggccagtgggcaagttgaaaaattcacaaaaatgtggtataatatctttgttcattagagcgataaacttgaatttgagagggaacttagatggtatttgaaaaaattgataaaaatagttggaacagaaaagagtattttgaccactactttgcaagtgtaccttgtacctacagcatgaccgttaaagtggatatcacacaaataaaggaaaagggaatgaaactatatcctgcaatgctttattatattgcaatgattgtaaaccgccattcagagtttaggacggcaatcaatcaagatggtgaattggggatatatgatgagatgataccaagctatacaatatttcacaatgatactgaaacattttccagcctttggactgagtgtaagtctgactttaaatcatttttagcagattatgaaagtgatacgcaacggtatggaaacaatcatagaatggaaggaaagccaaatgctccggaaaacatttttaatgtatctatgataccgtggtcaaccttcgatggctttaatctgaatttgcagaaaggatatgattatttgattcctatttttactatggggaaatattataaagaagataacaaaattatacttcctttggcaattcaagttcatcacgcagtatgtgacggatttcacatttgccgttttgtaaacgaattgcaggaattgataaatagttaacttcaggtttgtctgtaactaaaaacaagtatttaagcaaaaacatcgtagaaatacggtgttttttgttaccctaagtttaaacgtgctacgcctgaataagtgataataagcggatgaatggcagaaattcgaaagcaaattcgacccggtcgtcggttcagggcagggtcgttaaatagccgcttatgtctattgctggtttaccggtttattgactaccggaagcagtgtgaccgtgtgcttctcaaatgcctgaggccagtttgctcaggctctccccgtggaggtaataattgacgatatgatcatttattctgcctcccagagcctgataaaaacggtgaatccgttagcgaggtgccgccggcttccattcaggtcgaggtggcccggctccatgcaccgcgacgcaacgcggggaggcagacaaggtatagggcggcgaggcggctacagccgatagtctggaacagcgcacttacgggttgctgcgcaacccaagtgctaccggcgcggcagcgtgacccgtgtcggcggctccaacggctcgccatcgtccagaaaacacggctcatcgggcatcggcaggcgctgctgcccgcgccgttcccattcctccgtttcggtcaaggctggcaggtctggttccatgcccggaatgccgggctggctgggcggctcctcgccggggccggtcggtagttgctgctcgcccggatacagggtcgggatgcggcgcaggtcgccatgccccaacagcgattcgtcctggtcgtcgtgatcaaccaccacggcggcactgaacaccgacaggcgcaactggtcgcggggctggccccacgccacgcggtcattgaccacgtaggccgacacggtgccggggccgttgagcttcacgacggagatccagcgctcggccaccaagtccttgactgcgtattggaccgtccgcaaagaacgtccgatgagcttggaaagtgtcttctggctgaccaccacggcgttctggtggcccatctgcgccacgaggtgatgcagcagcattgccgccgtgggtttcctcgcaataagcccggcccacgcctcatgcgctttgcgttccgtttgcacccagtgaccgggcttgttcttggcttgaatgccgatttctctggactgcgtggccatgcttatctccatgcggtaggggtgccgcacggttgcggcaccatgcgcaatcagctgcaacttttcggcagcgcgacaacaattatgcgttgcgtaaaagtggcagtcaattacagattttctttaacctacgcaatgagctattgcggggggtgccgcaatgagctgttgcgtaccccccttttttaagttgttgatttttaagtctttcgcatttcgccctatatctagttctttggtgcccaaagaagggcacccctgcggggttcccccacgccttcggcgcggctccccctccggcaaaaagtggcccctccggggcttgttgatcgactgcgcggccttcggccttgcccaaggtggcgctgcccccttggaacccccgcactcgccgccgtgaggctcggggggcaggcgggcgggcttcgccttcgactgcccccactcgcataggcttgggtcgttccaggcgcgtcaaggccaagccgctgcgcggtcgctgcgcgagccttgacccgccttccacttggtgtccaaccggcaagcgaagcgcgcaggccgcaggccggaggcttttccccagagaaaattaaaaaaattgatggggcaaggccgcaggccgcgcagttggagccggtgggtatgtggtcgaaggctgggtagccggtgggcaatccctgtggtcaagctcgtgggcaggcgcagcctgtccatcagcttgtccagcagggttgtccacgggccgagcgaagcgagccagccggtggccgctcgcggccatcgtccacatatccacgggctggcaagggagcgcagcgaccgcgcagggcgaagcccggagagcaagcccgtagggcgccgcagccgccgtaggcggtcacgactttgcgaagcaaagtctagtgagtatactcaagcattgagtggcccgccggaggcaccgccttgcgctgcccccgtcgagccggttggacaccaaaagggaggggcaggcatggcggcatacgcgatcatgcgatgcaagaagctggcgaaaatgggcaacgtggcggccagtctcaagcacgcctaccgcgagcgcgagacgcccaacgctgacgccagcaggacgccagagaacgagcactgggcggccagcagcaccgatgaagcgatgggccgactgcgcgagttgctgccagagaagcggcgcaaggacgctgtgttggcggtcgagtacgtcatgacggccagcccggaatggtggaagtcggccagccaagaacagcaggcggcgttcttcgagaaggcgcacaagtggctggcggacaagtacggggcggatcgcatcgtgacggccagcatccaccgtgacgaaaccagcccgcacatgaccgcgttcgtggtgccgctgacgcaggacggcaggctgtcggccaaggagttcatcggcaacaaagcgcagatgacccgcgaccagaccacgtttgccgccgctgtggccgatctagggctgcaacggggcatcgagggcagcaaggcacgtcacacgcgcattcaggcgttctacgaggccctggagcggccaccagtgggccacgtcaccatcagcccgcaagcggtcgagccacgcgcctatgcaccgcagggattggccgaaaagctgggaatctcaaagcgcgttgagacgccggaagccgtggccgaccggctgacaaaagcggttcggcaggggtatgagcctgccctacaggccgccgcaggagcgcgtgagatgcgcaagaaggccgatcaagcccaagagacggcccgagaccttcgggagcgcctgaagcccgttctggacgccctggggccgttgaatcgggatatgcaggccaaggccgccgcgatcatcaaggccgtgggcgaaaagctgctgacggaacagcgggaagtccagcgccagaaacaggcccagcgccagcaggaacgcgggcgcgcacatttccccgaaaagtgccacctgacgtctaagaaaccattattatcatgacattaacctataaaaataggcgtatcacgaggccctttcgtcttcgaataaatacctgtgacggaagatcacttcgcagaataaataaatcctggtgtccctgttgataccgggaagccctgggccaacttttggcgaaaatgagacgttgatcggcacgtaagaggttccaactttcaccataatgaaataagatcactaccgggcgtattttttgagttatcgagattttcaggagctaaggaagctaaaatggagaaaaaaatcactggatataccaccgttgatatatcccaatggcatcgtaaagaacattttgaggcatttcagtcagttgctcaatgtacctataaccagaccgttcagctggatattacggcctttttagcttttgccattctcaccggattcagtcgtcactcatggtgatttctcacttgataaccttatttttgacgaggggaaattaataggttgtattgatgttggacgagtcggaatcgcagaccgataccaggatcttgccatcctatggaactgcctcggtgagttttctccttcattacagaaacggctttttcaaaaatatggtattgataatcctgatatgaataaattgcagtttcatttgatgctcgatgagtttttctaatcagaattggttaattggttgtaacactggcagagcattacgctgacttgacgggacggcggctttgttgaataaatcgaacttttgctgagttgaaggatcagatcacgcatcttcccgacaacgcagaccgttccgtggcaaagcaaaagttcaaaatcaccaactggtccacctacaacaaagctctcatcaaccgtggctccctcactttctggctggatgatggggcgattcaggcctggtatgagtcagcaacaccttcttcacgaggcagacctcatcag

pMTL71201

cctgcaggataaaaaaattgtagataaattttataaaatagttttatctacaatttttttatcaggaaacagctatgaccgcggccgctgtatccatatgaccatgattacgaattcgagctcggtacccggggatcctctagagtcgacgtcacgcgtccatggagatctcgaggcctgcagacatgcaagcttggcactggccgtcgttttacaacgtcgtgactgggaaaaccctggcgttacccaacttaatcgccttgcagcacatccccctttcgccagctggcgtaatagcgaagaggcccgcaccgatcgcccttcccaacagttgcgcagcctgaatggcgaatggcgctagcataaaaataagaagcctgcatttgcaggcttcttatttttatggcgcgccggccggccaggtggcacttttcggggaaatgtgcgcggaacccctatttgtttatttttctaaatacattcaaatatgtatccgctcatgagacaataaccctgataaatgcttcaataatattgaaaaaggaagagtatgagtattcaacatttccgtgtcgcccttattcccttttttgcggcattttgccttcctgtttttgctcacccagaaacgctggtgaaagtaaaagatgctgaagatcagttgggtgcacgagtgggttacatcgaactggatctcaacagcggtaagatccttgagagttttcgccccgaagaacgttttccaatgatgagcacttttaaagttctgctatgtggcgcggtattatcccgtattgacgccgggcaagagcaactcggtcgccgcatacactattctcagaatgacttggttgagtactcaccagtcacagaaaagcatcttacggatggcatgacagtaagagaattatgcagtgctgccataaccatgagtgataacactgcggccaacttacttctgacaacgatcggaggaccgaaggagctaaccgcttttttgcacaacatgggggatcatgtaactcgccttgatcgttgggaaccggagctgaatgaagccataccaaacgacgagcgtgacaccacgatgcctgtagcaatggcaacaacgttgcgcaaactattaactggcgaactacttactctagcttcccggcaacaattaatagactggatggaggcggataaagttgcaggaccacttctgcgctcggcccttccggctggctggtttattgctgataaatctggagccggtgagcgtgggtctcgcggtatcattgcagcactggggccagatggtaagccctcccgtatcgtagttatctacacgacggggagtcaggcaactatggatgaacgaaatagacagatcgctgagataggtgcctcactgattaagcattggtaactgtcagaccaagtttactcatatatactttagattgatttaaaacttcatttttaatttaaaaggatctaggtgaagatcctttttgataatctcatgaccaaaatcccttaacgtgagttttcgttccactgagcgtcagagtttaaacgtgctacgcctgaataagtgataataagcggatgaatggcagaaattcgaaagcaaattcgacccggtcgtcggttcagggcagggtcgttaaatagccgcttatgtctattgctggtttaccggtttattgactaccggaagcagtgtgaccgtgtgcttctcaaatgcctgaggccagtttgctcaggctctccccgtggaggtaataattgacgatatgatcatttattctgcctcccagagcctgataaaaacggtgaatccgttagcgaggtgccgccggcttccattcaggtcgaggtggcccggctccatgcaccgcgacgcaacgcggggaggcagacaaggtatagggcggcgaggcggctacagccgatagtctggaacagcgcacttacgggttgctgcgcaacccaagtgctaccggcgcggcagcgtgacccgtgtcggcggctccaacggctcgccatcgtccagaaaacacggctcatcgggcatcggcaggcgctgctgcccgcgccgttcccattcctccgtttcggtcaaggctggcaggtctggttccatgcccggaatgccgggctggctgggcggctcctcgccggggccggtcggtagttgctgctcgcccggatacagggtcgggatgcggcgcaggtcgccatgccccaacagcgattcgtcctggtcgtcgtgatcaaccaccacggcggcactgaacaccgacaggcgcaactggtcgcggggctggccccacgccacgcggtcattgaccacgtaggccgacacggtgccggggccgttgagcttcacgacggagatccagcgctcggccaccaagtccttgactgcgtattggaccgtccgcaaagaacgtccgatgagcttggaaagtgtcttctggctgaccaccacggcgttctggtggcccatctgcgccacgaggtgatgcagcagcattgccgccgtgggtttcctcgcaataagcccggcccacgcctcatgcgctttgcgttccgtttgcacccagtgaccgggcttgttcttggcttgaatgccgatttctctggactgcgtggccatgcttatctccatgcggtaggggtgccgcacggttgcggcaccatgcgcaatcagctgcaacttttcggcagcgcgacaacaattatgcgttgcgtaaaagtggcagtcaattacagattttctttaacctacgcaatgagctattgcggggggtgccgcaatgagctgttgcgtaccccccttttttaagttgttgatttttaagtctttcgcatttcgccctatatctagttctttggtgcccaaagaagggcacccctgcggggttcccccacgccttcggcgcggctccccctccggcaaaaagtggcccctccggggcttgttgatcgactgcgcggccttcggccttgcccaaggtggcgctgcccccttggaacccccgcactcgccgccgtgaggctcggggggcaggcgggcgggcttcgccttcgactgcccccactcgcataggcttgggtcgttccaggcgcgtcaaggccaagccgctgcgcggtcgctgcgcgagccttgacccgccttccacttggtgtccaaccggcaagcgaagcgcgcaggccgcaggccggaggcttttccccagagaaaattaaaaaaattgatggggcaaggccgcaggccgcgcagttggagccggtgggtatgtggtcgaaggctgggtagccggtgggcaatccctgtggtcaagctcgtgggcaggcgcagcctgtccatcagcttgtccagcagggttgtccacgggccgagcgaagcgagccagccggtggccgctcgcggccatcgtccacatatccacgggctggcaagggagcgcagcgaccgcgcagggcgaagcccggagagcaagcccgtagggcgccgcagccgccgtaggcggtcacgactttgcgaagcaaagtctagtgagtatactcaagcattgagtggcccgccggaggcaccgccttgcgctgcccccgtcgagccggttggacaccaaaagggaggggcaggcatggcggcatacgcgatcatgcgatgcaagaagctggcgaaaatgggcaacgtggcggccagtctcaagcacgcctaccgcgagcgcgagacgcccaacgctgacgccagcaggacgccagagaacgagcactgggcggccagcagcaccgatgaagcgatgggccgactgcgcgagttgctgccagagaagcggcgcaaggacgctgtgttggcggtcgagtacgtcatgacggccagcccggaatggtggaagtcggccagccaagaacagcaggcggcgttcttcgagaaggcgcacaagtggctggcggacaagtacggggcggatcgcatcgtgacggccagcatccaccgtgacgaaaccagcccgcacatgaccgcgttcgtggtgccgctgacgcaggacggcaggctgtcggccaaggagttcatcggcaacaaagcgcagatgacccgcgaccagaccacgtttgccgccgctgtggccgatctagggctgcaacggggcatcgagggcagcaaggcacgtcacacgcgcattcaggcgttctacgaggccctggagcggccaccagtgggccacgtcaccatcagcccgcaagcggtcgagccacgcgcctatgcaccgcagggattggccgaaaagctgggaatctcaaagcgcgttgagacgccggaagccgtggccgaccggctgacaaaagcggttcggcaggggtatgagcctgccctacaggccgccgcaggagcgcgtgagatgcgcaagaaggccgatcaagcccaagagacggcccgagaccttcgggagcgcctgaagcccgttctggacgccctggggccgttgaatcgggatatgcaggccaaggccgccgcgatcatcaaggccgtgggcgaaaagctgctgacggaacagcgggaagtccagcgccagaaacaggcccagcgccagcaggaacgcgggcgcgcacatttccccgaaaagtgccacctgacgtctaagaaaccattattatcatgacattaacctataaaaataggcgtatcacgaggccctttcgtcttcgaataaatacctgtgacggaagatcacttcgcagaataaataaatcctggtgtccctgttgataccgggaagccctgggccaacttttggcgaaaatgagacgttgatcggcacgtaagaggttccaactttcaccataatgaaataagatcactaccgggcgtattttttgagttatcgagattttcaggagctaaggaagctaaaatggagaaaaaaatcactggatataccaccgttgatatatcccaatggcatcgtaaagaacattttgaggcatttcagtcagttgctcaatgtacctataaccagaccgttcagctggatattacggcctttttagcttttgccattctcaccggattcagtcgtcactcatggtgatttctcacttgataaccttatttttgacgaggggaaattaataggttgtattgatgttggacgagtcggaatcgcagaccgataccaggatcttgccatcctatggaactgcctcggtgagttttctccttcattacagaaacggctttttcaaaaatatggtattgataatcctgatatgaataaattgcagtttcatttgatgctcgatgagtttttctaatcagaattggttaattggttgtaacactggcagagcattacgctgacttgacgggacggcggctttgttgaataaatcgaacttttgctgagttgaaggatcagatcacgcatcttcccgacaacgcagaccgttccgtggcaaagcaaaagttcaaaatcaccaactggtccacctacaacaaagctctcatcaaccgtggctccctcactttctggctggatgatggggcgattcaggcctggtatgagtcagcaacaccttcttcacgaggcagacctcag

pMTL71301

caggataaaaaaattgtagataaattttataaaatagttttatctacaatttttttatcaggaaacagctatgaccgcggccgctgtatccatatgaccatgattacgaattcgagctcggtacccggggatcctctagagtcgacgtcacgcgtccatggagatctcgaggcctgcagacatgcaagcttggcactggccgtcgttttacaacgtcgtgactgggaaaaccctggcgttacccaacttaatcgccttgcagcacatccccctttcgccagctggcgtaatagcgaagaggcccgcaccgatcgcccttcccaacagttgcgcagcctgaatggcgaatggcgctagcataaaaataagaagcctgcatttgcaggcttcttatttttatggcgcgccggccggccgcaggagcgccagaaggccgccagagaggccgagcgcggccgtgaggcttggacgctagggcagggcatgaaaaagcccgtagcgggctgctacgggcgtctgacgcggtggaaagggggaggggatgttgtctacatggctctgctgtagtgagtgggttgcgctccggcagcggtcctgatcaatcgtcaccctttctcggtccttcaacgttcctgacaacgagcctccttttcgccaatccatcgacaatcaccgcgagtccctgctcgaacgctgcgtccggaccggcttcgtcgaaggcgtctatcgcggcccgcaacagcggcgagagcggagcctgttcaacggtgccgccgcgctcgccggcatcgctgtcgccggcctgctcctcaagcacggccccaacagtgaagtagctgattgtcatcagcgcattgacggcgtccccggccgaaaaacccgcctcgcagaggaagcgaagctgcgcgtcggccgtttccatctgcggtgcgcccggtcgcgtgccggcatggatgcgcgcgccatcgcggtaggcgagcagcgcctgcctgaagctgcgggcattcccgatcagaaatgagcgccagtcgtcgtcggctctcggcaccgaatgcgtatgattctccgccagcatggcttcggccagtgcgtcgagcagcgcccgcttgttcctgaagtgccagtaaagcgccggctgctgaacccccaaccgttccgccagtttgcgtgtcgtcagaccgtctacgccgacctcgttcaacaggtccagggcggcacggatcactgtattcggctgcaactttgtcatgcttgacactttatcactgataaacataatatgtccaccaacttatcagtgataaagaatccgcgcgttcaatcggaccagcggaggctggtccggaggccagacgtgaaacccaacatacccctgatcgtaattctgagcactgtcgcgctcgacgctgtcggcatcggcctgattatgccggtgctgccgggcctcctgcgcgatctggttcactcgaacgacgtcaccgcccactatggcattctgctggcgctgtatgcgttggtgcaatttgcctgcgcacctgtgctgggcgcgctgtcggatcgtttcgggcggcggccaatcttgctcgtctcgctggccggcgccactgtcgactacgccatcatggcgacagcgcctttcctttgggttctctatatcgggcggatcgtggccggcatcaccggggcgactggggcggtagccggcgcttatattgccgatatcactgatggcgatgagcgcgcgcggcacttcggcttcatgagcgcctgtttcgggttcgggatggtcgcgggacctgtgctcggtgggctgatgggcggtttctccccccacgctccgttcttcgccgcggcagccttgaacggcctcaatttcctgacgggctgtttccttttgccggagtcgcacaaaggcgaacgccggccgttacgccgggaggctctcaacccgctcgcttcgttccggtgggcccggggcatgaccgtcgtcgccgccctgatggcggtcttcttcatcatgcaacttgtcggacaggtgccggccgcgctttgggtcattttcggcgaggatcgctttcactgggacgcgaccacgatcggcatttcgcttgccgcatttggcattctgcattcactcgcccaggcagtgatcaccggccctgtagccgcccggctcggcgaaaggcgggcactcatgctcggaatgattgccgacggcacaggctacatcctgcttgccttcgcgacacggggatggatggcgttcccgatcatggtcctgcttgcttcgggtggcatcggaatgccggcgctgcaagcaatgttgtccaggcaggtggatgaggaacgtcaggggcagctgcaaggctcactggcggcgctcaccagcctgacctcgatcgtcggacccctcctcttcacggcgatctatgcggcttctataacaacgtggaacgggtgggcatggattgcgggcgctgccctctacttgctctgcctgccggcgctgcgtcgcgggctttggagcggcgcagggcaacgagccgatcgctgatcgtggaaacgataggaaacgtggccaatatggacaacttcttcgcccccgttttcaccatgggcaaatattacacgcaaggcgacaaggtgctgatgccgctggcgattcaggttcatcatgccgtttgtgatggcttccatgtcggcagaatgcttaatgaattacaacagtttttatgcatgcgcccaatacgcaaaccgcctctccccgcgcgttggccgattcattaatgcagctggcacgacaggtttcccgactggaaagcgggcagtgaacgcaacgcaattaatgtgagttagctcactcattaggcaccccaggctttacactttatgcttccggctcgtatgttgtgtggaattgtgagcggtttaaacgtgctacgcctgaataagtgataataagcggatgaatggcagaaattcgaaagcaaattcgacccggtcgtcggttcagggcagggtcgttaaatagccgcttatgtctattgctggtttaccggtttattgactaccggaagcagtgtgaccgtgtgcttctcaaatgcctgaggccagtttgctcaggctctccccgtggaggtaataattgacgatatgatcatttattctgcctcccagagcctgataaaaacggtgaatccgttagcgaggtgccgccggcttccattcaggtcgaggtggcccggctccatgcaccgcgacgcaacgcggggaggcagacaaggtatagggcggcgaggcggctacagccgatagtctggaacagcgcacttacgggttgctgcgcaacccaagtgctaccggcgcggcagcgtgacccgtgtcggcggctccaacggctcgccatcgtccagaaaacacggctcatcgggcatcggcaggcgctgctgcccgcgccgttcccattcctccgtttcggtcaaggctggcaggtctggttccatgcccggaatgccgggctggctgggcggctcctcgccggggccggtcggtagttgctgctcgcccggatacagggtcgggatgcggcgcaggtcgccatgccccaacagcgattcgtcctggtcgtcgtgatcaaccaccacggcggcactgaacaccgacaggcgcaactggtcgcggggctggccccacgccacgcggtcattgaccacgtaggccgacacggtgccggggccgttgagcttcacgacggagatccagcgctcggccaccaagtccttgactgcgtattggaccgtccgcaaagaacgtccgatgagcttggaaagtgtcttctggctgaccaccacggcgttctggtggcccatctgcgccacgaggtgatgcagcagcattgccgccgtgggtttcctcgcaataagcccggcccacgcctcatgcgctttgcgttccgtttgcacccagtgaccgggcttgttcttggcttgaatgccgatttctctggactgcgtggccatgcttatctccatgcggtaggggtgccgcacggttgcggcaccatgcgcaatcagctgcaacttttcggcagcgcgacaacaattatgcgttgcgtaaaagtggcagtcaattacagattttctttaacctacgcaatgagctattgcggggggtgccgcaatgagctgttgcgtaccccccttttttaagttgttgatttttaagtctttcgcatttcgccctatatctagttctttggtgcccaaagaagggcacccctgcggggttcccccacgccttcggcgcggctccccctccggcaaaaagtggcccctccggggcttgttgatcgactgcgcggccttcggccttgcccaaggtggcgctgcccccttggaacccccgcactcgccgccgtgaggctcggggggcaggcgggcgggcttcgccttcgactgcccccactcgcataggcttgggtcgttccaggcgcgtcaaggccaagccgctgcgcggtcgctgcgcgagccttgacccgccttccacttggtgtccaaccggcaagcgaagcgcgcaggccgcaggccggaggcttttccccagagaaaattaaaaaaattgatggggcaaggccgcaggccgcgcagttggagccggtgggtatgtggtcgaaggctgggtagccggtgggcaatccctgtggtcaagctcgtgggcaggcgcagcctgtccatcagcttgtccagcagggttgtccacgggccgagcgaagcgagccagccggtggccgctcgcggccatcgtccacatatccacgggctggcaagggagcgcagcgaccgcgcagggcgaagcccggagagcaagcccgtagggcgccgcagccgccgtaggcggtcacgactttgcgaagcaaagtctagtgagtatactcaagcattgagtggcccgccggaggcaccgccttgcgctgcccccgtcgagccggttggacaccaaaagggaggggcaggcatggcggcatacgcgatcatgcgatgcaagaagctggcgaaaatgggcaacgtggcggccagtctcaagcacgcctaccgcgagcgcgagacgcccaacgctgacgccagcaggacgccagagaacgagcactgggcggccagcagcaccgatgaagcgatgggccgactgcgcgagttgctgccagagaagcggcgcaaggacgctgtgttggcggtcgagtacgtcatgacggccagcccggaatggtggaagtcggccagccaagaacagcaggcggcgttcttcgagaaggcgcacaagtggctggcggacaagtacggggcggatcgcatcgtgacggccagcatccaccgtgacgaaaccagcccgcacatgaccgcgttcgtggtgccgctgacgcaggacggcaggctgtcggccaaggagttcatcggcaacaaagcgcagatgacccgcgaccagaccacgtttgccgccgctgtggccgatctagggctgcaacggggcatcgagggcagcaaggcacgtcacacgcgcattcaggcgttctacgaggccctggagcggccaccagtgggccacgtcaccatcagcccgcaagcggtcgagccacgcgcctatgcaccgcagggattggccgaaaagctgggaatctcaaagcgcgttgagacgccggaagccgtggccgaccggctgacaaaagcggttcggcaggggtatgagcctgccctacaggccgccgcaggagcgcgtgagatgcgcaagaaggccgatcaagcccaagagacggcccgagaccttcgggagcgcctgaagcccgttctggacgccctggggccgttgaatcgggatatgcaggccaaggccgccgcgatcatcaaggccgtgggcgaaaagctgctgacggaacagcgggaagtccagcgccagaaacaggcccagcgccagcaggaacgcgggcgcgcacatttccccgaaaagtgccacctgacgtctaagaaaccattattatcatgacattaacctataaaaataggcgtatcacgaggccctttcgtcttcgaataaatacctgtgacggaagatcacttcgcagaataaataaatcctggtgtccctgttgataccgggaagccctgggccaacttttggcgaaaatgagacgttgatcggcacgtaagaggttccaactttcaccataatgaaataagatcactaccgggcgtattttttgagttatcgagattttcaggagctaaggaagctaaaatggagaaaaaaatcactggatataccaccgttgatatatcccaatggcatcgtaaagaacattttgaggcatttcagtcagttgctcaatgtacctataaccagaccgttcagctggatattacggcctttttagcttttgccattctcaccggattcagtcgtcactcatggtgatttctcacttgataaccttatttttgacgaggggaaattaataggttgtattgatgttggacgagtcggaatcgcagaccgataccaggatcttgccatcctatggaactgcctcggtgagttttctccttcattacagaaacggctttttcaaaaatatggtattgataatcctgatatgaataaattgcagtttcatttgatgctcgatgagtttttctaatcagaattggttaattggttgtaacactggcagagcattacgctgacttgacgggacggcggctttgttgaataaatcgaacttttgctgagttgaaggatcagatcacgcatcttcccgacaacgcagaccgttccgtggcaaagcaaaagttcaaaatcaccaactggtccacctacaacaaagctctcatcaaccgtggctccctcactttctggctggatgatggggcgattcaggcctggtatgagtcagcaacaccttcttcacgaggcagacctcagcctg

pMTL71401

cctgcaggataaaaaaattgtagataaattttataaaatagttttatctacaatttttttatcaggaaacagctatgaccgcggccgctgtatccatatgaccatgattacgaattcgagctcggtacccggggatcctctagagtcgacgtcacgcgtccatggagatctcgaggcctgcagacatgcaagcttggcactggccgtcgttttacaacgtcgtgactgggaaaaccctggcgttacccaacttaatcgccttgcagcacatccccctttcgccagctggcgtaatagcgaagaggcccgcaccgatcgcccttcccaacagttgcgcagcctgaatggcgaatggcgctagcataaaaataagaagcctgcatttgcaggcttcttatttttatggcgcgccggccggccggatgaatgtcagctactgggctatctggacaagggaaaacgcaagcgcaaagagaaagcaggtagcttgcagtgggcttacatggcgatagctagactgggcggttttatggacagcaagcgaaccggaattgccagctggggcgccctctggtaaggttgggaagccctgcaaagtaaactggatggctttcttgccgccaaggatctgatggcgcaggggatcaagatctgatcaagagacaggatgaggatcgtttcgcatgattgaacaagatggattgcacgcaggttctccggccgcttgggtggagaggctattcggctatgactgggcacaacagacaatcggctgctctgatgccgccgtgttccggctgtcagcgcaggggcgcccggttctttttgtcaagaccgacctgtccggtgccctgaatgaactgcaggacgaggcagcgcggctatcgtggctggccacgacgggcgttccttgcgcagctgtgctcgacgttgtcactgaagcgggaagggactggctgctattgggcgaagtgccggggcaggatctcctgtcatctcaccttgctcctgccgagaaagtatccatcatggctgatgcaatgcggcggctgcatacgcttgatccggctacctgcccattcgaccaccaagcgaaacatcgcatcgagcgagcacgtactcggatggaagccggtcttgtcgatcaggatgatctggacgaagagcatcaggggctcgcgccagccgaactgttcgccaggctcaaggcgcgcatgcccgacggcgaggatctcgtcgtgacccatggcgatgcctgcttgccgaatatcatggtggaaaatggccgcttttctggattcatcgactgtggccggctgggtgtggcggaccgctatcaggacatagcgttggctacccgtgatattgctgaagagcttggcggcgaatgggctgaccgcttcctcgtgctttacggtatcgccgctcccgattcgcagcgcatcgccttctatcgccttcttgacgagttcttctgagtttaaacgtgctacgcctgaataagtgataataagcggatgaatggcagaaattcgaaagcaaattcgacccggtcgtcggttcagggcagggtcgttaaatagccgcttatgtctattgctggtttaccggtttattgactaccggaagcagtgtgaccgtgtgcttctcaaatgcctgaggccagtttgctcaggctctccccgtggaggtaataattgacgatatgatcatttattctgcctcccagagcctgataaaaacggtgaatccgttagcgaggtgccgccggcttccattcaggtcgaggtggcccggctccatgcaccgcgacgcaacgcggggaggcagacaaggtatagggcggcgaggcggctacagccgatagtctggaacagcgcacttacgggttgctgcgcaacccaagtgctaccggcgcggcagcgtgacccgtgtcggcggctccaacggctcgccatcgtccagaaaacacggctcatcgggcatcggcaggcgctgctgcccgcgccgttcccattcctccgtttcggtcaaggctggcaggtctggttccatgcccggaatgccgggctggctgggcggctcctcgccggggccggtcggtagttgctgctcgcccggatacagggtcgggatgcggcgcaggtcgccatgccccaacagcgattcgtcctggtcgtcgtgatcaaccaccacggcggcactgaacaccgacaggcgcaactggtcgcggggctggccccacgccacgcggtcattgaccacgtaggccgacacggtgccggggccgttgagcttcacgacggagatccagcgctcggccaccaagtccttgactgcgtattggaccgtccgcaaagaacgtccgatgagcttggaaagtgtcttctggctgaccaccacggcgttctggtggcccatctgcgccacgaggtgatgcagcagcattgccgccgtgggtttcctcgcaataagcccggcccacgcctcatgcgctttgcgttccgtttgcacccagtgaccgggcttgttcttggcttgaatgccgatttctctggactgcgtggccatgcttatctccatgcggtaggggtgccgcacggttgcggcaccatgcgcaatcagctgcaacttttcggcagcgcgacaacaattatgcgttgcgtaaaagtggcagtcaattacagattttctttaacctacgcaatgagctattgcggggggtgccgcaatgagctgttgcgtaccccccttttttaagttgttgatttttaagtctttcgcatttcgccctatatctagttctttggtgcccaaagaagggcacccctgcggggttcccccacgccttcggcgcggctccccctccggcaaaaagtggcccctccggggcttgttgatcgactgcgcggccttcggccttgcccaaggtggcgctgcccccttggaacccccgcactcgccgccgtgaggctcggggggcaggcgggcgggcttcgccttcgactgcccccactcgcataggcttgggtcgttccaggcgcgtcaaggccaagccgctgcgcggtcgctgcgcgagccttgacccgccttccacttggtgtccaaccggcaagcgaagcgcgcaggccgcaggccggaggcttttccccagagaaaattaaaaaaattgatggggcaaggccgcaggccgcgcagttggagccggtgggtatgtggtcgaaggctgggtagccggtgggcaatccctgtggtcaagctcgtgggcaggcgcagcctgtccatcagcttgtccagcagggttgtccacgggccgagcgaagcgagccagccggtggccgctcgcggccatcgtccacatatccacgggctggcaagggagcgcagcgaccgcgcagggcgaagcccggagagcaagcccgtagggcgccgcagccgccgtaggcggtcacgactttgcgaagcaaagtctagtgagtatactcaagcattgagtggcccgccggaggcaccgccttgcgctgcccccgtcgagccggttggacaccaaaagggaggggcaggcatggcggcatacgcgatcatgcgatgcaagaagctggcgaaaatgggcaacgtggcggccagtctcaagcacgcctaccgcgagcgcgagacgcccaacgctgacgccagcaggacgccagagaacgagcactgggcggccagcagcaccgatgaagcgatgggccgactgcgcgagttgctgccagagaagcggcgcaaggacgctgtgttggcggtcgagtacgtcatgacggccagcccggaatggtggaagtcggccagccaagaacagcaggcggcgttcttcgagaaggcgcacaagtggctggcggacaagtacggggcggatcgcatcgtgacggccagcatccaccgtgacgaaaccagcccgcacatgaccgcgttcgtggtgccgctgacgcaggacggcaggctgtcggccaaggagttcatcggcaacaaagcgcagatgacccgcgaccagaccacgtttgccgccgctgtggccgatctagggctgcaacggggcatcgagggcagcaaggcacgtcacacgcgcattcaggcgttctacgaggccctggagcggccaccagtgggccacgtcaccatcagcccgcaagcggtcgagccacgcgcctatgcaccgcagggattggccgaaaagctgggaatctcaaagcgcgttgagacgccggaagccgtggccgaccggctgacaaaagcggttcggcaggggtatgagcctgccctacaggccgccgcaggagcgcgtgagatgcgcaagaaggccgatcaagcccaagagacggcccgagaccttcgggagcgcctgaagcccgttctggacgccctggggccgttgaatcgggatatgcaggccaaggccgccgcgatcatcaaggccgtgggcgaaaagctgctgacggaacagcgggaagtccagcgccagaaacaggcccagcgccagcaggaacgcgggcgcgcacatttccccgaaaagtgccacctgacgtctaagaaaccattattatcatgacattaacctataaaaataggcgtatcacgaggccctttcgtcttcgaataaatacctgtgacggaagatcacttcgcagaataaataaatcctggtgtccctgttgataccgggaagccctgggccaacttttggcgaaaatgagacgttgatcggcacgtaagaggttccaactttcaccataatgaaataagatcactaccgggcgtattttttgagttatcgagattttcaggagctaaggaagctaaaatggagaaaaaaatcactggatataccaccgttgatatatcccaatggcatcgtaaagaacattttgaggcatttcagtcagttgctcaatgtacctataaccagaccgttcagctggatattacggcctttttagcttttgccattctcaccggattcagtcgtcactcatggtgatttctcacttgataaccttatttttgacgaggggaaattaataggttgtattgatgttggacgagtcggaatcgcagaccgataccaggatcttgccatcctatggaactgcctcggtgagttttctccttcattacagaaacggctttttcaaaaatatggtattgataatcctgatatgaataaattgcagtttcatttgatgctcgatgagtttttctaatcagaattggttaattggttgtaacactggcagagcattacgctgacttgacgggacggcggctttgttgaataaatcgaacttttgctgagttgaaggatcagatcacgcatcttcccgacaacgcagaccgttccgtggcaaagcaaaagttcaaaatcaccaactggtccacctacaacaaagctctcatcaaccgtggctccctcactttctggctggatgatggggcgattcaggcctggtatgagtcagcaacaccttcttcacgaggcagacctcag

pMTL71501

cctgcaggataaaaaaattgtagataaattttataaaatagttttatctacaatttttttatcaggaaacagctatgaccgcggccgctgtatccatatgaccatgattacgaattcgagctcggtacccggggatcctctagagtcgacgtcacgcgtccatggagatctcgaggcctgcagacatgcaagcttggcactggccgtcgttttacaacgtcgtgactgggaaaaccctggcgttacccaacttaatcgccttgcagcacatccccctttcgccagctggcgtaatagcgaagaggcccgcaccgatcgcccttcccaacagttgcgcagcctgaatggcgaatggcgctagcataaaaataagaagcctgcatttgcaggcttcttatttttatggcgcgccggccggcctgacagatgagacaataaccctgataaatgcttcaataatattgaaaaaggaggaagagtatggaacgaagtagcaatgaagtcagtaatccagttgctggcaattttgtattcccatcgaacgccacgtttggtatgggagatcgcgtgcgcaagaaatccggcgccgcctggcaaggtcagattgtcgggtggtactgcacaaatttgacccccgaaggctacgccgtcgagtctgaggctcacccaggctcagtacagatttatcctgttgcggcgcttgaacgcatcaactgagtttaaacgtgctacgcctgaataagtgataataagcggatgaatggcagaaattcgaaagcaaattcgacccggtcgtcggttcagggcagggtcgttaaatagccgcttatgtctattgctggtttaccggtttattgactaccggaagcagtgtgaccgtgtgcttctcaaatgcctgaggccagtttgctcaggctctccccgtggaggtaataattgacgatatgatcatttattctgcctcccagagcctgataaaaacggtgaatccgttagcgaggtgccgccggcttccattcaggtcgaggtggcccggctccatgcaccgcgacgcaacgcggggaggcagacaaggtatagggcggcgaggcggctacagccgatagtctggaacagcgcacttacgggttgctgcgcaacccaagtgctaccggcgcggcagcgtgacccgtgtcggcggctccaacggctcgccatcgtccagaaaacacggctcatcgggcatcggcaggcgctgctgcccgcgccgttcccattcctccgtttcggtcaaggctggcaggtctggttccatgcccggaatgccgggctggctgggcggctcctcgccggggccggtcggtagttgctgctcgcccggatacagggtcgggatgcggcgcaggtcgccatgccccaacagcgattcgtcctggtcgtcgtgatcaaccaccacggcggcactgaacaccgacaggcgcaactggtcgcggggctggccccacgccacgcggtcattgaccacgtaggccgacacggtgccggggccgttgagcttcacgacggagatccagcgctcggccaccaagtccttgactgcgtattggaccgtccgcaaagaacgtccgatgagcttggaaagtgtcttctggctgaccaccacggcgttctggtggcccatctgcgccacgaggtgatgcagcagcattgccgccgtgggtttcctcgcaataagcccggcccacgcctcatgcgctttgcgttccgtttgcacccagtgaccgggcttgttcttggcttgaatgccgatttctctggactgcgtggccatgcttatctccatgcggtaggggtgccgcacggttgcggcaccatgcgcaatcagctgcaacttttcggcagcgcgacaacaattatgcgttgcgtaaaagtggcagtcaattacagattttctttaacctacgcaatgagctattgcggggggtgccgcaatgagctgttgcgtaccccccttttttaagttgttgatttttaagtctttcgcatttcgccctatatctagttctttggtgcccaaagaagggcacccctgcggggttcccccacgccttcggcgcggctccccctccggcaaaaagtggcccctccggggcttgttgatcgactgcgcggccttcggccttgcccaaggtggcgctgcccccttggaacccccgcactcgccgccgtgaggctcggggggcaggcgggcgggcttcgccttcgactgcccccactcgcataggcttgggtcgttccaggcgcgtcaaggccaagccgctgcgcggtcgctgcgcgagccttgacccgccttccacttggtgtccaaccggcaagcgaagcgcgcaggccgcaggccggaggcttttccccagagaaaattaaaaaaattgatggggcaaggccgcaggccgcgcagttggagccggtgggtatgtggtcgaaggctgggtagccggtgggcaatccctgtggtcaagctcgtgggcaggcgcagcctgtccatcagcttgtccagcagggttgtccacgggccgagcgaagcgagccagccggtggccgctcgcggccatcgtccacatatccacgggctggcaagggagcgcagcgaccgcgcagggcgaagcccggagagcaagcccgtagggcgccgcagccgccgtaggcggtcacgactttgcgaagcaaagtctagtgagtatactcaagcattgagtggcccgccggaggcaccgccttgcgctgcccccgtcgagccggttggacaccaaaagggaggggcaggcatggcggcatacgcgatcatgcgatgcaagaagctggcgaaaatgggcaacgtggcggccagtctcaagcacgcctaccgcgagcgcgagacgcccaacgctgacgccagcaggacgccagagaacgagcactgggcggccagcagcaccgatgaagcgatgggccgactgcgcgagttgctgccagagaagcggcgcaaggacgctgtgttggcggtcgagtacgtcatgacggccagcccggaatggtggaagtcggccagccaagaacagcaggcggcgttcttcgagaaggcgcacaagtggctggcggacaagtacggggcggatcgcatcgtgacggccagcatccaccgtgacgaaaccagcccgcacatgaccgcgttcgtggtgccgctgacgcaggacggcaggctgtcggccaaggagttcatcggcaacaaagcgcagatgacccgcgaccagaccacgtttgccgccgctgtggccgatctagggctgcaacggggcatcgagggcagcaaggcacgtcacacgcgcattcaggcgttctacgaggccctggagcggccaccagtgggccacgtcaccatcagcccgcaagcggtcgagccacgcgcctatgcaccgcagggattggccgaaaagctgggaatctcaaagcgcgttgagacgccggaagccgtggccgaccggctgacaaaagcggttcggcaggggtatgagcctgccctacaggccgccgcaggagcgcgtgagatgcgcaagaaggccgatcaagcccaagagacggcccgagaccttcgggagcgcctgaagcccgttctggacgccctggggccgttgaatcgggatatgcaggccaaggccgccgcgatcatcaaggccgtgggcgaaaagctgctgacggaacagcgggaagtccagcgccagaaacaggcccagcgccagcaggaacgcgggcgcgcacatttccccgaaaagtgccacctgacgtctaagaaaccattattatcatgacattaacctataaaaataggcgtatcacgaggccctttcgtcttcgaataaatacctgtgacggaagatcacttcgcagaataaataaatcctggtgtccctgttgataccgggaagccctgggccaacttttggcgaaaatgagacgttgatcggcacgtaagaggttccaactttcaccataatgaaataagatcactaccgggcgtattttttgagttatcgagattttcaggagctaaggaagctaaaatggagaaaaaaatcactggatataccaccgttgatatatcccaatggcatcgtaaagaacattttgaggcatttcagtcagttgctcaatgtacctataaccagaccgttcagctggatattacggcctttttagcttttgccattctcaccggattcagtcgtcactcatggtgatttctcacttgataaccttatttttgacgaggggaaattaataggttgtattgatgttggacgagtcggaatcgcagaccgataccaggatcttgccatcctatggaactgcctcggtgagttttctccttcattacagaaacggctttttcaaaaatatggtattgataatcctgatatgaataaattgcagtttcatttgatgctcgatgagtttttctaatcagaattggttaattggttgtaacactggcagagcattacgctgacttgacgggacggcggctttgttgaataaatcgaacttttgctgagttgaaggatcagatcacgcatcttcccgacaacgcagaccgttccgtggcaaagcaaaagttcaaaatcaccaactggtccacctacaacaaagctctcatcaaccgtggctccctcactttctggctggatgatggggcgattcaggcctggtatgagtcagcaacaccttcttcacgaggcagacctcag

pMTL72111

ggataaaaaaattgtagataaattttataaaatagttttatctacaatttttttatcaggaaacagctatgaccgcggccgctgtatccatatgaccatgattacgaattcgagctcggtacccggggatcctctagagtcgacgtcacgcgtccatggagatctcgaggcctgcagacatgcaagcttggcactggccgtcgttttacaacgtcgtgactgggaaaaccctggcgttacccaacttaatcgccttgcagcacatccccctttcgccagctggcgtaatagcgaagaggcccgcaccgatcgcccttcccaacagttgcgcagcctgaatggcgaatggcgctagcataaaaataagaagcctgcatttgcaggcttcttatttttatggcgcgcctcctttttgataatctcatgaccaaaatcccttaacgtgagttttcgttccactgagcgtcagaccccgtagaaaagatcaaaggatcttcttgagatcctttttttctgcgcgtaatctgctgcttgcaaacaaaaaaaccaccgctaccagcggtggtttgtttgccggatcaagagctaccaactctttttccgaaggtaactggcttcagcagagcgcagataccaaatactgttcttctagtgtagccgtagttaggccaccacttcaagaactctgtagcaccgcctacatacctcgctctgctaatcctgttaccagtggctgctgccagtggcgataagtcgtgtcttaccgggttggactcaagacgatagttaccggataaggcgcagcggtcgggctgaacggggggttcgtgcacacagcccagcttggagcgaacgacctacaccgaactgagatacctacagcgtgagctatgagaaagcgccacgcttcccgaagggagaaaggcggacaggtatccggtaagcggcagggtcggaacaggagagcgcacgagggagcttccagggggaaacgcctggtatctttatagtcctgtcgggtttcgccacctctgacttgagcgtcgatttttgtgatgctcgtcaggggggcggagcctatggaaaaacgccagcaacgcggcctttttacggttcctggccttttgctggccttttgctcacatgttctttcctgcgttatcccctgattctgtggataaccgtattaccgcctttgagtgagctgataccgctcgccgcagccgaacgaccgagcgcagcgagtcagtgagcgaggaagcggaagagcgcccaatacgcagggcccggccggccagtgggcaagttgaaaaattcacaaaaatgtggtataatatctttgttcattagagcgataaacttgaatttgagagggaacttagatggtatttgaaaaaattgataaaaatagttggaacagaaaagagtattttgaccactactttgcaagtgtaccttgtacctacagcatgaccgttaaagtggatatcacacaaataaaggaaaagggaatgaaactatatcctgcaatgctttattatattgcaatgattgtaaaccgccattcagagtttaggacggcaatcaatcaagatggtgaattggggatatatgatgagatgataccaagctatacaatatttcacaatgatactgaaacattttccagcctttggactgagtgtaagtctgactttaaatcatttttagcagattatgaaagtgatacgcaacggtatggaaacaatcatagaatggaaggaaagccaaatgctccggaaaacatttttaatgtatctatgataccgtggtcaaccttcgatggctttaatctgaatttgcagaaaggatatgattatttgattcctatttttactatggggaaatattataaagaagataacaaaattatacttcctttggcaattcaagttcatcacgcagtatgtgacggatttcacatttgccgttttgtaaacgaattgcaggaattgataaatagttaacttcaggtttgtctgtaactaaaaacaagtatttaagcaaaaacatcgtagaaatacggtgttttttgttaccctaagtttaaactcgccttttactttaacaacgacccgtaagtaccgggggaacagctcgatctctcgggtacaacaggcacaaagttaacttgcgtatacgtctaagggccgctaaccttcacggcaacgcaaccgcggacgtcatttttgccgaaaacggttgcacgatccaccggcggttccggtgaacagcttaaaggtttttgaaccgaatggatatcaagctagcccaccccagcaagacgacgcctgaggatttgaagcagttggcaaatctctccgcggtgatgctgcagaaaattcgggatgagatgctggagccatttcctcggaaggaagccccgctgatcccgtctggccgcctacaagaattgtgtggcatcgacaaaacgcggatgaaccggtccctcaaaaagggggatctccctcagggccagcaatcgcgacccggtgcagtgcgctatttcagcctcagcgaggcaatgcaatggatccgagcggaacttaagcctgtcccgcgaaggggaccaggtaaagtcattgcagttgcgaacttcaagggcggtgtcacgaagaccactatgtccaccctcctctgccagggcttgagtctgcggcgaggtcggaaggtgtgccacgttgatctggatccgcagggaagcgcaaccacgctgtatggcatcaatccacatgccgaggtgtcgtccgaaaacaccattatgccgctcatcgaggcgtatttggcgggcgagtccttcgatatgcgagggcttcctcaggagacttactggcctaacctggatttgattccttcgtctactgagcttttcaacgcggagtttatgcttccggctcgggcgacggcagaggaaggccatattccgttcgagcgcgtgttaagtaacggcctcgattcgttgaaagacgaatatgactacatcatcctcgacacggctcctaccctcagctacctgaccatcaacgcgattttcgctgccgatggcgtcatcgtaccggtggtcccggacaccttggctttcgcgtctatggtccagttctggcaactcttctcggacctagtaacaggcatggaagagcagagcgagggatctaaaaaggagttcgactttctcgatgttctcatgacacgcatggagaaaaagaacgctcctcgcctggtggcagactggattcgcggcgtctatgggtcgcgcgtgctgccgattgagatccctgagacggacctcgcccgtaacagcagcattcaatttcgcacggtctatgacctctcctctagcgaggcgaacaccgagacgatgcgacgcattcgccaaccctgcgatgagtttgtcgactatgtggacgacaaggtcagcgcgctttggcaaggaattgaagaatgagtttgagagaaaagcttgccgcaaaggctgggaacatcaaggtcacggcggaagacttggagaaagccgctgcgcgcggtccgcaagcgccgcgaactgcgcccggtcagttaatgcatatgcaagggaaggttgagcgacaggctaacgagatcgcgcaactaagagcagaacttgagtcggcccgcgtcagcggcggcgcagtggatgtgcctatcgaccaactgcatgaggtcccaggccgcagacgcttcatgcctcccgagaagtatgtcgaattgagggaaaacctcaggcacaacaagctcgttcatcctgtgattgtatgccctcggcctgcgggaggcttcgagattgtctccgggcatcaccggacagacgcgtaccgcgagcttgggcgcgatcacatacgctgcgtgctcggcgaacttagttcagacgaggctgacacgggcgcgttctacgcgaaccttatgcagtcagatttaacggatttcgagaagtttcggaagttcgacgaactgctgcttcgcagcccagacaagactcaagccgcaatagctgaacaggctggtgtacctgtctcgactctctcagagattttgtcgttccggaacttgcctcccgaggtcctaagccttctcgatagccgcccagacctgctcgggtcgaatgctggcgccgagttggcaagggcgaccaaagacggtcgcggggatcgggtcgtcgaagcggttaagttgttggccgagaagaagatcgatcaacagcaggccgtacggatgactaaggccgagcaggttaagaccaggcctgccgcatctaccggcttcaaaatcaaggcgggaaaggcgacttggtgcgatgttcgtatcgcaaagaaagtcatgcgcattgagttccgcagcgaggaagaagcggaagcggcccaatcggccattcgcgaacatctggaagggttagctaaagctgcgtcggaagacgcaaaaagctaagtgcttgttttttaaggacttcgtactacgaatcgaggttttaagccatgtctagactgtaatcctacaaaaacaaaagcccacggcggcaaccgtgggcttttgagaacttcaagctgaccagtttcccggccgctacacaccgaagccactcgacatggattcgttagtcggagtgtagcggaacgcgaacctgagtcaagcgacttcaaccattttttacgaatgggaaggtcatatgactttcgcgcaacgctccgttgctggcgaggatgttgctcgcccacaaaaacaccttaaccagacagacgctctgattgctccggcgcccaagcgcctcaaacgcaagactatcgaagcagtcgagcgcgcaactcgaatcgtcgggatcgggcgtagcgcccgatcagctcttgccgccctcgcccgcacggcgaataacgatgaccccaccggtaagatctttaagcaccgggaaacgctttgtgccgaaaccggaatgtcgccggctacttggtaccgtgctcaacgagaactgctcgacttgggcctaattaccgtcgacgttcaagttcggaagcgatttggccgattcgcaggagcctacatttacctgacggaaaaagcgacggagatgctcggcttaagctcgcgaaaagaagaagaaaccacgggtacgggcgaggacgacacagcgcagctcggcgagccggccgttccaccaccctcttctatggcgcaaccgtctctcaaaacgagagtcctgtttacagaagatcgtgtcccatactcctttcaaaaaagacagcaggatcggctcccccaggacctgacacgtctgcgcggcctgggtcttgatgtaaatttaattttttggttgatgcgaaaggctaaagagcaaggccactttctctcagatgtcgtaagcgcgacatgggagagtcttgcgaaagcacgcgtgccaaaagcgtatctgcttgccctactcaccgcccgcaccgatttcagtgctgtctgcaaagcaaaggcactcaaagaagacaaagcccgaatccaagtgcaggaccgcgatttcgtgcgttcgatactcgcaggggcagcgcggcagtgtttcgtggacgaaaaaggcaaccatttcgaagtcgaaagcgacggaagctcagtgcttgtcaccgaggtccaaagtgcggtcacttcccgcttggtaggaacttccctcgccgaatttgcaaggcgactacacgctggtgcgtaccagaaagctgaggtctacgctgctccccagagagcaagcggccggctcgagaaacggggaaaggaggcggcttcgacgttatcggcgttgcgagcgatgctgcgcgaccgcaggtcagccaacgcggcaaacacgacgaacaatgctcatgccatggcctagggtgtgttttgcgctgaaagcctgca

pMTL73111

ggataaaaaaattgtagataaattttataaaatagttttatctacaatttttttatcaggaaacagctatgaccgcggccgctgtatccatatgaccatgattacgaattcgagctcggtacccggggatcctctagagtcgacgtcacgcgtccatggagatctcgaggcctgcagacatgcaagcttggcactggccgtcgttttacaacgtcgtgactgggaaaaccctggcgttacccaacttaatcgccttgcagcacatccccctttcgccagctggcgtaatagcgaagaggcccgcaccgatcgcccttcccaacagttgcgcagcctgaatggcgaatggcgctagcataaaaataagaagcctgcatttgcaggcttcttatttttatggcgcgcctcctttttgataatctcatgaccaaaatcccttaacgtgagttttcgttccactgagcgtcagaccccgtagaaaagatcaaaggatcttcttgagatcctttttttctgcgcgtaatctgctgcttgcaaacaaaaaaaccaccgctaccagcggtggtttgtttgccggatcaagagctaccaactctttttccgaaggtaactggcttcagcagagcgcagataccaaatactgttcttctagtgtagccgtagttaggccaccacttcaagaactctgtagcaccgcctacatacctcgctctgctaatcctgttaccagtggctgctgccagtggcgataagtcgtgtcttaccgggttggactcaagacgatagttaccggataaggcgcagcggtcgggctgaacggggggttcgtgcacacagcccagcttggagcgaacgacctacaccgaactgagatacctacagcgtgagctatgagaaagcgccacgcttcccgaagggagaaaggcggacaggtatccggtaagcggcagggtcggaacaggagagcgcacgagggagcttccagggggaaacgcctggtatctttatagtcctgtcgggtttcgccacctctgacttgagcgtcgatttttgtgatgctcgtcaggggggcggagcctatggaaaaacgccagcaacgcggcctttttacggttcctggccttttgctggccttttgctcacatgttctttcctgcgttatcccctgattctgtggataaccgtattaccgcctttgagtgagctgataccgctcgccgcagccgaacgaccgagcgcagcgagtcagtgagcgaggaagcggaagagcgcccaatacgcagggcccggccggccagtgggcaagttgaaaaattcacaaaaatgtggtataatatctttgttcattagagcgataaacttgaatttgagagggaacttagatggtatttgaaaaaattgataaaaatagttggaacagaaaagagtattttgaccactactttgcaagtgtaccttgtacctacagcatgaccgttaaagtggatatcacacaaataaaggaaaagggaatgaaactatatcctgcaatgctttattatattgcaatgattgtaaaccgccattcagagtttaggacggcaatcaatcaagatggtgaattggggatatatgatgagatgataccaagctatacaatatttcacaatgatactgaaacattttccagcctttggactgagtgtaagtctgactttaaatcatttttagcagattatgaaagtgatacgcaacggtatggaaacaatcatagaatggaaggaaagccaaatgctccggaaaacatttttaatgtatctatgataccgtggtcaaccttcgatggctttaatctgaatttgcagaaaggatatgattatttgattcctatttttactatggggaaatattataaagaagataacaaaattatacttcctttggcaattcaagttcatcacgcagtatgtgacggatttcacatttgccgttttgtaaacgaattgcaggaattgataaatagttaacttcaggtttgtctgtaactaaaaacaagtatttaagcaaaaacatcgtagaaatacggtgttttttgttaccctaagtttaaacttcttcaaattcccgttgcacatagcccggcaattcctttccctgctctgccataagcgcagcgaatgccgggtaatactcgtcaacgatctgatagagaagggtttgctcgggtcggtggctctggtaacgaccagtatcccgatcccggctggccgtcctggccgccacatgaggcatgttccgcgtccttgcaatactgtgtttacatacagcctatcgcttagcggaaagttcttttaccctcagccgaaatgcctgccgttgctagacattgccagccagtgcccgtcactcccgtactaactgtcacgaacccctgcaataactgtcacgcccccctgcaataactgtcacgaacccctgcaataactgtcacgcccccaaacctgcaaacccagcaggggcgggggctggcggggtgttggaaaaatccatccatgattatctaagaataatccactaggcgcggttatcagcgcccttgtggggcgctgctgcccttgcccaatatgcccggccagaggccggatagctggtctattcgctgcgctaggctacacaccgccccaccgctgcgcggcagggggaaaggcgggcaaagcccgctaaaccccacaccaaaccccgcagaaatacgctggagcgcttttagccgctttagcggcctttccccctacccgaagggtgggggcgcgtgtgcagccccgcagggcctgtctcggtcgatcattcagcccggctcatccttctggcgtggcggcagaccgaacaaggcgcggtcgtggtcgcgttcaaggtacgcatccattgccgccatgagccgatcctccggccactcgctgctgttcaccttggccaaaatcatggcccccaccagcaccttgcgccttgtttcgttcttgcgctcttgctgctgttcccttgcccgcacccgctgaatttcggcattgattcgcgctcgttgttcttcgagcttggccagccgatccgccgccttgttgctccccttaaccatcttgacaccccattgttaatgtgctgtctcgtaggctatcatggaggcacagcggcggcaatcccgaccctactttgtaggggagggcgcacttaccggtttctcttcgagaaactggcctaacggccacccttcgggcggtgcgctctccgagggccattgcatggagccgaaaagcaaaagcaacagcgaggcagcatggcgatttatcaccttacggcgaaaaccggcagcaggtcgggcggccaatcggccagggccaaggccgactacatccagcgcgaaggcaagtatgcccgcgacatggatgaagtcttgcacgccgaatccgggcacatgccggagttcgtcgagcggcccgccgactactgggatgctgccgacctgtatgaacgcgccaatgggcggctgttcaaggaggtcgaatttgccctgccggtcgagctgaccctcgaccagcagaaggcgctggcgtccgagttcgcccagcacctgaccggtgccgagcgcctgccgtatacgctggccatccatgccggtggcggcgagaacccgcactgccacctgatgatctccgagcggatcaatgacggcatcgagcggcccgccgctcagtggttcaagcggtacaacggcaagaccccggagaagggcggggcacagaagaccgaagcgctcaagcccaaggcatggcttgagcagacccgcgaggcatgggccgaccatgccaaccgggcattagagcgggctggccacgacgcccgcattgaccacagaacacttgaggcgcagggcatcgagcgcctgcccggtgttcacctggggccgaacgtggtggagatggaaggccggggcatccgcaccgaccgggcagacgtggccctgaacatcgacaccgccaacgcccagatcatcgacttacaggaataccgggaggcaatagaccatgaacgcaatcgacagagtgaagaaatccagaggcatcaacgagttagcggagcagatcgaaccgctggcccagagcatggcgacactggccgacgaagcccggcaggtcatgagccagacccagcaggccagcgaggcgcaggcggcggagtggctgaaagcccagcgccagacaggggcggcatgggtggagctggccaaagagttgcgggaggtagccgccgaggtgagcagcgccgcgcagagcgcccggagcgcgtcgcgggggtggcactggaagctatggctaaccgtgatgctggcttccatgatgcctacggtggtgctgctgatcgcatcgttgctcttgctcgacctgacgccactgacaaccgaggacggctcgatctggctgcgcttggtggcccgatgaagaacgacaggactttgcaggccataggccgacagctcaaggccatgggctgtgagcgcttcgatatcggcgtcagggacgccaccaccggccagatgatgaaccgggaatggtcagccgccgaagtgctccagaacacgccatggctcaagcggatgaatgcccagggcaatgacgtgtatatcaggcccgccgagcaggagcggcatggtctggtgctggtggacgacctcagcgagtttgacctggatgacatgaaagccgagggccgggagcctgccctggtagtggaaaccagcccgaagaactatcaggcatgggtcaaggtggccgacgccgcaggcggtgaacttcgggggcagattgcccggacgctggccagcgagtacgacgccgacccggccagcgccgacagccgccactatggccgcttggcgggcttcaccaaccgcaaggacaagcacaccacccgcgccggttatcagccgtgggtgctgctgcgtgaatccaagggcaagaccgccaccgctggcccggcgctggtgcagcaggctggccagcagatcgagcaggcccagcggcagcaggagaaggcccgcaggctggccagcctcgaactgcccgagcggcagcttagccgccaccggcgcacggcgctggacgagtaccgcagcgagatggccgggctggtcaagcgcttcggtgatgacctcagcaagtgcgactttatcgccgcgcagaagctggccagccggggccgcagtgccgaggaaatcggcaaggccatggccgaggccagcccagcgctggcagagcgcaagcccggccacgaagcggattacatcgagcgcaccgtcagcaaggtcatgggtctgcccagcgtccagcttgcgcgggccgagctggcacgggcaccggcaccccgccagcgaggcatggacaggggcgggccagatttcagcatgtagtgcttgcgttggtactcacgcctgttatactatgagtactcacgcacagaagggggttttatggaatacgaaaaaagcgcttcagggtcggtctacctgatcaaaagtgacaagggctattggttgcccggtggctttggttatacgtcaaacaaggccgaggctggccgcttttcagtcgctgatatggccagccttaaccttgacggctgcaccttgtccttgttccgcgaagacaagcctttcggccccggcaagtttctcggtgactgatatgaaagaccaaaaggacaagcagaccggcgacctgctggccagccctgacgctgtacgccaagcgcgatatgccgagcgcatgaaggccaaagggatgcgtcagcgcaagttctggctgaccgacgacgaatacgaggcgctgcgcgagtgcctggaagaactcagagcggcgcagggcgggggtagtgaccccgccagcgcctaaccaccaactgcctgcaaaggaggcaatcaatggctacccataagcctatcaatattctggaggcgttcgcagcagcgccgccaccgctggactacgttttgcccaacatggtggccggtacggtcggggcgctggtgtcgcccggtggtgccggtaaatccatgctggccctgcaactggccgcacagattgcaggcgggccggatctgctggaggtgggcgaactgcccaccggcccggtgatctacctgcccgccgaagacccgcccaccgccattcatcaccgcctgcacgcccttggggcgcacctcagcgccgaggaacggcaagccgtggctgacggcctgctgatccagccgctgatcggcagcctgcccaacatcatggccccggagtggttcgacggcctcaagcgcgccgccgagggccgccgcctgatggtgctggacacgctgcgccggttccacatcgaggaagaaaacgccagcggccccatggcccaggtcatcggtcgcatggaggccatcgccgccgataccgggtgctctatcgtgttcctgcaccatgccagcaagggcgcggccatgatgggcgcaggcgaccagcagcaggccagccggggcagctcggtactggtcgataacatccgctggcagtcctacctgtcgagcatgaccagcgccgaggccgaggaatggggtgtggacgacgaccagcgccggttcttcgtccgcttcggtgtgagcaaggccaactatggcgcaccgttcgctgatcggtggttcaggcggcatgacggcggggtgctcaagcccgccgtgctggagaggcagcgcaagagcaagggggtgccccgtggtgaagcctaagaacaagcacagcctcagccacgtccggcacgacccggcgcactgtctggcccccggcctgttccgtgccctcaagcggggcgagcgcaagcgcagcaagctggacgtgacgtatgactacggcgacggcaagcggatcgagttcagcggcccggagccgctgggcgctgatgatctgcgcatcctgcaagggctggtggccatggctgggcctaatggcctagtgcttggcccggaacccaagaccgaaggcggacggcagctccggctgttcctggaacccaagtgggaggccgtcaccgctgatgccatggtggtcaaaggtagctatcgggcgctggcaaaggaaatcggggcagaggtcgatagtggtggggcgctcaagcacatacaggactgcatcgagcgcctttggaaggtatccatcatcgcccagaatggccgcaagcggcaggggtttcggctgctgtcggagtacgccagcgacgaggcggacgggcgcctgtacgtggccctgaaccccttgatcgcgcaggccgtcatgggtggcggccagcatgtgcgcatcagcatggacgaggtgcgggcgctggacagcgaaaccgcccgcctgctgcaccagcggctgtgtggctggatcgaccccggcaaaaccggcaaggcttccatagataccttgtgcggctatgtctggccgtcagaggccagtggttcgaccatgcgcaagcgccgccagcgggtgcgcgaggcgttgccggagctggtcgcgctgggctggacggtaaccgagttcgcggcgggcaagtacgacatcacccggcccaaggcggcaggctgaccccccccactctattgtaaacaagacatttttatcttttatattcaatggcttattttcctgctaattggtaataccatgaaaaataccatgctcagaaaaggcttaacaatattttgaaaaattgcctactgagcgctgccgcacagctccataggccgctttcctggctttgcttccagatgtatgctcttctgctcctgca

pMTL74111

ggataaaaaaattgtagataaattttataaaatagttttatctacaatttttttatcaggaaacagctatgaccgcggccgctgtatccatatgaccatgattacgaattcgagctcggtacccggggatcctctagagtcgacgtcacgcgtccatggagatctcgaggcctgcagacatgcaagcttggcactggccgtcgttttacaacgtcgtgactgggaaaaccctggcgttacccaacttaatcgccttgcagcacatccccctttcgccagctggcgtaatagcgaagaggcccgcaccgatcgcccttcccaacagttgcgcagcctgaatggcgaatggcgctagcataaaaataagaagcctgcatttgcaggcttcttatttttatggcgcgcctcctttttgataatctcatgaccaaaatcccttaacgtgagttttcgttccactgagcgtcagaccccgtagaaaagatcaaaggatcttcttgagatcctttttttctgcgcgtaatctgctgcttgcaaacaaaaaaaccaccgctaccagcggtggtttgtttgccggatcaagagctaccaactctttttccgaaggtaactggcttcagcagagcgcagataccaaatactgttcttctagtgtagccgtagttaggccaccacttcaagaactctgtagcaccgcctacatacctcgctctgctaatcctgttaccagtggctgctgccagtggcgataagtcgtgtcttaccgggttggactcaagacgatagttaccggataaggcgcagcggtcgggctgaacggggggttcgtgcacacagcccagcttggagcgaacgacctacaccgaactgagatacctacagcgtgagctatgagaaagcgccacgcttcccgaagggagaaaggcggacaggtatccggtaagcggcagggtcggaacaggagagcgcacgagggagcttccagggggaaacgcctggtatctttatagtcctgtcgggtttcgccacctctgacttgagcgtcgatttttgtgatgctcgtcaggggggcggagcctatggaaaaacgccagcaacgcggcctttttacggttcctggccttttgctggccttttgctcacatgttctttcctgcgttatcccctgattctgtggataaccgtattaccgcctttgagtgagctgataccgctcgccgcagccgaacgaccgagcgcagcgagtcagtgagcgaggaagcggaagagcgcccaatacgcagggcccggccggccagtgggcaagttgaaaatttcacaaaaatgtggtataatatctttgttcattagagcgataaacttgaatttgagagggaacttagatggtatttgaaaaaattgataaaaatagttggaacagaaaagagtattttgaccactactttgcaagtgtaccttgtacctacagcatgaccgttaaagtggatatcacacaaataaaggaaaagggaatgaaactatatcctgcaatgctttattatattgcaatgattgtaaaccgccattcagagtttaggacggcaatcaatcaagatggtgaattggggatatatgatgagatgataccaagctatacaatatttcacaatgatactgaaacattttccagcctttggactgagtgtaagtctgactttaaatcatttttagcagattatgaaagtgatacgcaacggtatggaaacaatcatagaatggaaggaaagccaaatgctccggaaaacatttttaatgtatctatgataccgtggtcaaccttcgatggctttaatctgaatttgcagaaaggatatgattatttgattcctatttttactatggggaaatattataaagaaggtaacaaaattatacttcctttggcaattcaagttcatcacgcagtatgtgacggatttcacatttgccgttttgtaaacgaattgcaggaattgataaatagttaacttcaggtttgtctgtaactaaaaacaagtatttaagcaaaaacatcgtagaaatacggtgttttttgttaccctaagtttaaaccgatactgagcgaagcaagtgcgtcgagcagtgcccgcttgttcctgaaatgccagtaaagcgctggctgctgaacccccagccggaactgaccccacaaggccctagcgtttgcaatgcaccaggtcatcattgacccaggcgtgttccaccaggccgctgcctcgcaactcttcgcaggcttcgccgacctgctcgcgccacttcttcacgcgggtggaatccgatccgcacatgaggcggaaggtttccagcttgagcgggtacggctcccggtgcgagctgaaatagtcgaacatccgtcgggccgtcggcgacagcttgcggtacttctcccatatgaatttcgtgtagtggtcgccagcaaacagcacgacgatttcctcgtcgatcaggacctggcaacgggacgttttcttgccacggtccaggacgcggaagcagtgcagcagcgacaccgattccaggtgcccaacgcggtcggacgtgaagcccatcgccgtcgcctgtaggcgcgacaggcattcctcggccttcgtgtaataccggccattgatcgaccagcccaggtcctggcaaagctcgtagaacgtgaaggtgatcggctcgccgataggggtgcgcttcgcgtactccaacacctgctgccacaccagttcgtcatcgtcggcccgcagctcgacgccggtgtaggtgatcttcacgtccttgttgacgtggaaaatgaccttgttttgcagcgcctcgcgcgggattttcttgttgcgcgtggtgaacagggcagagcgggccgtgtcgtttggcatcgctcgcatcgtgtccggccacggcgcaatatcgaacaaggaaagctgcatttccttgatctgctgcttcgtgtgtttcagcaacgcggcctgcttggcctcgctgacctgttttgccaggtcctcgccggcggtttttcgcttcttggtcgtcatagttcctcgcgtgtcgatggtcatcgacttcgccaaacctgccgcctcctgttcgagacgacgcgaacgctccacggcggccgatggcgcgggcagggcagggggagccagttgcacgctgtcgcgctcgatcttggccgtagcttgctggaccatcgagccgacggactggaaggtttcgcggggcgcacgcatgacggtgcggcttgcgatggtttcggcatcctcggcggaaaaccccgcgtcgatcagttcttgcctgtatgccttccggtcaaacgtccgattcattcaccctccttgcgggattgccccgactcacgccggggcaatgtgcccttattcctgatttgacccgcctggtgccttggtgtccagataatccaccttatcggcaatgaagtcggtcccgtagaccgtctggccgtccttctcgtacttggtattccgaatcttgccctgcacgaataccagctccgcgaagtcgctcttcttgatggagcgcatggggacgtgcttggcaatcacgcgcaccccccggccgttttagcggctaaaaaagtcatggctctgccctcgggcggaccacgcccatcatgaccttgccaagctcgtcctgcttctcttcgatcttcgccagcagggcgaggatcgtggcatcaccgaaccgcgccgtgcgcgggtcgtcggtgagccagagtttcagcaggccgcccaggcggcccaggtcgccattgatgcgggccagctcgcggacgtgctcatagtccacgacgcccgtgattttgtagccctggccgacggccagcaggtaggcctacaggctcatgccggccgccgccgccttttcctcaatcgctcttcgttcgtctggaaggcagtacaccttgataggtgggctgcccttcctggttggcttggtttcatcagccatccgcttgccctcatctgttacgccggcggtagccggccagcctcgcagagcaggattcccgttgagcaccgccaggtgcgaataagggacagtgaagaaggaacacccgctcgcgggtgggcctacttcacctatcctgcccggctgacgccgttggatacaccaaggaaagtctacacgaaccctttggcaaaatcctgtatatcgtgcgaaaaaggatggatataccgaaaaaatcgctataatgaccccgaagcagggttatgcagcggaaaagatccgtcgaccctttccgacgctcaccgggctggttgccctcgccgctgggctggcggccgtctatggccctgcaaacgcgccagaaacgccgtcgaagccgtgtgcgagacaccgcggccgccggcgttgtggatacctcgcggaaaacttggccctcactgacagatgaggggcggacgttgacacttgaggggccgactcacccggcgcggcgttgacagatgaggggcaggctcgatttcggccggcgacgtggagctggccagcctcgcaaatcggcgaaaacgcctgattttacgcgagtttcccacagatgatgtggacaagcctggggataagtgccctgcggtattgacacttgaggggcgcgactactgacagatgaggggcgcgatccttgacacttgaggggcagagtgctgacagatgaggggcgcacctattgacatttgaggggctgtccacaggcagaaaatccagcatttgcaagggtttccgcccgtttttcggccaccgctaacctgtcttttaacctgcttttaaaccaatatttataaaccttgtttttaaccagggctgcgccctgtgcgcgtgaccgcgcacgccgaaggggggtgcccccccttctcgaaccctcccggcccgctaacgcgggcctcccatccccccaggggctgcgcccctcggccgcgaacggcctcaccccaaaaatggcagccaagctgaccacttctgcctgca

pMTL74311

caggataaaaaaattgtagataaattttataaaatagttttatctacaatttttttatcaggaaacagctatgaccgcggccgctgtatccatatgaccatgattacgaattcgagctcggtacccggggatcctctagagtcgacgtcacgcgtccatggagatctcgaggcctgcagacatgcaagcttggcactggccgtcgttttacaacgtcgtgactgggaaaaccctggcgttacccaacttaatcgccttgcagcacatccccctttcgccagctggcgtaatagcgaagaggcccgcaccgatcgcccttcccaacagttgcgcagcctgaatggcgaatggcgctagcataaaaataagaagcctgcatttgcaggcttcttatttttatggcgcgcctcctttttgataatctcatgaccaaaatcccttaacgtgagttttcgttccactgagcgtcagaccccgtagaaaagatcaaaggatcttcttgagatcctttttttctgcgcgtaatctgctgcttgcaaacaaaaaaaccaccgctaccagcggtggtttgtttgccggatcaagagctaccaactctttttccgaaggtaactggcttcagcagagcgcagataccaaatactgttcttctagtgtagccgtagttaggccaccacttcaagaactctgtagcaccgcctacatacctcgctctgctaatcctgttaccagtggctgctgccagtggcgataagtcgtgtcttaccgggttggactcaagacgatagttaccggataaggcgcagcggtcgggctgaacggggggttcgtgcacacagcccagcttggagcgaacgacctacaccgaactgagatacctacagcgtgagctatgagaaagcgccacgcttcccgaagggagaaaggcggacaggtatccggtaagcggcagggtcggaacaggagagcgcacgagggagcttccagggggaaacgcctggtatctttatagtcctgtcgggtttcgccacctctgacttgagcgtcgatttttgtgatgctcgtcaggggggcggagcctatggaaaaacgccagcaacgcggcctttttacggttcctggccttttgctggccttttgctcacatgttctttcctgcgttatcccctgattctgtggataaccgtattaccgcctttgagtgagctgataccgctcgccgcagccgaacgaccgagcgcagcgagtcagtgagcgaggaagcggaagagcgcccaatacgcagggcccggccggccgcaggagcgccagaaggccgccagagaggccgagcgcggccgtgaggcttggacgctagggcagggcatgaaaaagcccgtagcgggctgctacgggcgtctgacgcggtggaaagggggaggggatgttgtctacatggctctgctgtagtgagtgggttgcgctccggcagcggtcctgatcaatcgtcaccctttctcggtccttcaacgttcctgacaacgagcctccttttcgccaatccatcgacaatcaccgcgagtccctgctcgaacgctgcgtccggaccggcttcgtcgaaggcgtctatcgcggcccgcaacagcggcgagagcggagcctgttcaacggtgccgccgcgctcgccggcatcgctgtcgccggcctgctcctcaagcacggccccaacagtgaagtagctgattgtcatcagcgcattgacggcgtccccggccgaaaaacccgcctcgcagaggaagcgaagctgcgcgtcggccgtttccatctgcggtgcgcccggtcgcgtgccggcatggatgcgcgcgccatcgcggtaggcgagcagcgcctgcctgaagctgcgggcattcccgatcagaaatgagcgccagtcgtcgtcggctctcggcaccgaatgcgtatgattctccgccagcatggcttcggccagtgcgtcgagcagcgcccgcttgttcctgaagtgccagtaaagcgccggctgctgaacccccaaccgttccgccagtttgcgtgtcgtcagaccgtctacgccgacctcgttcaacaggtccagggcggcacggatcactgtattcggctgcaactttgtcatgcttgacactttatcactgataaacataatatgtccaccaacttatcagtgataaagaatccgcgcgttcaatcggaccagcggaggctggtccggaggccagacgtgaaacccaacatacccctgatcgtaattctgagcactgtcgcgctcgacgctgtcggcatcggcctgattatgccggtgctgccgggcctcctgcgcgatctggttcactcgaacgacgtcaccgcccactatggcattctgctggcgctgtatgcgttggtgcaatttgcctgcgcacctgtgctgggcgcgctgtcggatcgtttcgggcggcggccaatcttgctcgtctcgctggccggcgccactgtcgactacgccatcatggcgacagcgcctttcctttgggttctctatatcgggcggatcgtggccggcatcaccggggcgactggggcggtagccggcgcttatattgccgatatcactgatggcgatgagcgcgcgcggcacttcggcttcatgagcgcctgtttcgggttcgggatggtcgcgggacctgtgctcggtgggctgatgggcggtttctccccccacgctccgttcttcgccgcggcagccttgaacggcctcaatttcctgacgggctgtttccttttgccggagtcgcacaaaggcgaacgccggccgttacgccgggaggctctcaacccgctcgcttcgttccggtgggcccggggcatgaccgtcgtcgccgccctgatggcggtcttcttcatcatgcaacttgtcggacaggtgccggccgcgctttgggtcattttcggcgaggatcgctttcactgggacgcgaccacgatcggcatttcgcttgccgcatttggcattctgcattcactcgcccaggcagtgatcaccggccctgtagccgcccggctcggcgaaaggcgggcactcatgctcggaatgattgccgacggcacaggctacatcctgcttgccttcgcgacacggggatggatggcgttcccgatcatggtcctgcttgcttcgggtggcatcggaatgccggcgctgcaagcaatgttgtccaggcaggtggatgaggaacgtcaggggcagctgcaaggctcactggcggcgctcaccagcctgacctcgatcgtcggacccctcctcttcacggcgatctatgcggcttctataacaacgtggaacgggtgggcatggattgcgggcgctgccctctacttgctctgcctgccggcgctgcgtcgcgggctttggagcggcgcagggcaacgagccgatcgctgatcgtggaaacgataggaaacgtggccaatatggacaacttcttcgcccccgttttcaccatgggcaaatattacacgcaaggcgacaaggtgctgatgccgctggcgattcaggttcatcatgccgtttgtgatggcttccatgtcggcagaatgcttaatgaattacaacagtttttatgcatgcgcccaatacgcaaaccgcctctccccgcgcgttggccgattcattaatgcagctggcacgacaggtttcccgactggaaagcgggcagtgaacgcaacgcaattaatgtgagttagctcactcattaggcaccccaggctttacactttatgcttccggctcgtatgttgtgtggaattgtgagcggtttaaaccgatactgagcgaagcaagtgcgtcgagcagtgcccgcttgttcctgaaatgccagtaaagcgctggctgctgaacccccagccggaactgaccccacaaggccctagcgtttgcaatgcaccaggtcatcattgacccaggcgtgttccaccaggccgctgcctcgcaactcttcgcaggcttcgccgacctgctcgcgccacttcttcacgcgggtggaatccgatccgcacatgaggcggaaggtttccagcttgagcgggtacggctcccggtgcgagctgaaatagtcgaacatccgtcgggccgtcggcgacagcttgcggtacttctcccatatgaatttcgtgtagtggtcgccagcaaacagcacgacgatttcctcgtcgatcaggacctggcaacgggacgttttcttgccacggtccaggacgcggaagcagtgcagcagcgacaccgattccaggtgcccaacgcggtcggacgtgaagcccatcgccgtcgcctgtaggcgcgacaggcattcctcggccttcgtgtaataccggccattgatcgaccagcccaggtcctggcaaagctcgtagaacgtgaaggtgatcggctcgccgataggggtgcgcttcgcgtactccaacacctgctgccacaccagttcgtcatcgtcggcccgcagctcgacgccggtgtaggtgatcttcacgtccttgttgacgtggaaaatgaccttgttttgcagcgcctcgcgcgggattttcttgttgcgcgtggtgaacagggcagagcgggccgtgtcgtttggcatcgctcgcatcgtgtccggccacggcgcaatatcgaacaaggaaagctgcatttccttgatctgctgcttcgtgtgtttcagcaacgcggcctgcttggcctcgctgacctgttttgccaggtcctcgccggcggtttttcgcttcttggtcgtcatagttcctcgcgtgtcgatggtcatcgacttcgccaaacctgccgcctcctgttcgagacgacgcgaacgctccacggcggccgatggcgcgggcagggcagggggagccagttgcacgctgtcgcgctcgatcttggccgtagcttgctggaccatcgagccgacggactggaaggtttcgcggggcgcacgcatgacggtgcggcttgcgatggtttcggcatcctcggcggaaaaccccgcgtcgatcagttcttgcctgtatgccttccggtcaaacgtccgattcattcaccctccttgcgggattgccccgactcacgccggggcaatgtgcccttattcctgatttgacccgcctggtgccttggtgtccagataatccaccttatcggcaatgaagtcggtcccgtagaccgtctggccgtccttctcgtacttggtattccgaatcttgccctgcacgaataccagctccgcgaagtcgctcttcttgatggagcgcatggggacgtgcttggcaatcacgcgcaccccccggccgttttagcggctaaaaaagtcatggctctgccctcgggcggaccacgcccatcatgaccttgccaagctcgtcctgcttctcttcgatcttcgccagcagggcgaggatcgtggcatcaccgaaccgcgccgtgcgcgggtcgtcggtgagccagagtttcagcaggccgcccaggcggcccaggtcgccattgatgcgggccagctcgcggacgtgctcatagtccacgacgcccgtgattttgtagccctggccgacggccagcaggtaggcctacaggctcatgccggccgccgccgccttttcctcaatcgctcttcgttcgtctggaaggcagtacaccttgataggtgggctgcccttcctggttggcttggtttcatcagccatccgcttgccctcatctgttacgccggcggtagccggccagcctcgcagagcaggattcccgttgagcaccgccaggtgcgaataagggacagtgaagaaggaacacccgctcgcgggtgggcctacttcacctatcctgcccggctgacgccgttggatacaccaaggaaagtctacacgaaccctttggcaaaatcctgtatatcgtgcgaaaaaggatggatataccgaaaaaatcgctataatgaccccgaagcagggttatgcagcggaaaagatccgtcgaccctttccgacgctcaccgggctggttgccctcgccgctgggctggcggccgtctatggccctgcaaacgcgccagaaacgccgtcgaagccgtgtgcgagacaccgcggccgccggcgttgtggatacctcgcggaaaacttggccctcactgacagatgaggggcggacgttgacacttgaggggccgactcacccggcgcggcgttgacagatgaggggcaggctcgatttcggccggcgacgtggagctggccagcctcgcaaatcggcgaaaacgcctgattttacgcgagtttcccacagatgatgtggacaagcctggggataagtgccctgcggtattgacacttgaggggcgcgactactgacagatgaggggcgcgatccttgacacttgaggggcagagtgctgacagatgaggggcgcacctattgacatttgaggggctgtccacaggcagaaaatccagcatttgcaagggtttccgcccgtttttcggccaccgctaacctgtcttttaacctgcttttaaaccaatatttataaaccttgtttttaaccagggctgcgccctgtgcgcgtgaccgcgcacgccgaaggggggtgcccccccttctcgaaccctcccggcccgctaacgcgggcctcccatccccccaggggctgcgcccctcggccgcgaacggcctcaccccaaaaatggcagccaagctgaccacttctgcctg

pMTL75111

ggataaaaaaattgtagataaattttataaaatagttttatctacaatttttttatcaggaaacagctatgaccgcggccgctgtatccatatgaccatgattacgaattcgagctcggtacccggggatcctctagagtcgacgtcacgcgtccatggagatctcgaggcctgcagacatgcaagcttggcactggccgtcgttttacaacgtcgtgactgggaaaaccctggcgttacccaacttaatcgccttgcagcacatccccctttcgccagctggcgtaatagcgaagaggcccgcaccgatcgcccttcccaacagttgcgcagcctgaatggcgaatggcgctagcataaaaataagaagcctgcatttgcaggcttcttatttttatggcgcgcctcctttttgataatctcatgaccaaaatcccttaacgtgagttttcgttccactgagcgtcagaccccgtagaaaagatcaaaggatcttcttgagatcctttttttctgcgcgtaatctgctgcttgcaaacaaaaaaaccaccgctaccagcggtggtttgtttgccggatcaagagctaccaactctttttccgaaggtaactggcttcagcagagcgcagataccaaatactgttcttctagtgtagccgtagttaggccaccacttcaagaactctgtagcaccgcctacatacctcgctctgctaatcctgttaccagtggctgctgccagtggcgataagtcgtgtcttaccgggttggactcaagacgatagttaccggataaggcgcagcggtcgggctgaacggggggttcgtgcacacagcccagcttggagcgaacgacctacaccgaactgagatacctacagcgtgagctatgagaaagcgccacgcttcccgaagggagaaaggcggacaggtatccggtaagcggcagggtcggaacaggagagcgcacgagggagcttccagggggaaacgcctggtatctttatagtcctgtcgggtttcgccacctctgacttgagcgtcgatttttgtgatgctcgtcaggggggcggagcctatggaaaaacgccagcaacgcggcctttttacggttcctggccttttgctggccttttgctcacatgttctttcctgcgttatcccctgattctgtggataaccgtattaccgcctttgagtgagctgataccgctcgccgcagccgaacgaccgagcgcagcgagtcagtgagcgaggaagcggaagagcgcccaatacgcagggcccggccggccagtgggcaagttgaaaaattcacaaaaatgtggtataatatctttgttcattagagcgataaacttgaatttgagagggaacttagatggtatttgaaaaaattgataaaaatagttggaacagaaaagagtattttgaccactactttgcaagtgtaccttgtacctacagcatgaccgttaaagtggatatcacacaaataaaggaaaagggaatgaaactatatcctgcaatgctttattatattgcaatgattgtaaaccgccattcagagtttaggacggcaatcaatcaagatggtgaattggggatatatgatgagatgataccaagctatacaatatttcacaatgatactgaaacattttccagcctttggactgagtgtaagtctgactttaaatcatttttagcagattatgaaagtgatacgcaacggtatggaaacaatcatagaatggaaggaaagccaaatgctccggaaaacatttttaatgtatctatgataccgtggtcaaccttcgatggctttaatctgaatttgcagaaaggatatgattatttgattcctatttttactatggggaaatattataaagaaggtaacaaaattatacttcctttggcaattcaagttcatcacgcagtatgtgacggatttcacatttgccgttttgtaaacgaattgcaggaattgataaatagttaacttcaggtttgtctgtaactaaaaacaagtatttaagcaaaaacatcgtagaaatacggtgttttttgttaccctaagtttaaacatgaaaccgcaccaggacggccaggacgaaccgtttttcattaccgaagagatcgaggcggagatgatcgcggccgggtacgtgttcgagccgcccgcgcacgtctcaaccgtgcggctgcatgaaatcctggccggtttgtctgatgccaagctggcggcctggccggccagcttggccgctgaagaaaccgagcgccgccgtctaaaaaggtgatgtgtatttgagtaaaacagcttgcgtcatgcggtcgctgcgtatatgatgcgatgagtaaataaacaaatacgcaaggggaacgcatgaaggttatcgctgtacttaaccagaaaggcgggtcaggcaagacgaccatcgcaacccatctagcccgcgccctgcaactcgccggggccgatgttctgttagtcgattccgatccccagggcagtgcccgcgattgggcggccgtgcgggaagatcaaccgctaaccgttgtcggcatcgaccgcccgacgattgaccgcgacgtgaaggccatcggccggcgcgacttcgtagtgatcgacggagcgccccaggcggcggacttggctgtgtccgcgatcaaggcagccgacttcgtgctgattccggtgcagccaagcccttacgacatatgggccaccgccgacctggtggagctggttaagcagcgcattgaggtcacggatggaaggctacaagcggcctttgtcgtgtcgcgggcgatcaaaggcacgcgcatcggcggtgaggttgccgaggcgctggccgggtacgagctgcccattcttgagtcccgtatcacgcagcgcgtgagctacccaggcactgccgccgccggcacaaccgttcttgaatcagaacccgagggcgacgctgcccgcgaggtccaggcgctggccgctgaaattaaatcaaaactcatttgagttaatgaggtaaagaggaaatgagcaaaagcacaaacacgctaagtgccggccgtccgagcgcacgcagcagcaaggctgcaacgttggccagcctggcagacacgccagccatgaagcgggtcaactttcagttgtcggcggaggatcacaccaagctgaagatgtacgcggtacgccaaggcaagaccattaccgagctgctatctgaatacatcgcgcagctaccagagtaaatgagcaaatgaataatggagtagatgaattttagcggctaaaggaggcggcatggaaaatcaagaacaaccaggcaccgacgccgtggaatgccccatgtgtggaggaacgggcggttggccaggcgtaagcggctgggttgtctgccggccctgcaatggcactggaacccccaagcccgaggaatcggcgtgagcggtcgcaaaccatccggcccggtacaaatcggcgcggcgctgggtgatgacctggtggagaagttgaaggccgcgcaggccgcccagcggcaacgcatcgaggcagaagcacgccccggtgaatcgtggcaagcggccgctgatcgaatccgcaaagaatcccggcaaccgccggcagccggtgcgccgtcgattaggaagccgcccaagggcgacgagcaaccagattttttcgttccgatgctctatgacgtgggcacccgcgatagtcgcagcatcatggacgtggccgttttccgtctgtcgaagcgtgaccgacgagctggcgaggtgatccgctacgagcttccagacgggcacgtagaggtttccgcagggccggccggcatggccagtgtgtgggattacgacctggtactgatggcggtttcccatctaaccgaatccatgaaccgataccgggaagggaagggagacaagcccggccgcgtgttccgtccacacgttgcggacgtactcaagttctgccggcgagccgatggcggaaagcagaaagacgacctggtagaaacctgcattcggttaaacaccacgcacgttgccatgcagcgtacgaagaaggccaagaacggccgcctggtgacggtatccgagggtgaagccttgattagccgctacaagatcgtaaagagcgaaaccgggcggccggagtacatcgagatcgagctagctgattggatgtaccgcgagatcacagaaggcaagaacccggacgtgctgacggttcaccccgattactttttgatcgatcccggcatcggccgttttctctaccgcctggcacgccgcgccgcaggcaaggcagaagccagatggttgttcaagacgatctacgaacgcagtggcagcgccggagagttcaagaagttctgtttcaccgtgcgcaagctgatcgggtcaaatgacctgccggagtacgatttgaaggaggaggcggggcaggctggcccgatcctagtcatgcgctaccgcaacctgatcgagggcgaagcatccgccggttcctaatgtacggagcagatgctagggcaaattgccctagcaggggaaaaaggtcgaaaaggtctctttcctgtggatagcacgtacattgggaacccaaagccgtacattgggaaccggaacccgtacattgggaacccaaagccgtacattgggaaccggccacacatgtaagtgactgatataaaagagaaaaaaggcgatttttccgcctaaaactctttaaaacttattaaaactcttaaaacccgcctggcctgtgcataactgtctggccagcgcacagccgaagagctgcaaaaagcgcctacccttcggtcgctgcgctccctacgccccgccgcttcgcgtcggcctatcgcggccgctggccgctcaaaaatggctggcctacggccaggcaatctaccagggcgcggacaagccgcgccgtcgccactcgaccgccggcgctcctgca

pMTL70111

cctgcaggataaaaaaattgtagataaattttataaaatagttttatctacaatttttttatcaggaaacagctatgaccgcggccgctgtatccatatgaccatgattacgaattcgagctcggtacccggggatcctctagagtcgacgtcacgcgtccatggagatctcgaggcctgcagacatgcaagcttggcactggccgtcgttttacaacgtcgtgactgggaaaaccctggcgttacccaacttaatcgccttgcagcacatccccctttcgccagctggcgtaatagcgaagaggcccgcaccgatcgcccttcccaacagttgcgcagcctgaatggcgaatggcgctagcataaaaataagaagcctgcatttgcaggcttcttatttttatggcgcgcctcctttttgataatctcatgaccaaaatcccttaacgtgagttttcgttccactgagcgtcagaccccgtagaaaagatcaaaggatcttcttgagatcctttttttctgcgcgtaatctgctgcttgcaaacaaaaaaaccaccgctaccagcggtggtttgtttgccggatcaagagctaccaactctttttccgaaggtaactggcttcagcagagcgcagataccaaatactgttcttctagtgtagccgtagttaggccaccacttcaagaactctgtagcaccgcctacatacctcgctctgctaatcctgttaccagtggctgctgccagtggcgataagtcgtgtcttaccgggttggactcaagacgatagttaccggataaggcgcagcggtcgggctgaacggggggttcgtgcacacagcccagcttggagcgaacgacctacaccgaactgagatacctacagcgtgagctatgagaaagcgccacgcttcccgaagggagaaaggcggacaggtatccggtaagcggcagggtcggaacaggagagcgcacgagggagcttccagggggaaacgcctggtatctttatagtcctgtcgggtttcgccacctctgacttgagcgtcgatttttgtgatgctcgtcaggggggcggagcctatggaaaaacgccagcaacgcggcctttttacggttcctggccttttgctggccttttgctcacatgttctttcctgcgttatcccctgattctgtggataaccgtattaccgcctttgagtgagctgataccgctcgccgcagccgaacgaccgagcgcagcgagtcagtgagcgaggaagcggaagagcgcccaatacgcagggcccggccggccagtgggcaagttgaaaaattcacaaaaatgtggtataatatctttgttcattagagcgataaacttgaatttgagagggaacttagatggtatttgaaaaaattgataaaaatagttggaacagaaaagagtattttgaccactactttgcaagtgtaccttgtacctacagcatgaccgttaaagtggatatcacacaaataaaggaaaagggaatgaaactatatcctgcaatgctttattatattgcaatgattgtaaaccgccattcagagtttaggacggcaatcaatcaagatggtgaattggggatatatgatgagatgataccaagctatacaatatttcacaatgatactgaaacattttccagcctttggactgagtgtaagtctgactttaaatcatttttagcagattatgaaagtgatacgcaacggtatggaaacaatcatagaatggaaggaaagccaaatgctccggaaaacatttttaatgtatctatgataccgtggtcaaccttcgatggctttaatctgaatttgcagaaaggatatgattatttgattcctatttttactatggggaaatattataaagaagataacaaaattatacttcctttggcaattcaagttcatcacgcagtatgtgacggatttcacatttgccgttttgtaaacgaattgcaggaattgataaatagttaacttcaggtttgtctgtaactaaaaacaagtatttaagcaaaaacatcgtagaaatacggtgttttttgttaccctaagtttaaac

pMTL70131

cctgcaggataaaaaaattgtagataaattttataaaatagttttatctacaatttttttatcaggaaacagctatgaccgcggccgctgtatccatatgaccatgattacgaattcgagctcggtacccggggatcctctagagtcgacgtcacgcgtccatggagatctcgaggcctgcagacatgcaagcttggcactggccgtcgttttacaacgtcgtgactgggaaaaccctggcgttacccaacttaatcgccttgcagcacatccccctttcgccagctggcgtaatagcgaagaggcccgcaccgatcgcccttcccaacagttgcgcagcctgaatggcgaatggcgctagcataaaaataagaagcctgcatttgcaggcttcttatttttatggcgcgccaagatgatcttcttgagatcgttttggtctgcgcgtaatctcttgctctgaaaacgaaaaaaccgccttgcagggcggtttttcgaaggttctctgagctaccaactctttgaaccgaggtaactggcttggaggagcgcagtcaccaaaacttgtcctttcagtttagccttaaccggcgcatgacttcaagactaactcctctaaatcaattaccagtggctgctgccagtggtgcttttgcatgtctttccgggttggactcaagacgatagttaccggataaggcgcagcggtcggactgaacggggggttcgtgcatacagtccagcttggagcgaactgcctacccggaactgagtgtcaggcgtggaatgagacaaacgcggccataacagcggaatgacaccggtaaaccgaaaggcaggaacaggagagcgcacgagggagccgccagggggaaacgcctggtatctttatagtcctgtcgggtttcgccaccactgatttgagcgtcagatttcgtgatgcttgtcaggggggcggagcctatggaaaaacggctttgccgcggccctctcacttccctgttaagtatcttcctggcatcttccaggaaatctccgccccgttcgtaagccatttccgctcgccgcagtcgaacgaccgagcgtagcgagtggccggccagtgggcaagttgaaaaattcacaaaaatgtggtataatatctttgttcattagagcgataaacttgaatttgagagggaacttagatggtatttgaaaaaattgataaaaatagttggaacagaaaagagtattttgaccactactttgcaagtgtaccttgtacctacagcatgaccgttaaagtggatatcacacaaataaaggaaaagggaatgaaactatatcctgcaatgctttattatattgcaatgattgtaaaccgccattcagagtttaggacggcaatcaatcaagatggtgaattggggatatatgatgagatgataccaagctatacaatatttcacaatgatactgaaacattttccagcctttggactgagtgtaagtctgactttaaatcatttttagcagattatgaaagtgatacgcaacggtatggaaacaatcatagaatggaaggaaagccaaatgctccggaaaacatttttaatgtatctatgataccgtggtcaaccttcgatggctttaatctgaatttgcagaaaggatatgattatttgattcctatttttactatggggaaatattataaagaagataacaaaattatacttcctttggcaattcaagttcatcacgcagtatgtgacggatttcacatttgccgttttgtaaacgaattgcaggaattgataaatagttaacttcaggtttgtctgtaactaaaaacaagtatttaagcaaaaacatcgtagaaatacggtgttttttgttaccctaagtttaaac

pMTL70621

cctgcaggataaaaaaattgtagataaattttataaaatagttttatctacaatttttttatcaggaaacagctatgaccgcggccgctgtatccatatgaccatgattacgaattcgagctcggtacccggggatcctctagagtcgacgtcacgcgtccatggagatctcgaggcctgcagacatgcaagcttggcactggccgtcgttttacaacgtcgtgactgggaaaaccctggcgttacccaacttaatcgccttgcagcacatccccctttcgccagctggcgtaatagcgaagaggcccgcaccgatcgcccttcccaacagttgcgcagcctgaatggcgaatggcgctagcataaaaataagaagcctgcatttgcaggcttcttatttttatggcgcgcccggtcagttcagtaatttcctgcatttgcctgtttccagtcggtagatattccacaaaacagcagggaagcagcgcttttccgctgcataaccctgcttcggggtcattatagcgattttttcggtatatccatcctttttcgcacgatatacaggattttgccaaagggttcgtgtagactttccttggtgtatccaacggcgtcagccgggcaggataggtgaagtaggcccacccgcgagcgggtgttccttcttcactgtcccttattcgcacctggcggtgctcaacgggaatcctgctctgcgaggctggccggctaccgccggcgtaacagatgagggcaagcggatggctgatgaaaccaagccaaccaggaagggcagcccacctatcaaggtgtactgccttccagacgaacgaagagcgattgaggaaaaggcggcggcggccccggatctatgcggtgtgaaataccgcacagatgcgtaaggagaaaataccgcatcaggcgctcttccgcttcctcgctcactgactcgctgcgctcggtcgttcggctgcggcgagcggtatcagctcactcaaaggcggtaatacggttatccacagaatcaggggataacgcaggaaagaacatgtgagcaaaaggccagcaaaaggccaggaaccgtaaaaaggccgcgttgctggcgtttttccataggctccgcccccctgacgagcatcacaaaaatcgacgctcaagtcagaggtggcgaaacccgacaggactataaagataccaggcgtttccccctggaagctccctcgtgcgctctcctgttccgaccctgccgcttaccggatacctgtccgcctttctcccttcgggaagcgtggcgctttctcatagctcacgctgtaggtatctcagttcggtgtaggtcgttcgctccaagctgggctgtgtgcacgaaccccccgttcagcccgaccgctgcgccttatccggtaactatcgtcttgagtccaacccggtaagacacgacttatcgccactggcagcagccactggtaacaggattagcagagcgaggtatgtaggcggtgctacagagttcttgaagtggtggcctaactacggctacactagaaggacagtatttggtatctgcgctctgctgaagccagttaccttcggaaaaagagttggtagctcttgatccggcaaacaaaccaccgctggtagcggtggtttttttgtttgcaagcagcagattacgcgcagaaaaaaaggatctcaagaagatcctttgatcttttctacggggtctgacgctcagtggaacgaaaactcacgttaagggattttggtcatgagattatcaaaaaggatcttcacctagatccggccggcctaattctcatgtttgacagcttatcatcgataagctttaatgcggtagtttatcacagttaaattgctaacgcagtcaggcaccgtgtatgaaatctaacaatgcgctcatcgtcatcctcggcaccgtcaccctggatgctgtaggcataggcttggttatgccggtactgccgggcctcttgcgggatatcgtccattccgacagcatcgccagtcactatggcgtgctgctagcgctatatgcgttgatgcaatttctatgcgcacccgttctcggagcactgtccgaccgctttggccgccgcccagtcctgctcgcttcgctacttggagccactatcgactacgcgatcatggcgaccacacccgtcctgtggatcctctacgccggacgcatcgtggccggcatcaccggcgccacaggtgcggttgctggcgcctatatcgccgacatcaccgatggggaagatcgggctcgccacttcgggctcatgagcgcttgtttcggcgtgggtatggtggcaggccccgtggccgggggactgttgggcgccatctccttgcatgcaccattccttgcggcggcggtgctcaacggcctcaacctactactgggctgcttcctaatgcaggagtcgcataagggagagcgtcgaccgatgcccttgagagccttcaacccagtcagctccttccggtgggcgcggggcatgactatcgtcgccgcacttatgactgtcttctttatcatgcaactcgtaggacaggtgccggcagcgctctgggtcattttcggcgaggaccgctttcgctggagcgcgacgatgatcggcctgtcgcttgcggtattcggaatcttgcacgccctcgctcaagccttcgtcactggtcccgccaccaaacgtttcggcgagaagcaggccattatcgccggcatggcggccgacgcgctgggctacgtcttgctggcgttcgcgacgcgaggctggatggccttccccattatgattcttctcgcttccggcggcatcgggatgcccgcgttgcaggccatgctgtccaggcaggtagatgacgaccatcagggacagcttcaaggatcgctcgcggctcttaccagcctaacttcgatcattggaccgctgatcgtcacggcgatttatgccgcctcggcgagcacatggaacgggttggcatggattgtaggcgccgccctataccttgtctgcctccccgcgttgcgtcgcggtgcatggagccgggccacctcgacctgaatggaagccggcggcacctcgctaacggattcaccactccaagaattggagccaatcaattcttgcggagaactgtgaatgcgcaaaccaacccgtttaaac
